# Practical electrochemical hydrogenation of nitriles at the nickel foam cathode†

**DOI:** 10.1039/d4gc03446e

**Published:** 2024-09-05

**Authors:** Rok Narobe, Marcel Nicolas Perner, María de Jesús Gálvez-Vázquez, Conrad Kuhwald, Martin Klein, Peter Broekmann, Sina Rösler, Bertram Cezanne, Siegfried R. Waldvogel

**Affiliations:** a Department of Chemistry, Johannes Gutenberg University Mainz 55128 Mainz Germany; b Max-Planck-Institute for Chemical Energy Conversion Stiftstraße 34–36 45470 Mülheim an der Ruhr Germany siegfried.waldvogel@cec.mpg.de +49 208/306-3131; c Sigma-Aldrich Production GmbH 9470 Buchs Switzerland; d Merck Electronics KGaA 64293 Darmstadt Germany; e Department of Chemistry, Biochemistry and Pharmaceutical Sciences, University of Bern 3012 Bern Switzerland

## Abstract

We report a scalable hydrogenation method for nitriles based on cost-effective materials in a very simple two-electrode setup under galvanostatic conditions. All components are commercially and readily available. The method is very easy to conduct and applicable to a variety of nitrile substrates, leading exclusively to primary amine products in yields of up to 89% using an easy work-up protocol. Importantly, this method is readily transferable from the milligram scale in batch-type screening cells to the multi-gram scale in a flow-type electrolyser. The transfer to flow electrolysis enabled us to achieve a notable 20 g day^−1^ productivity of phenylethylamine at a geometric current density of 50 mA cm^−2^ in a flow-type electrolyser with 48 cm^2^ electrodes. It is noteworthy that this method is sustainable in terms of process safety and reusability of components.

## Abstract 2

We report a scalable hydrogenation method for nitriles based on cost-effective materials in a very simple two-electrode setup under galvanostatic conditions.

## Graphical abstract

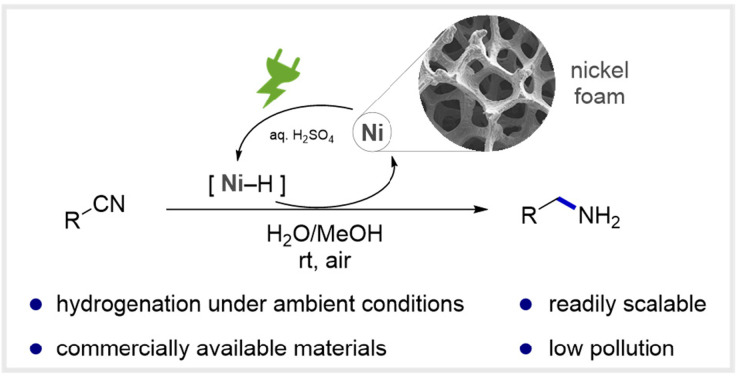

## Introduction

Hydrogenations are fundamental reactions in organic chemistry typically achieved by using either stoichiometric reagents^1,2^ or simply hydrogen gas in combination with a metal catalyst.^3–6^ Despite many positive attributes of catalytic hydrogenation methods, significant safety risks are associated with the supply chain of compressed, flammable, potentially explosive hydrogen gas (production, transportation, and storage) and its handling in a research lab or industrial production plant.^7^ In this context, electrochemistry offers a neat solution,^8–14^ as it allows the reduction of protons (H^+^) on a metal cathode material (M) leading to hydrogen adsorbed onto a metal surface (M–H), which is an essential intermediate in hydrogenation reactions.^15^ This safer and more sustainable alternative approach recently attracted considerable interest with many successful examples,^16,17^ especially in the field of biomass derived organics.^18,19^ Among the most frequently hydrogenated functionalities are multiple bonds and carbonyls, whereas reports of hydrogenation of nitriles are comparably rare.^16,20^ Nevertheless, the products of the latter, primary amines, are exceptionally important as versatile intermediates for the production of value-added fine and bulk chemicals in the plastic,^21^ pharmaceutical,^22^ and agrochemical industries.^23^

**Scheme 1:**
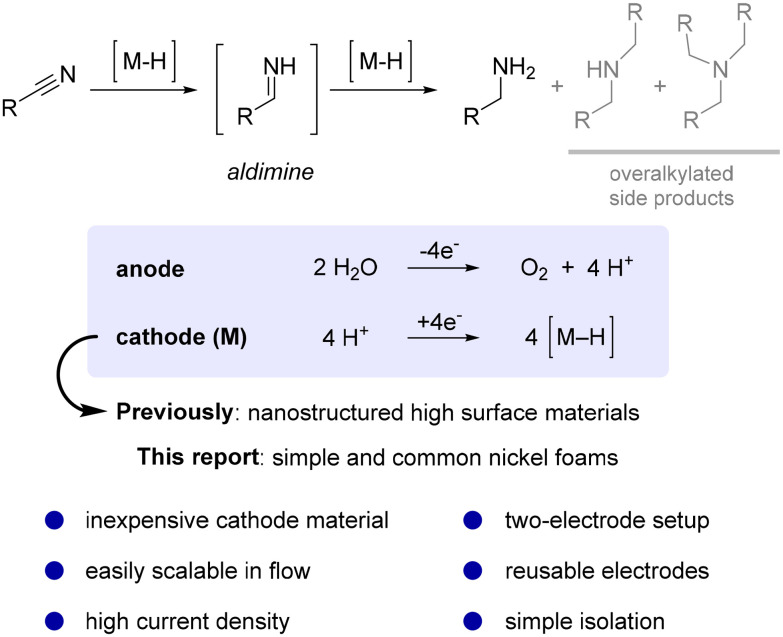
Application of nickel foam for electrochemical hydrogenation of nitriles.

The hydrogenation of nitriles to the corresponding primary amines is a challenging two-step process usually performed under high pressure. It proceeds *via* the formation of a reactive aldimine intermediate (Scheme 1),^24–27^ which is prone to side reactions such as attack of nucleophilic amines, which results in the formation of undesired overalkylated side products.^28,29^ Moreover, strongly coordinating amines may lead to the deactivation of metal hydrogenation catalytic sites.^30,31^

Promising results have been achieved using specially developed nanostructured cathode materials based on iron,^32^ nickel,^33^ or copper.^34–37^ By careful tuning of reaction parameters and additives in combination with the use of a relatively complex three-electrode reaction setup, side reactions including hydrogen evolution can be efficiently suppressed. Despite the impressive selectivity, applications on a relevant preparative scale remain elusive mainly due to the technical difficulties associated with the manufacturing of these electrodes in large dimensions,^38^ their limited mechanical stability,^39^ and the possible disintegration of such a sophisticated surface under prolonged operation.^40^

A different, more robust strategy for improving reaction selectivity towards primary amine formation relies on suppressing the nucleophilicity of amine products by simple protonation under acidic reaction conditions.^41^ These reactions can be performed under constant current electrolysis conditions as the system is less prone to any side reactions apart from hydrogen evolution. In addition, the acidic medium provides superb ionic conductivity which is required for electrolysis.^42,43^ This more synthetically oriented electrolysis approach typically offers faster conversion and, importantly, allows the application of a more convenient two-electrode reaction setup.^44^ Representative reports include hydrogenation on highly porous palladium,^45^ iron black,^46^ cobalt black,^46^ or pyrophoric RANEY® nickel cathodes.^47^

Despite all the impressive developments in the field of electrochemical hydrogenations, industrial scale applications are still lagging behind due to a lack of accessible cathode materials.^16,38^ The absence of commercial electrode sources along with the non-trivial tedious preparation of advanced high surface materials precludes more widespread use of inherently safer electrochemical hydrogenation methods.^48^ To address this need in preparative electrolysis, we present a convenient nitrile hydrogenation based on a readily and commercially available inexpensive nickel foam cathode material in a two-electrode setup under galvanostatic conditions. The use of simple nickel foam represents a central advantage for the method as it is readily and commercially available and can be purchased as large sheets. Owing to its excellent mechanical properties and electrical conductivity, any foreseeable large-scale application in flow reactors should become significantly easier compared to using alternative cathode materials.^49–51^ To support this claim, we herein present a successful development of nitrile reduction from the milligram scale in screening-type cells to multi-gram preparative hydrogenation in a flow electrolyser along with promising reusability studies of the nickel electrode.

## Results and discussion

### Reaction development

Inspired by the superb reduction properties of classic RANEY® nickel reagents and promising literature indications of well-balanced properties between hydrogen adsorption and evolution, safety, availability, and recyclability of nickel-based electrodes,^20,52–55^ we tested nickel foam as a cathode material for hydrogenations of our test substrate benzylcyanide (1a). The selected substrate is challenging as it contains an unconjugated nitrile group, yet interesting as the desired product, 2-phenylethylamine, represents an important class of pharmaceutically active compounds.^22^ Sulfuric acid was used as an electrolyte and the initial source of hydrogen atoms in well conducting aqueous medium.^42^ In the catholyte, additional methanol was required to ensure the solubility of the organic substrate 1a. To prevent undesired anodic oxidative degradation of the product,^56^ we worked in a divided cell setup using a sulfonic acid-based cation exchange membrane (Nafion™ N324) as a separator. Indeed, after some optimization (Tables S1–S3†),^57^ we found galvanostatic conditions that furnish the desired product in 91% yield (Table 1, entry 1) under ambient conditions. Using a nickel plate instead of nickel foam as a cathode resulted in only traces of the product (entry 2), highlighting the importance of the large surface area of the foam material. Lowering the concentration of sulfuric acid decreased the product yield as well (entry 3), and more importantly, led to visual changes in the appearance of the foam material (black staining). Due to the observed lower stability of nickel foam in less acidic electrolytes, all attempts to decrease the ratio between the acid and substrate 1a were discontinued.

**Table 1:** Electrochemical hydrogenation of benzylcyanide^a^

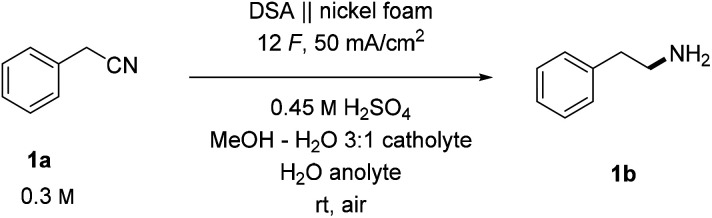		
Entry	Deviation	Yield [%]
1	None	91 (88)^b^
2	Nickel plate instead of nickel foam	12
3	Less H_2_SO_4_ (0.15 M)	69
4	Graphite as the anode	96
5	Undivided cell	31
6	1 atm H_2_ without electricity	<5

a Reaction conditions: anolyte 3.15 mmol H_2_SO_4_ in 7 mL H_2_O, catholyte 2.1 mmol benzylcyanide and 3.15 mmol H_2_SO_4_ in 7 mL of MeOH : H_2_O (3 : 1). Reactions performed using the IrO_2_ DSA anode and the nickel foam cathode, 50 mA cm^−2^, 12 *F* in a divided cell (Nafion™ N324) at rt under air. Reported current densities are calculated based on the geometric area of the electrode. GC yields were determined using tripropylamine as an internal standard.

b Isolated yield.

In the anodic compartment, water oxidation is used as a clean counter reaction evolving oxygen (O_2_) and proton flux.^58^ A dimensionally stable anode (DSA) based on iridium oxide was used for this purpose due to its low overpotential for water oxidation and proven superior stability under acidic conditions.^59,60^ Alternatively, graphite can be used as a metal-free anode material giving an even higher product yield of 96% (entry 4). However, because of its fast decomposition and foreseeable issues in the scale-up, we decided to stay with DSA electrodes. A metal-free boron-doped diamond (BDD) anode also showed good stability and similarly high yield (95%, see Table S1†). However, its high overpotential for water oxidation, high costs and formation of peroxide species make it a less appealing option.^61,62^ The results obtained in an undivided cell, as expected, showed many by-products and incomplete conversion of the substrate (entry 5). A control reaction under a hydrogen atmosphere without applying electric current did not lead to effective product formation (entry 6).

### Synthetic application

**Scheme 2:**
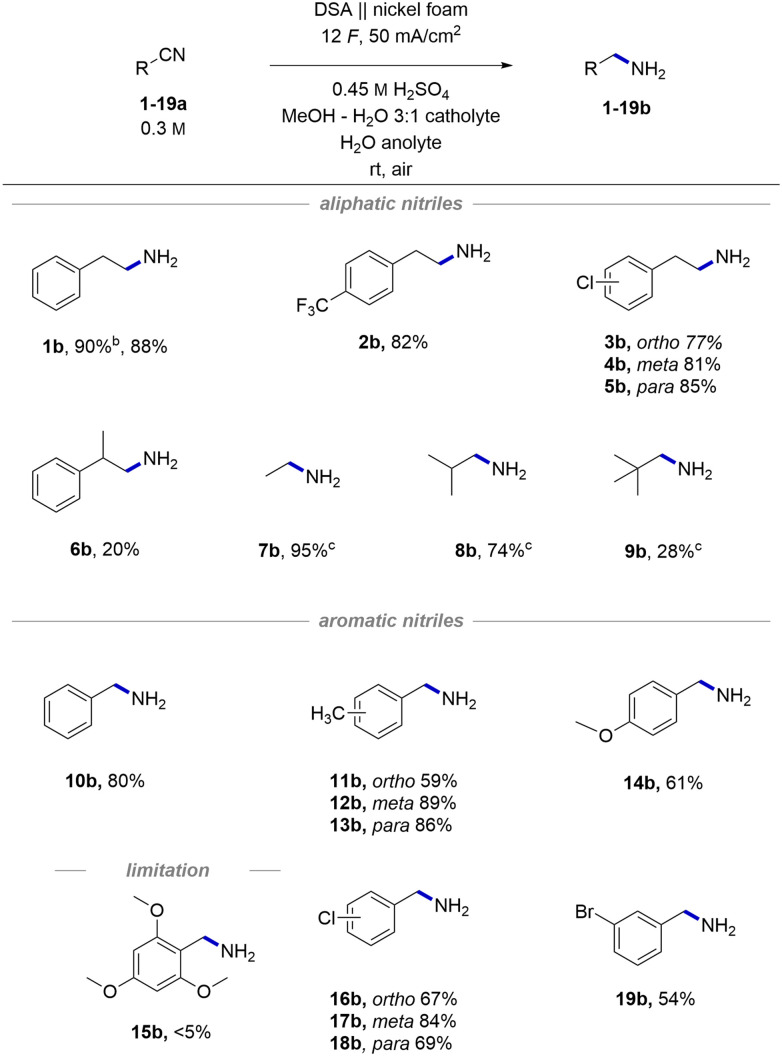
Synthetic scope of the developed electrochemical hydrogenation. Reaction conditions: anolyte 3.15 mmol H_2_SO_4_ in 7 mL H_2_O, catholyte 2.1 mmol substrate and 3.15 mmol H_2_SO_4_ in 7 mL of MeOH : H_2_O (3 : 1). Reactions performed using the IrO_2_ DSA anode and the nickel foam cathode, 50 mA cm^−2^, 12 *F* in a divided cell (Nafion™ N324) at rt under air. Reported current densities are calculated based on the geometric area of the electrode. Reported are isolated yields of HCl salts. ^a^Yield of the free amine before precipitation as an HCl salt. ^b1^H NMR yield using *N*,*N*-dimethylformamide as an internal standard.

With the optimized conditions in hand, we started exploring the general applicability of the transformation by testing different aliphatic and aromatic nitriles, obtaining the corresponding amines (1–19b) displayed in Scheme 2. To expand the scope of medicinally important phenylethylamine derivatives, we tested different halogenated substrates. These were found to be well tolerated giving access to phenylethylamines with potentially improved pharmacological properties (2b)^63^ and precursors (3–5b) for further derivatizations using cross-coupling methods.^64^ By-products stemming from reductive decyanation^65^ were never observed, implying a clean proton reduction followed by hydrogenation of the substrates with the formed M–H species (voltammograms in Fig. S8†). Importantly, the excess charge^66^ does not trigger any side reactions apart from clean hydrogen evolution on the cathode and oxygen on the anode. A substrate with an additional methyl group in α to nitrile positions gave a lower yield of product 6b than the model substrate (20% for 6b*vs.* 88% for 1b), indicating the sensitivity of the hydrogenation towards steric hindrance. The same trend was additionally confirmed by the descending yields of differently bulky products in the series 7–9b. The observed lower yields are a consequence of sluggish reactivity, and the overall mass balance is typically conserved.

Aromatic nitriles were also found to be suitable substrates providing the desired hydrogenated products in comparable yields as aliphatic nitriles. A simple unsubstituted product 10b was obtained in 80% yield. A series of products with different methyl substitutions (11–13b) gave yields, which are consistent with the previous observation of steric hindrance's influence reflected in a marginally lower yield of *ortho*-substituted product 11b. A product with an electron-donating methoxy group 14b showed a slightly lower yield (61%), whereas a combination of steric and electronic effects may suppress the chemical transformation completely (15b). Interestingly, despite similarities in conditions with deprotection protocols for Bn protected amines,^67^ we never observed any noticeable products of C–N bond cleavage, presumably due to relatively mild reaction conditions. Likewise, the dehalogenation reaction was not a major side reaction and gave high yields of chloro (16–18b) and bromo (13b) products.

Importantly, in most examples the crude product is technically pure without any further purification step apart from extraction. Higher purity is achieved by precipitation of the corresponding amine as a hydrochloride salt using HCl in diethyl ether.

### Transfer to flow electrolysis

**Fig. 1:**
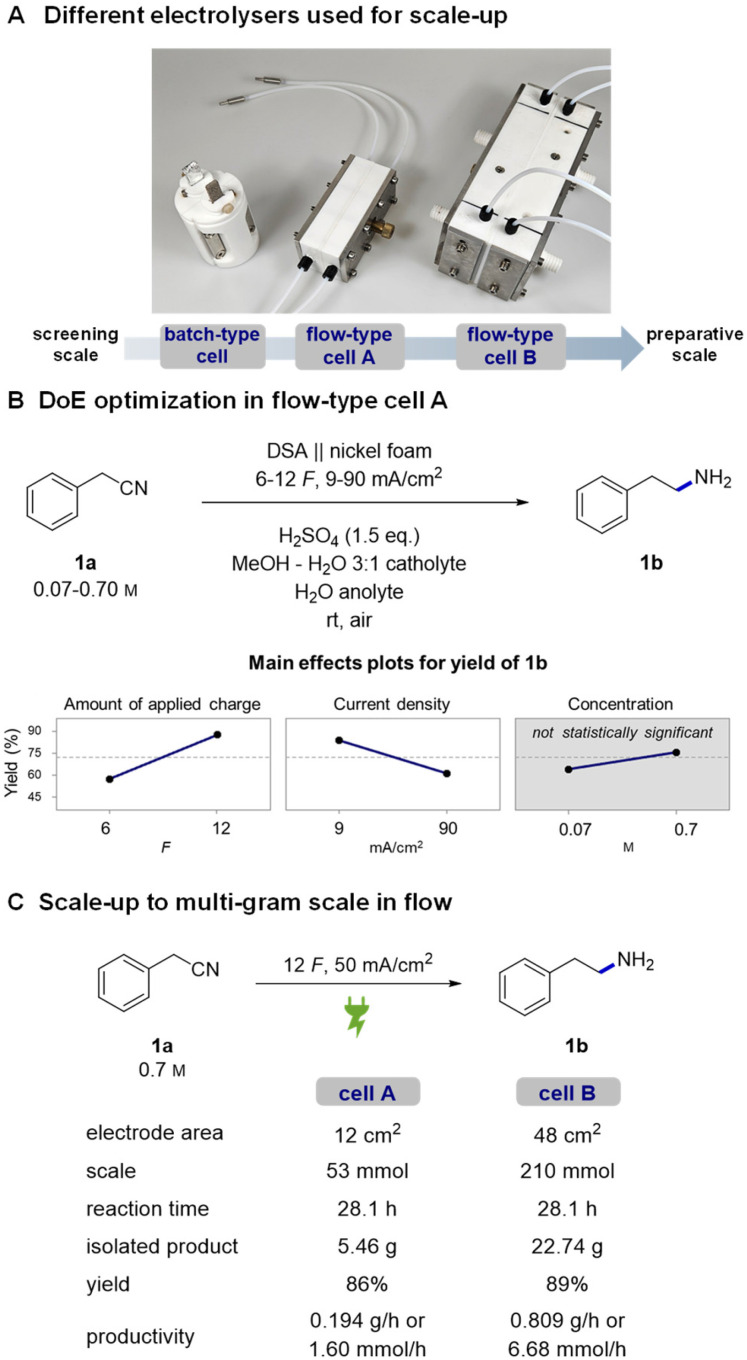
Different electrolysers used for scale-up of the hydrogenation (A) along with reaction trends obtained by full factorial 2^3^ DoE optimization (B). Conditions for the preparative multi-gram scale experiments in flow-type cells A and B (C).

To make the observed reactivity more convenient for large-scale applications, we switched from using batch-type H cells to flow electrolysers (Fig. 1A, see the ESI† for technical data).^49,68–70^ An advantage of flow electrolysers is better tunability of the interelectrode gap – a narrower gap between the electrodes effectively lowers ohmic resistance of the cell and consequently decreases the terminal voltage required for its operation. Additionally, heat dissipation and mass transfer are significantly better than batch-type cells.^71^ Altogether, this makes flow technology a more technically viable approach for scale-up as it offers better reproducibility and lower operational costs.

First, the reaction was tried in a single pass flow mode but due to repeated problems with significant black staining of nickel foam all further attempts in this direction were discontinued. Experiments in recirculating flow mode in contrast worked flawlessly when using a relatively high flow rate of 5 mL min^−1^. By using a flow cell equipped with 12 cm^2^ electrodes,^72^ we achieved good reproducibility and high yields without any noticeable new by-products. Expectedly, a significant 3-fold decrease in the required terminal cell voltage and thereby consumed energy was observed upon decreasing the interelectrode gap from 21 mm in batch-type cells to 1.3 mm in the flow cell (11 V *vs.* 3–4 V at 50 mA cm^−2^, respectively).

To visualize the influence of different reaction parameters and identify any possible cross-interactions, we decided to run a full factorial 2^3^ DoE with preselected continuous parameters (Fig. 1B).^73^ The three parameters – reaction concentration, amount of applied charge and current density – were selected based on our previous experience in batch-type cells. In the case of concentration, the higher value represents a solubility limit of 1a in the reaction solvent. The amount of applied charge and current density were selected to cover a reasonable range around our previously used optimal conditions. Statistical processing of the obtained experimental data revealed the amount of applied charge as the most significant reaction parameter, followed by current density. The reaction concentration was found to be statistically insignificant for the mathematical model. No cross-interactions between the selected parameters were observed (Fig. S6†). Based on the obtained linear trends, we decided to use the highest amount of charge, 12 *F*, and the highest concentration as they ensure better conductivity and thereby lower cell voltage. Using these parameters, current density was screened in a linear fashion with the aim of decreasing the reaction time as much as possible while still achieving full conversion of the nitrile substrate 1a into 1b (Table S6†). A current density of 50 mA cm^−2^ was found to be the highest we can use, which is, interestingly, the same current density as we used in batch-type screening cells as well.

Then, the reaction was tested in a 4-times larger flow cell, having a geometric electrode area of 48 cm^2^. Similarly, a linear current density screening was repeated, and consistently with the smaller cell, 50 mA cm^−2^ current density was found to be optimal (Table S7†). These results clearly demonstrate that the process can be readily transferred from one cell to another without much additional optimization effort.

To demonstrate the practical value of the developed method and to test the stability of the system under prolonged operation, 1-day long multi-gram hydrogenation experiments^74^ were conducted in both flow cells (Fig. 1b). Gratifyingly, in both preparative experiments, cell voltage remained almost constant (3.5–3.8 V) without any major fluctuations, indicating stable and reliable operation of the system. At the end, we managed to isolate 5.46 g (86% yield) of product 1b from the smaller cell experiment and 22.74 g (89% yield) of 1b from the larger cell experiment. With these preparative experiments, we clearly demonstrate that the developed method represents a convenient and safe alternative to classic hydrogenation methods. We also expand the repertoire of gram-scale nickel foam electrochemical applications from homogeneous nickel-catalysed cross-couplings^75–77^ and oxidative ring opening^78,79^ to highly important hydrogenation reactions.

### Reusability of electrodes and surface analysis

**Fig. 2:**
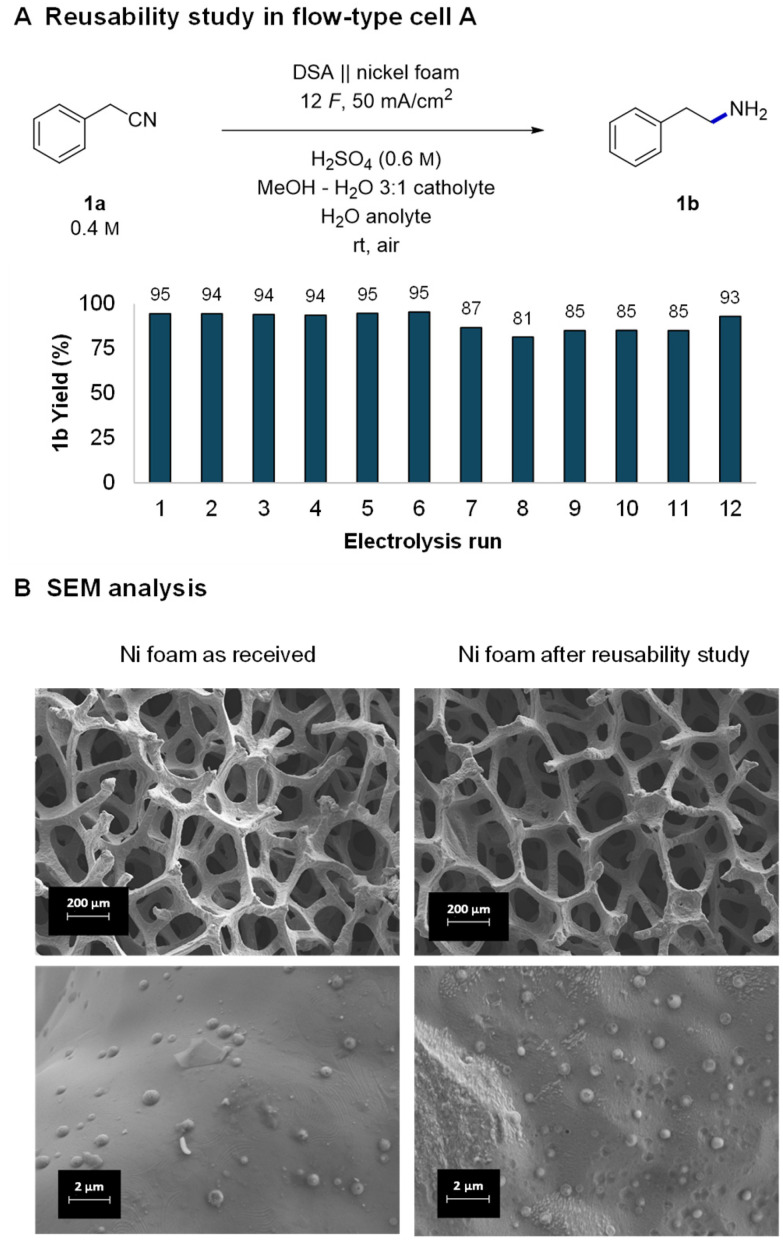
Reusability study of nickel foam (A) and the corresponding scanning electron microscopy (SEM) analysis of the nickel foam's surface (B).

With good indications of the stable performance of the system, we continued with a more systematic investigation of the robustness of the reaction and reusability of the electrodes. The tests were performed by consecutively running reactions in the small flow cell with a washing step of the nickel foam after each run. After 5–6 reactions, the cell was opened and the foam was rinsed with MeOH, dried, and reinstalled in the cell right before the next use. The obtained GC yields of 12 consecutive reactions were all in the 81–95% range (Fig. 2A). In the runs where the yield is lower, the substrate did not get completely consumed. The observed fluctuations in the yields can be attributed either to changes in the activity of the nickel or experimental parameters that are difficult to control, such as a not perfectly parallelly aligned nickel foam electrode or exact flow cell temperature.

Indeed, a detailed investigation of nickel's surface by scanning electron microscopy (SEM) revealed small changes in a piece of foam which was used first in DoE optimization and later in a reusability study (approx. 60 reactions). At low magnifications the surface of the “used nickel foam” did not present significant structural changes (Fig. 2B and Fig. S9, S10†). However, higher magnifications revealed two different types of changes in the surface. The “as-received nickel foam” presented a smooth surface covered by randomly distributed clusters (likely coming from the manufacturing process) whereas some areas of the “used nickel foam” became rougher and others presented the formation of pits on the surface.^40^ However, no buildup of any organic polymeric deposits (fouling) was observed, indicating a relatively clean and robust electrochemical process. The observed changes in the surface may indeed have a minor influence on the activity of the nickel and thus fluctuating yields. Nevertheless, despite the fluctuations, the experiments show that nickel foam can be reused several times without a significant drop in reaction yields. The obtained results are in good agreement with a related complementing study of Martín-Matute's group^20^ in which they studied sustainability of nickel foam in the hydrogenation of olefins under potentiostatic conditions. The durability of other components of the reaction setup, IrO_2_ coated DSA and the Nafion™ membrane is well established (*e.g.*, in the chlor-alkali process)^80^ and was therefore not investigated.

### Sustainability considerations

In addition to convenient scalability, sustainability also represents a notable highlight of this reported electrochemical reaction. The process is sustainable not only due to the good reusability of electrodes, but also due to the low consumption of chemicals, making it almost a waste-free process. The two things that get consumed in the electrochemical reaction are electricity and water.^81^ The water oxidation reaction on DSA leads to oxygen evolution and the release of protons, which are eventually used for hydrogenation on the cathode after passing the cation exchange membrane. Sulfuric acid in this system serves a dual role – as a supporting electrolyte and a source of protons – and importantly, without being consumed in the electrochemical redox events.^42^ The only “real” irrecoverable waste is generated in the workup procedure of the catholyte in the neutralization step of sulfuric acid with sodium hydroxide, which leads to the formation of environmentally benign salt sodium sulfate. In contrast to electrochemical methods,^82^ classical catalytic hydrogenations are not that clean mainly because of polluting hydrogen gas sourcing.^7^ Hydrogen gas is mostly produced by the energy-intensive steam reforming process of natural gas, which emits a considerable amount of carbon monoxide and carbon dioxide, both greenhouse gases. Moreover, transport of hydrogen gas to labs or production plants where it is eventually used in significant excess poses an additional environmental burden. Overall, conventional hydrogen production is a polluting process that needs cleaner alternatives in our environmentally conscious society. Admittedly, the sourcing of an anode in our system which is based on a thin film of iridium oxide represents an environmental burden.^20^ However, this may be circumvented by using carbon-based BDD at a price of higher cell voltage during electrolysis (Table S1†).

Apart from pollution, safety represents another highly relevant aspect of the process's sustainability.^83^ The developed hydrogenation under electrochemical conditions proceeds at ambient temperatures and under an air atmosphere, completely circumventing safety risks related to the handling of pressurized reaction vessels in a classical reaction.^84^ Even though oxygen and hydrogen gases evolve simultaneously during the electrochemical reaction as by-products, they do not pose a serious safety concern as they never come in contact and form explosive gas mixtures. They are generated in separate compartments and can also be easily removed through separated tubes and ideally reused in different processes (*e.g.*, fuel cells).^85^

Overall, it can be concluded that the developed electrochemical process is well in line with the sustainable principles of green chemistry, bringing many advantages in terms of low environmental pollution and inherent process safety (for a detailed evaluation, see ESI chapter 7†).^8,86^

## Conclusions

In summary, we report an operationally very simple electrochemical method for the hydrogenation of nitriles, which is readily scalable and advantageous in terms of sustainability. This method is applicable to non-conjugated aliphatic nitriles and aromatic benzylcyanide derivatives. In contrast to other electrochemical hydrogenation methods, it relies on the use of a practical 2-electrode setup and readily available commercial materials and components. The use of inexpensive nickel foam as the cathode material offers an important advantage in terms of scalability due to its availability and excellent mechanical properties. To capitalize on this, we show the ease of scalability from batch-type screening cells to two differently sized flow cells, eventually reaching a notable productivity of 20 g day^−1^ of phenylethylamine. Reusability studies showed that nickel electrodes can be reused many times without a significant drop in performance. Further investigation of the method's technical relevance for the preparation of different commercially relevant primary amines is in progress.

## Data availability

The data supporting this article have been included as part of the ESI.† All data therein are detailed and will allow an easy reproduction of the results.

## Conflicts of interest

There are no conflicts to declare.

## Supplementary Material

GC-026-D4GC03446E-s001
