# Supplementary material for: Practical electrochemical hydrogenation of nitriles at the nickel foam cathode
Source: Green Chem. 2024 Sep 5;26(20):10567–74. doi: 10.1039/d4gc03446e (PMC11413620; doi:10.1039/d4gc03446e)
Supplement: GC-026-D4GC03446E-s001 [file GC-026-D4GC03446E-s001.pdf]

Supplementary Information (SI) for Green Chemistry.
This journal is © The Royal Society of Chemistry 2024

Supporting information

Practical Electrochemical Hydrogenation of Nitriles at Nickel Foam Cathode

Rok Narobe,[a,b,§] Marcel Nicolas Perner,[a,b,§] María de Jesus Gálvez-Vázquez,[a] Conrad Kuhwald,[c] Martin Klein,[d] Peter Broekmann,[e] Sina Rösler,[c] Bertram Cezanne,[d] Siegfried R. Waldvogel[a,b,*]

[§] Equal contribution

[a]Department of Chemistry, Johannes Gutenberg University, 55128 Mainz, Germany.

[b]Max-Planck-Institute for Chemical Energy Conversion, Stiftstraße 34–36, 45470 Mülheim an der Ruhr, Germany.

[c]Sigma-Aldrich Production GmbH, 9470 Buchs, Switzerland.

[d]Merck Electronics KGaA, 64293 Darmstadt, Germany.

[e]Department of Chemistry, Biochemistry and Pharmaceutical Sciences, University of Bern, 3012 Bern, Switzerland.

Email: siegfried.waldvogel@cec.mpg.de
TEL: +49 208/306-3131

Table of Contents

1 General information S1
2 General experimental procedures S6
2.1 Screening and optimization studies S6
2.2 Preparative experiments S7
3 Screening and optimization tables S9
3.1 Screening and optimization in batch-type cells S9
3.2 Optimization experiments in flow electrolysers S11
3.2.1 Statistical DoE S11
3.2.2 Linear current density screening in 2 cm x 6 cm and 4 cm x 12 cm flow cell S14
4 Limitations of the method S15
5 Cyclic voltammetry S16
6 Reusability study of nickel and SEM images S17
7 Evaluation of Green Chemistry Aspects S19
7.1 Green chemistry calculations for our method S20
7.2 Green chemistry calculations for a comparable reaction S21
8 Characterization data S23
9 NMR spectra S27
10 References S42

1 General information

Starting materials and reagents were purchased from commercial suppliers (Sigma Aldrich, TCI, Alfa Aesar, Acros or Fluka) and were used without further purification. Solvents were used as p.a. grade whereas high purity water was obtained by circulating deionized water through a Milli-Q® water purification system. Reactions were monitored by GC-FID and analytic thin layer chromatography (TLC) using Fluka silica gel plates with a fluorescent indicator. Visualization of the developed TLC chromatogram was performed using 254 nm UV light source. Organic solutions were concentrated using Büchi rotary evaporator.

Power supply

Galvanostat Rohde & Schwarz - HMP4040 was used as a power supply in all the electrochemical reactions. The experiments were performed under galvanostatic conditions using a simple two-electrode reaction setup.

Electrodes

- Nickel foam was purchased from the company Recemat BV, Netherlands. The foam was cut to the desired dimensions with a regular cutter knife.
    - In batch-type screening cells, we used 3.0 mm thick nickel foam “Ni4753” with an average pore size of 0.40 mm.
    - In a small 2 cm x 6 cm flow cell, we used 1.2 mm thick nickel foam “Ni5763” with an average pore size of 0.35 mm. The thinner 1.2 mm foam was used due to lower dead volume which is advantageous for achieving faster homogenization of reaction mixture after addition of internal standard, and it is also beneficial when rinsing the cell with washing solvents after reaction.
    - In a larger 4 cm x 12 cm flow cell, we used 3.0 mm thick nickel foam “Ni4753” with an average pore size of 0.40 mm. In the larger cell, 3 mm thick was used due to better mechanical stability.
- Nickel plate electrode was obtained from IKA Werke GmbH & Co. KG, Germany.
- Iridium oxide coated tantalum dimensionally stable anodes (DSA) were obtained from DeNora, Italy.
- Isostatic graphite Sigrafine™ V2100 was obtained by SGL Carbon, Germany.
- Boron-doped diamond (BDD) with a DIACHEM™ 15 µm diamond layer on silicon were obtained from CONDIAS GmbH, Germany.

Cation exchange membrane

Sulfonic acid-based cation exchange membrane Nafion™ N324 membrane was obtained from DuPont, United States. Prior to use it was conditioned in ca. 5% aq. H_2SO_4. We kept reusing the membranes unless we noticed any visible damage.

NMR spectroscopy

^{1}H NMR and ^{13}C{^{1}H} NMR spectra were recorded at 25 °C on a Bruker AVANCE III HD 500 MHz NMR spectrometer with a Bruker Prodigy probe (Bruker BioSpin GmbH, Rheinstetten, Germany) using D_2O as deuterated solvent. Chemical shifts (δ) are reported in parts per million (ppm). All chemical shifts are reported in δ-scale as parts per million [ppm] (multiplicity, coupling constant J, number of protons), relative to the solvent residual peaks as the internal standard. Coupling constants J are given in Hertz [Hz]. Abbreviations used for signal multiplicity: ^{1}H NMR: s = singlet, d = doublet, t = triplet, q = quartet and m = multiplet.

Ultra performance liquid chromatography – mass spectrometry

Ultra performance liquid chromatography – mass spectrometry (UPLC-MS) was performed on a Waters™ ACQUITY™ UPLC™ H-Class PLUS System (Waters Corporation, Milford, USA) using a quaternary solvent manager (ACQ H-CLASS QSM PLUS), a sample manager with flow-through needle (ACQ H-Class FTN-H PLUS) design, a column heater (ACQUITY UPLC CM-A) and an ACQUITY UPLC® BEH C18 1.7 µm 2.1 x 50 mm column. Mass spectra were measured using a single quadrupole mass detection (ACQUITY QDa Detector) employing ESI+. Acetonitrile (HPLC-MS grade) and water (Milli-Q®) were used as eluents, eluent with 0.1% (v/v) formic acid was added directly before mass detection using a second isocratic solvent manager (Waters™ ACQ Isoc Solvent Mgr).

Gas chromatography

GC measurements were performed on a Shimadzu (Kyoto, Japan) GC-2010 system coupled to an FID. The system was equipped with a capillary column (ZB-5MSi, length 30 m, int. diam. 0.25 mm, film 0.25 µm) and worked with H_2 as carrier gas.

Cyclic voltammetry

Cyclic voltammetry was conducted using a three-electrode setup consisting of a glassy carbon disc working electrode 'WE' (d = 3.0 mm, BASi MF-2012), an Ag/AgCl "leakless" reference electrode 'RE' (EDAQ) and a platinum wire counter-electrode 'CE'. Electrochemical measurements were carried out using an Autolab PGSTAT204 potentiostat at room temperature (ca. 298 K). Before use and between measurements, the working electrode was mechanically cleaned by polishing with diamond suspension (Buehler Metadi Diamond polishing suspension 1 Micron particle size) and repeatedly rinsed with MeOH or distilled water until its surface looked clean and reflective when dry. Supporting electrolyte n-Bu_4NBF_4 (TBAB) was used as supplied commercially from TCI (98%+). Unless otherwise stated, all analytes were prepared as 10 mM solution in 0.1 M TBAB in 3:1 MeOH-H_2O. The solutions were purged with Ar for 15 min prior to measurement. After measuring a few CVs (to see if anything is changing), ferrocene (ca. 2 mg) was added and the solution was purged with Ar for 15 min prior to measuring a new set of CVs. The scanning rate was 50 mV/sec. Data acquisition and processing were performed with Metrohm Autolab Nova 42.1.10.4.1.11.

Scanning electron microscopy

The morphological characterization of the nickel foam was performed using a Zeiss Gemini 450 scanning electron microscope (SEM). An accelerating voltage of 5 kV and a current of 100 pA were applied at a

working distance of 8 mm. The Ni foams were always rinsed in methanol and Milli-Q® water and then dried under ambient conditions before the SEM analysis.

Electrochemical setups

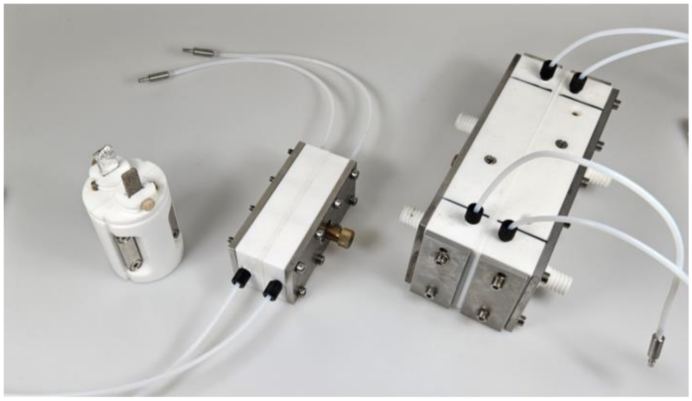

Figure S1: Side-by-side layout of the electrolysers used in this work.

- **Batch-type screening cells**

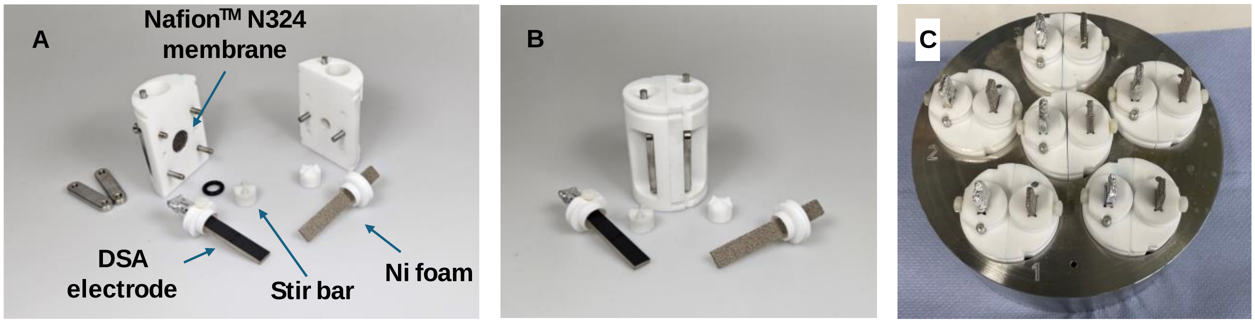

Figure S2: Components of a divided batch-type screening cell (A), assembled screening cell with electrodes (B), 6 screening cells in a screening block (C).

Batch-type screening experiments were conducted in divided Teflon™ electrolysis cells (**Figure S2**) which are commercially available as IKA Screening S8 system package (IKA®-Werke GmbH & Co. KG, Germany). Nafion™ N324 membrane (DuPont, Wilmington, United States) was used as a separator material between the two compartments. The membrane was conditioned in 5% aq. H_2SO_4 for at least 4 hours, washed with fresh H_2O and wiped before being installed in the electrochemical cell. Each cell compartment was equipped with a magnetic stirring bar. Both electrodes, each with exposed geometric surface of 3 cm^2 (submerged part of the electrode in a typical experiment) were arranged at 21 mm distance relative to each other. The reactions were typically performed at room temperature and constant stirring rate 400 rpm. For all the electrochemical experiments in the batch-type screening cells we used Milli-Q® water.

- **Experiments in flow cells**

  - **Small (2 cm x 6 cm) flow-type electrolyser (cell A)**

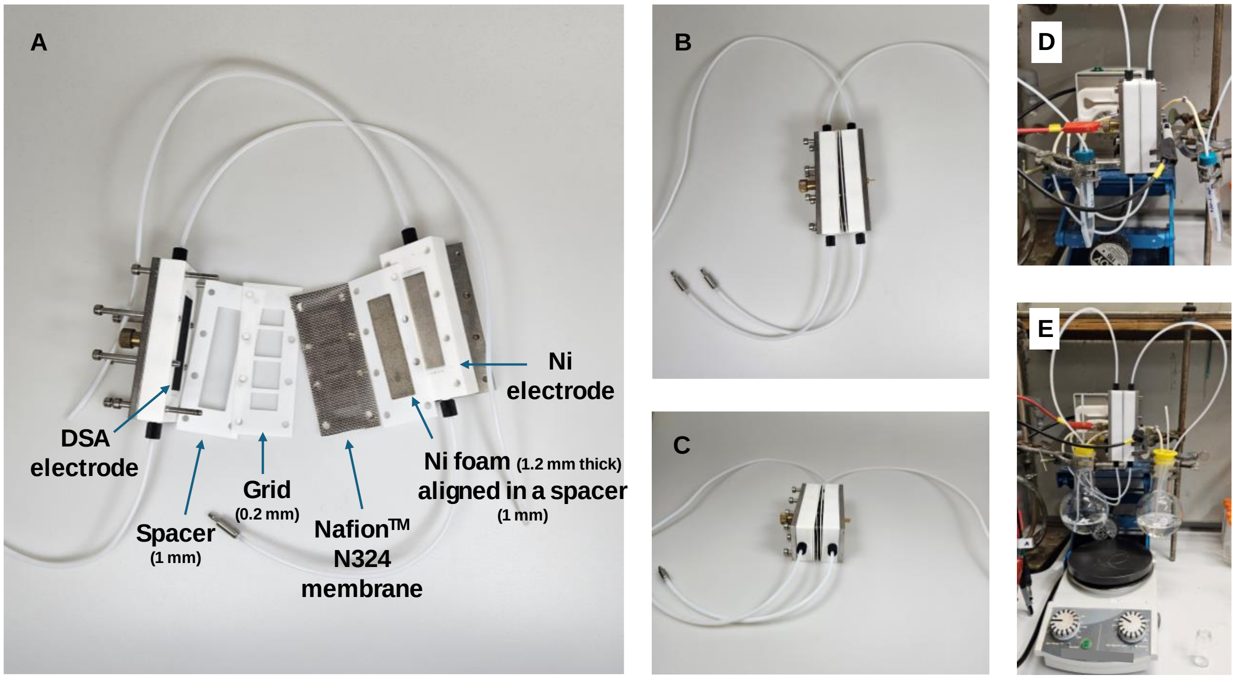

Figure S3: Disassembled flow cell (A), assembled from cell from different perspectives (B and C), flow cell during operation for optimization (D) and scale-up experiments (E).

Experiments in recirculating mode were conducted in a modular flow cell designed by the Waldvogel research group, now commercially available as ElectraSyn flow (IKA®-Werke GmbH & Co. KG, Germany). The cell consists of two Teflon™ half cells, which contain 20 mm x 60 mm electrodes. As a cathode we used nickel plate electrode which is in contact with nickel foam ("Ni 5763", 1.2 mm thick, average pore size 0.35 mm). The foam was cut with a cutter knife to fit tightly into an 18 mm x 67 mm opening in a 1 mm thick Teflon™ spacer. The other half-cell contains 20 mm x 60 mm DSA electrode (IrO_2 on Ta). The two half-cell compartments were separated by Nafion™ N324 cation exchange membrane (DuPont, Wilmington, United States). On the anolyte side of the membrane, Teflon grid was used to prevent contact between IrO_2 DSA electrode and Nafion™ membrane. The gap between DSA electrode and nickel foam is approx. 1.3 mm. Both catholyte and anolyte were pumped through the cell with the same flow rate 5 mL/min using a peristaltic pump with two channels.

- **Large (4 cm x 12 cm) scale flow-type electrolyser (cell B)**

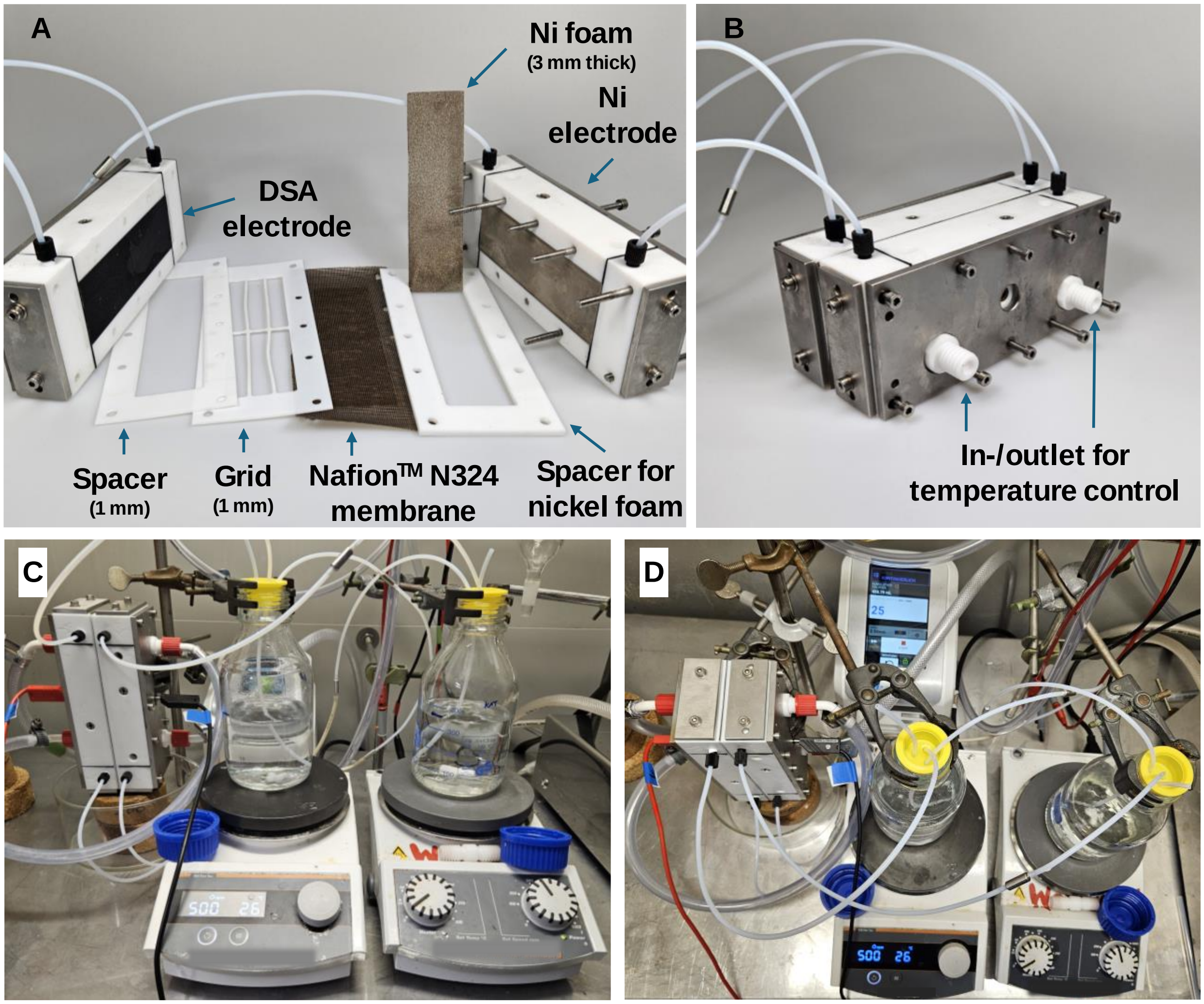

Figure S4: Disassembled flow cell (A), assembled flow cell (B) and setup during operation from two different perspectives (C and D).

Experiments in recirculating mode were conducted in a modular flow cell designed by the Waldvogel research group and built by the workshop at University of Mainz. As a cathode we used 40 mm x 120 mm nickel plate electrode which is in contact with nickel foam ("Ni 4753", 3.0 mm thick, average pore size 0.40 mm). The foam was cut with a cutter knife to fit tightly into a 36 mm x 131 mm opening in a 3 mm thick Teflon™ spacer. The other half-cell contains 40 mm x 120 mm DSA electrode (IrO_2 on Ta). The two half-cell compartments were separated by Nafion™ N324 cation exchange membrane (DuPont, Wilmington, United States). On the anolyte side of the membrane, Teflon grid was used to prevent contact between IrO_2 DSA electrode and Nafion™ membrane. The gap between DSA electrode and nickel foam is approx. 2.1 mm. Both catholyte and anolyte were pumped through the cell with the same flow rate 25 mL/min using a peristaltic pump with two channels.

2 General experimental procedures

2.1 Screening and optimization studies

- General protocol for screening and optimization studies in batch-type cells (GP1)

We used the reaction setup as described and shown in **Figure S2**. The anodic compartment was filled with 7 mL H_2O and the cathodic compartment was filled with 7 mL 3:1 MeOH/H_2O mixture. Then H_2SO_4 (169 µL, 3.15 mmol, 1.5 eq.) was added to both compartments, and in the catholyte additionally benzylcyanide (241 µL, 2.1 mmol, 1 eq.) was added. The electrolysis was started using DSA (IrO_2 on Ta) as anode and nickel foam as cathode. In a standard electrolysis experiment, 2432 C of electric charge were applied under galvanostatic conditions at current density 50 mA/cm^2 (150 mA). After electrolysis, the anolyte and the catholyte were both transferred into a 50 mL centrifuge tube as well as nickel foam electrode and internal standard NPr_3 (210 µL, in general terms 100 µL / 1 mmol of substrate). Both cell compartments were rinsed two more times using ca. 5 mL MeOH per compartment, and the cleaning solvents were collected in the 50 mL centrifuge tube. The centrifuge tube was then closed and shaken for approximately 2 minutes to ensure good homogenization of the reaction mixture "trapped" in a porous structure of nickel foam cathode. For GC-analysis, 100 µL of the so prepared reaction mixture sample was transferred into 1.5 mL conical bottom Eppendorf vial filled up with 1.0 mL 1,4-dioxane and 100 µL of conc. NaOH. The vial was mixed for 2 minutes using vortex mixer. Then, most of the organic phase was transferred into a GC vial and diluted with 1,4-dioxane. The composition of the samples was determined by GC-FID.

- General protocol for screening and DoE optimization studies in 2 cm x 6 cm flow cell (GP2)

We used the reaction setup as described and shown in **Figure S3**. A 15 mL conical bottom centrifuge tube was filled with 6 mL H_2O (anolyte) and another 15 mL centrifuge tube was filled with 6 mL 3:1 MeOH/H_2O mixture (catholyte). Then H_2SO_4 (34-342 µL, 0.6–6.3 mmol, 1.5 eq.) was added to both tubes, and in the catholyte additionally benzylcyanide (48–483 µL, 0.4-4.2 mmol, 1 eq.) was added. The centrifuge tubes were closed, shaken briefly and then connected to the flow cell. In a standard electrolysis experiment, 243–2431 C of electric charge were applied under galvanostatic conditions at current density 9–90 mA/cm^2. After the set amount of electric charge has passed, the anolyte compartment was emptied to prevent diffusion of the product from catholyte into the anolyte. Then, internal standard NPr_3 (42–420 µL; in general terms 100 µL / 1 mmol of substrate) was added, and the reaction mixture was circulated through the reactor for 2 minutes to achieve good homogenization of components. For GC-analysis, 100 µL of the so prepared reaction mixture sample was transferred into 1.5 mL conical bottom Eppendorf vial filled up with 1.0 mL 1,4-dioxane and 100 µL of conc. NaOH. The vial was mixed for 2 minutes using vortex mixer. Then, most of the organic phase was transferred into a GC vial and diluted to a 2 mL mark with 1,4-dioxane. The composition of the samples was determined by GC-FID.

- General protocol for screening and optimization studies in 4 cm x 12 cm flow cell (GP3)

We used the reaction setup as described and shown in **Figure S4**. A 50 mL conical bottom centrifuge tube was filled with 20 mL H_2O (anolyte) and another 50 mL centrifuge tube was filled with 20 mL 3:1 MeOH/H_2O mixture (catholyte). Then H_2SO_4 (1125 µL, 21 mmol, 1.5 eq.) was added to both tubes, and in

the catholyte additionally benzylcyanide (1616 µL, 14 mmol, 1 eq.) was added. The centrifuge tubes were closed, shaken briefly and then connected to the flow cell. In a standard electrolysis experiment, 16209 C of electric charge was applied under galvanostatic conditions at indicated current density (10-60 mA/cm^2). After the set amount of electric charge has passed, the anolyte compartment was emptied to prevent diffusion of the product from catholyte into the anolyte. For GC-analysis, 100 µL of the so prepared reaction mixture sample was transferred into 1.5 mL conical bottom Eppendorf vial filled up with 1.0 mL 1,4-dioxane and 100 µL of conc. NaOH. The vial was mixed for 2 minutes using vortex mixer. Then, most of the organic phase was transferred into a GC vial and diluted to a 2 mL mark with 1,4-dioxane. The composition of the samples was determined by GC-FID.

2.2 Preparative experiments

- General protocol for preparative electrolysis in batch-type divided cell used in synthetic scope investigations on 2.1 mmol scale (GP4)

We used the reaction setup as described and shown in **Figure S2**. The anodic compartment was filled with 7 mL H_2O and the cathodic compartment was filled with 7 mL 3:1 MeOH/H_2O mixture. Then H_2SO_4 (169 µL, 3.15 mmol, 1.5 eq.) was added to both compartments, and in the catholyte additionally nitrile substrate (2.1 mmol) was added.* The electrolysis was started using DSA (IrO_2 on Ta) as anode and nickel foam as cathode. The current density was set to 50 mA/cm^2, and the amount of applied charge was set to 2432 C. The reaction was performed at room temperature and constant stirring rate 400 rpm. After electrolysis, the anolyte and the catholyte were both transferred into a 100 mL round bottom (RB) flask. Both cell compartments were rinsed two more times using ca. 5 mL MeOH per compartment. Also nickel foam electrode was excessively rinsed with MeOH and H_2O to remove any “trapped” reaction mixture from a porous structure of nickel foam cathode. Then, most of MeOH was removed from the collected reaction mixture under reduced pressure by using rotary evaporator. The concentrated reaction mixture was then transferred into a separatory funnel and impurities (e.g., unreacted substrate) were extracted with ca. 40 mL Et_2O. The aqueous phase containing protonated amine was neutralized with NaOH by slowly adding concentrated aq. NaOH while frequently monitoring pH with indicator papers. The addition of base was stopped once the pH reached >13. The basified reaction mixture was extracted three times with 50 mL Et_2O. Combined Et_2O fraction were washed with brine and then dried over Na_2SO_4. The drying agent was filtered, and the amine product was dissolved in ca. 10 mL Et_2O and then precipitated as HCl salt by slow addition of 4 mL of 2 M HCl in Et_2O while stirring. While slowly adding HCl in Et_2O additional Et_2O was added in between to prevent the product from crashing out too fast and blocking the stir bar. To ensure complete precipitation the solution was cooled in an ice bath and kept at room temperature overnight. The hydrochloride salt precipitate was filtered, washed with ca. 20 mL Et_2O and dried on filter paper.

* In cases when the substrate didn’t dissolve well in reaction solvent mixture MeOH/H_2O 3:1, we modified the procedure a bit by first dissolving the substrate in MeOH and then added H_2O.

- Electrolysis in 2 cm x 6 cm flow cell for preparation of 2-phenylethylamine on 52.5 mmol scale

We used the reaction setup as described and shown in **Figure S3**. A 250 mL round bottom flask was filled with 75 mL H_2O (anolyte) and another 250 mL round bottom flask was filled with 75 mL 3:1 MeOH/H_2O mixture (catholyte). Then H_2SO_4 (4217 µL, 78.8 mmol, 1.5 eq.) was added to both RBs while stirring and

cooling, and in the catholyte additionally benzylcyanide (6059 µL, 52.5 mmol, 1 eq.) was added. The RBs were connected to the flow cell and the electrolysis was started. For electrolysis, 60786 C of electric charge were applied under galvanostatic conditions at current density 50 mA/cm^2. After the set amount of electric charge has passed, both compartments were emptied to prevent any diffusion of the product from catholyte into the anolyte. Nickel foam in the cathodic compartment was rinsed by pumping 40 mL of MeOH through the cell. Then, most of MeOH was removed from the collected reaction mixture under reduced pressure by using rotary evaporator. The aqueous phase containing protonated amine was then basified with NaOH. The basification was carried out by slowly adding concentrated aq. NaOH while frequently monitoring pH with indicator papers. The addition of base was stopped once the pH reached >13. The neutralized reaction mixture was transferred into a separatory funnel and extracted two times with 100 mL Et_2O. Combined Et_2O fraction were dried over Na_2SO_4. The drying agent was filtered, and the solvent was evaporated to obtain a slightly yellowish oily product 2-phenylethylamine (5.447 g, 45 mmol, 86%). The purity was confirmed by GC-FID.

- Electrolysis in 4 cm x 12 cm flow cell for preparation of 2-phenylethylamine on 210 mmol scale

We used the reaction setup as described and shown in **Figure S4**. A 500 mL flask was filled with 300 mL H_2O (anolyte) and another 500 mL flask was filled with 300 mL 3:1 MeOH/H_2O mixture (catholyte). Then H_2SO_4 (16.8 mL, 315 mmol, 1.5 eq.) was slowly added to both flasks while stirring and cooling, and in the catholyte additionally benzylcyanide (24.2 mL, 210 mmol, 1 eq.) was added. The flasks were connected to the flow cell and electrolysis was started. For electrolysis, 243142 C of electric charge were applied under galvanostatic conditions at current density 50 mA/cm^2. After the set amount of electric charge has passed, both compartments were emptied to prevent any diffusion of the product from catholyte into the anolyte. Nickel foam in the cathodic compartment was rinsed by pumping 50 mL of MeOH through the cell. Then, most of MeOH was removed from the collected reaction mixture under reduced pressure by using rotary evaporator. The aqueous phase containing protonated amine was then basified with NaOH. The basification was carried out by slowly adding concentrated aq. NaOH while frequently monitoring pH with indicator papers. The addition of base was stopped once the pH reached >13. The neutralized reaction mixture was transferred into a separatory funnel and extracted two times with 200 mL Et_2O. Combined Et_2O fraction were dried over Na_2SO_4. The drying agent was filtered, and the solvent was evaporated to obtain a slightly yellowish oily product 2-phenylethylamine (22.74 g, 187 mmol, 89%). The purity was confirmed by GC-FID.

3 Screening and optimization tables

3.1 Screening and optimization in batch-type cells

Following GP1, influence of different variables on the reaction yield were screened (**Table S1**).

Table S1: General screening of reaction parameters.[a]

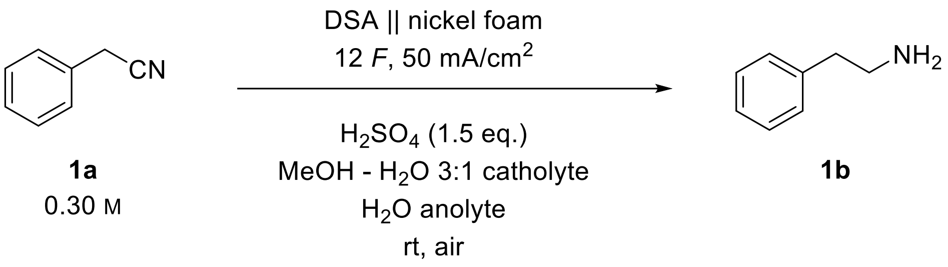

Entry	Deviation from optimized conditions	GC yield **1b** (%)[b]
1	None	91
2	Nickel plate instead of nickel foam	12
3	Nickel plate instead of nickel foam at 5 mA/cm^2	16
4	Less acid (1.5 eq.)	69
5	Graphite as an anode material	96
6	BDD as an anode	95
7	only MeOH in catholyte	78
8	Undivided cell	31
9	1 atm H_2, without electricity	<5

[a]Reaction conditions unless indicated otherwise: anolyte 3.15 mmol H_2SO_4 in 7 mL H_2O, catholyte 2.1 mmol benzylcyanide and 3.15 mmol H_2SO_4 in 7 mL MeOH:H_2O (3:1). Reactions performed using IrO_2 DSA anode and nickel foam cathode, 50 mA/cm^2, 12 *F* in div. cell (Nafion™ N324) at rt under air. Reported current density is calculated based on the geometric area of the electrode. [b]GC yields were determined using tripropylamine as internal standard.

In the next step, the influence of reaction concentration was investigated (**Table S2**). The indicated concentrations in the table refer to the concentration of the substrate. In all the experiments in the series, also concentration of the H_2SO_4 was adjusted so that the ratio between **1a** and H_2SO_4 remained unchanged. At higher concentrations we observed better conductivity.

Table S2: Screening of different reaction concentrations.[a]

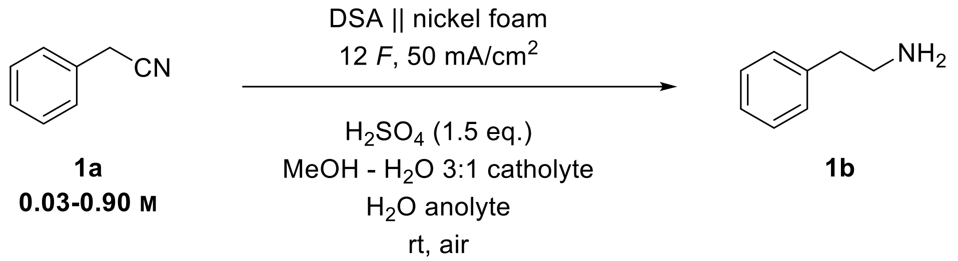

Entry	Concentration of substrate (M)	GC yield **1b** (%)[b]
1	0.03	78
2	0.10	85
**3**	**0.30**	**91**
4	0.90	72

[a]Reaction conditions: anolyte 0.32-9.45 mmol H_2SO_4 in 7 mL H_2O, catholyte 0.21 – 6.30 mmol benzylcyanide and 0.32 – 9.45 mmol H_2SO_4 (1.5 eq.) in 7 mL MeOH:H_2O (3:1). Reactions performed using IrO_2 DSA anode and nickel foam cathode, 50 mA/cm^2, 12 *F* in div. cell (Nafion^{TM} N324) at rt under air. Reported current density is calculated based on the geometric area of the electrode. [b]GC yields were determined using tripropylamine as internal standard.

At 0.3 M reaction concentration highest reaction yield was observed. Then, the Influence of current density was investigated (**Table S3**).

Table S3: Screening of current densities.[a]

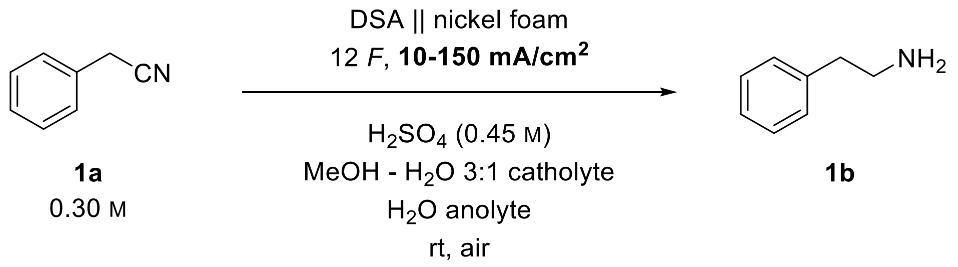

Entry	Current density (mA/cm^2)	GC yield **1b** (%)[b]
1	10	73
2	30	80
**3**	**50**	**91**
4	75	90
5	100	83
6	150	76

[a]Reaction conditions: anolyte 3.15 mmol H_2SO_4 in 7 mL H_2O, catholyte 2.1 mmol benzylcyanide and 3.15 mmol H_2SO_4 in 7 mL MeOH:H_2O (3:1). Reactions performed using IrO_2 DSA anode and nickel foam cathode, 10 – 150 mA/cm^2, 12 *F* in div. cell (Nafion^{TM} N324) at rt under air. Reported current density is calculated based on the geometric area of the electrode. [b]GC yields were determined using tripropylamine as internal standard.

At lower current density, which translate into long reaction times, we observed significant evaporation of reaction solvent through the porous nickel foam cathode (capillary suction and evaporation). At higher current density, we observed slightly elevated reaction temperature.

3.2 Optimization experiments in flow electrolysers

3.2.1 Statistical DoE

The effect of selected continuous reaction parameters on reaction yield was evaluated using the Design of Experiments (DoE) approach (**Table S4**). The experimental design was planned and evaluated with the aid of S14 Minitab 19.2020.1 (Minitab LLC). The limits were selected based on our previous experiences with this system in batch-type screening cells described in subchapter 3.1.

Table S4: Plan for full factorial DoE with 3 parameters.

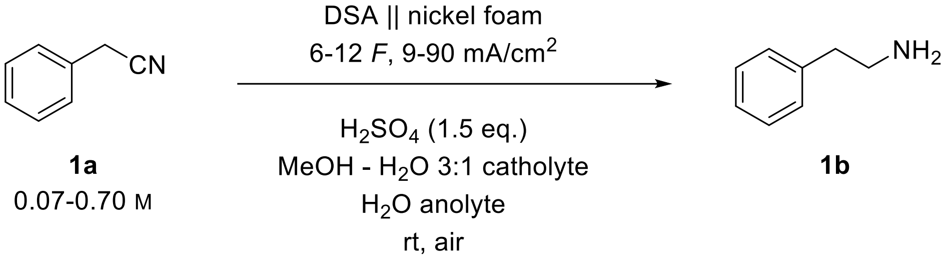

Parameter	Lower limit	Higher limit
Amount of applied charge (*F*)	6	12
Concentration (M)	0.070	0.700
Current density (mA/cm^2)	9	90

The experiments were conducted (**Table S5**) according to general procedure for screening in 2 cm x 6 cm flow cell (GP2). During the study, the cell was disassembled as few times as possible to avoid random errors coming from slightly different alignment of cell, electrodes, membrane, and spacers. After each experiment, both cell compartments were rinsed with approx. 40 mL of H_2O. The water was pumped out of the cell before the next reaction was set. To make the model more reliable, and statistically valid, we repeated each non-central point at least once. In case we observed bigger deviations in yield (more than 7%), we repeated the experiment two more times and crossed out the point which was furthest away from the average value.

Table S5: Performed DoE study experiments and obtained experimental data.[a]

Exp.	Amount of applied Charge (*F*)	Current density (mA/cm^2)	Concentration (M)	GC yields (%)[b]	Avg. GC yield (%)[b]
1	6	90	0.070	37, 38, 34	36
2	12	90	0.070	74, 69, 71	71
3	9	50	0.385	84	84
4	6	90	0.700	47, 41, 50	46
5	9	50	0.385	76	76
6	6	9	0.070	55, 54, 53	54
7	12	9	0.070	94, 96	95
8	12	9	0.700	93, 91	92
9	12	90	0.700	80, 81	81
10	6	9	0.700	79, 86	83
11	9	50	0.385	78	78

[a]The experiments were performed using general procedure 3 (GP3). [b]GC yields were determined using tripropylamine as internal standard. [c]Central points are highlighted in blue

Amount of applied charge and current density were found to have a statistically significant influence on the reaction yield, whereas concentration was found to be statistically insignificant parameter for the model (**Figure S5**). A linear fit model describes the variations in the system reasonably well (R^2= 0.78), without any detected curvatures.

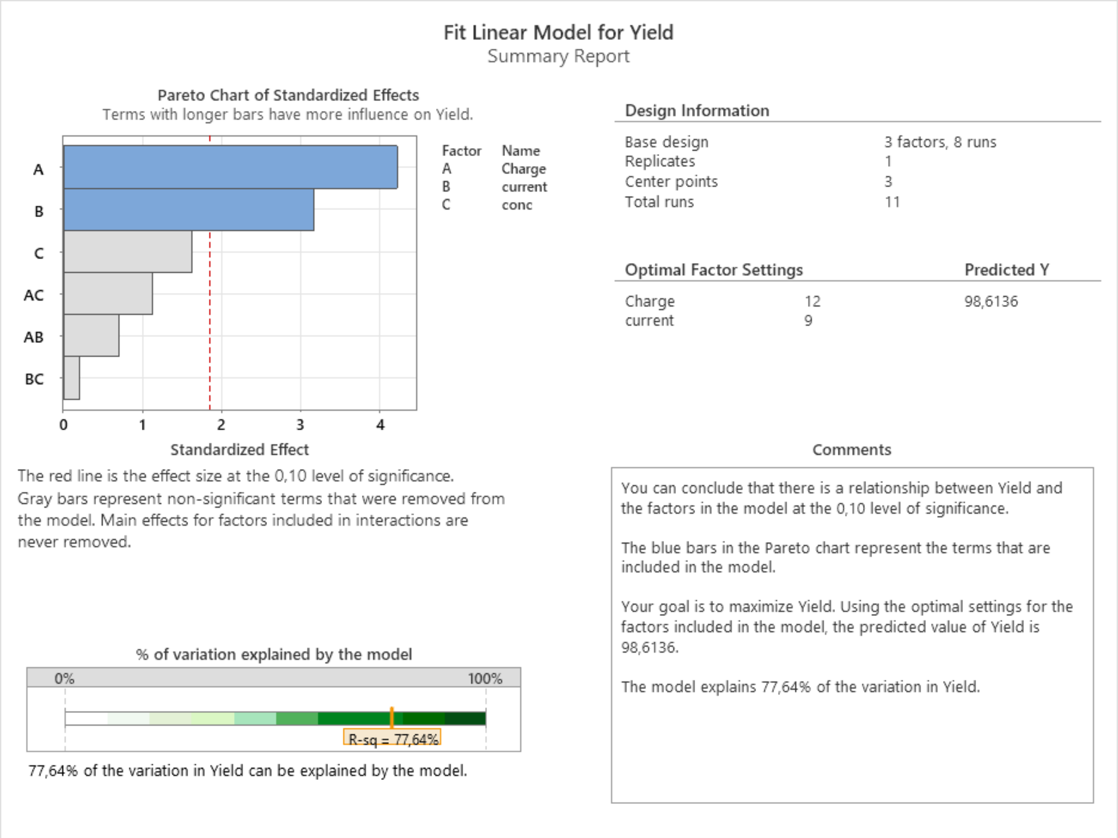

Figure S5: Summary report of the DoE optimization.

No major outliers or suspicious trends were identified among the collected data points (**Figure S6**).

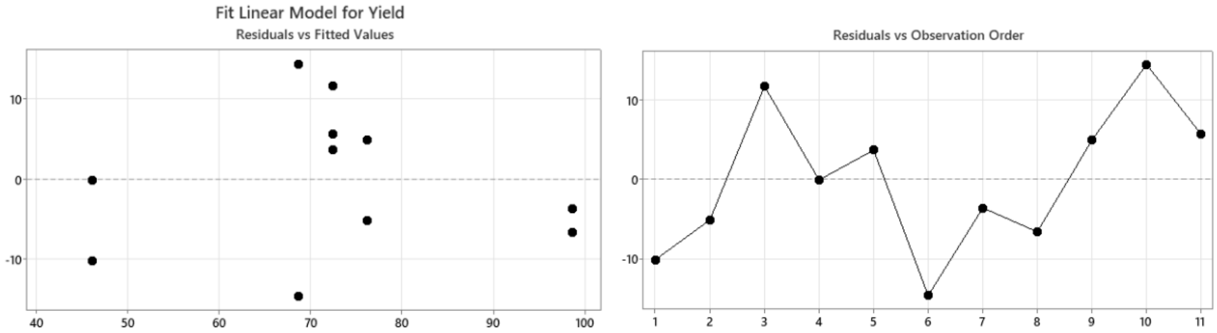

Figure S6: Matching of the experimental points with the model values.

No statistically significant complex interactions between the parameters were detected in the system (**Figure S7**).

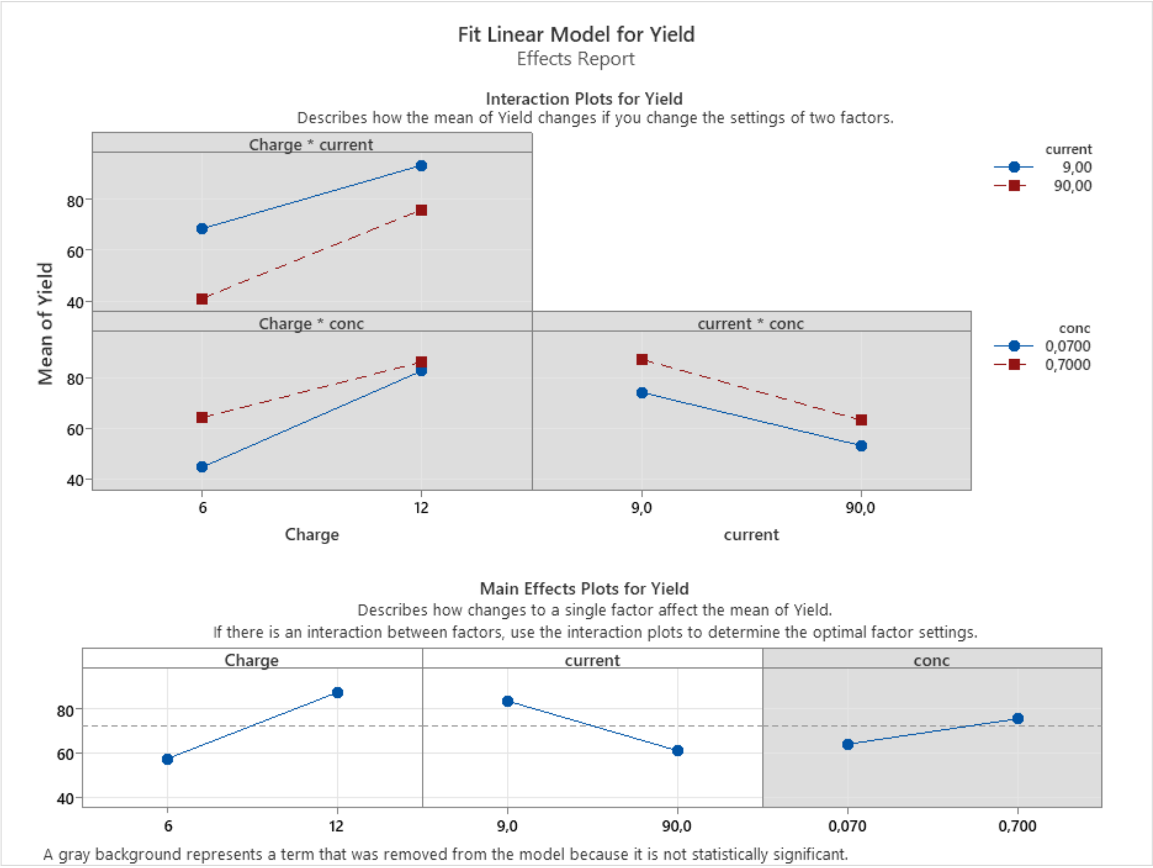

Figure S7: Effect of the parameters on the reaction yield.

3.2.2 Linear current density screening in 2 cm x 6 cm and 4 cm x 12 cm flow cell

Based on the observed linear trends in DoE study, we decided to use the highest amount of charge (12 *F*) and highest concentration (0.7 M) as it ensures better conductivity and thereby lower cell voltage. Due to absence of any complex parameter interactions in the system, we used these parameters and screened current density in a linear fashion. The aim of this screening was to decrease the reaction time as much as possible while still achieving full conversion of nitrile substrate **1a** into **1b** (**Table S6**). The experiments were performed according to a GP2 without adding internal standard to the reaction mixture.

Table S6: Linear current density screening in the 2 cm x 6 cm flow cell.[a]

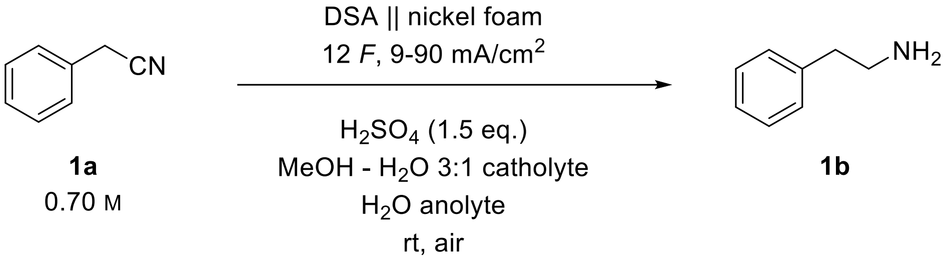

Exp.	Current density (mA/cm^2)	Full conversion of **1a**?
1	20	Yes
2	30	Yes
3	40	Yes
4	50	Yes
5	60	No

[a]Reaction conditions: anolyte 4.2 mmol H_2SO_4 in 6 mL H_2O, catholyte 4.2 mmol benzylcyanide and 6.3 mmol H_2SO_4 in 6 mL MeOH:H_2O (3:1). Reactions performed using IrO_2 DSA anode and nickel foam cathode, 20-60 mA/cm^2, 12 *F* in div. flow cell (Nafion™ N324) at rt under air. Reported current density is calculated based on the geometric area of the electrode.

A current density of 50 mA/cm^2 was found to be highest we can use. Similar experiments were performed also in a larger 4 cm x 12 cm flow cell (**Table S7**) following GP3.

Table S7: Linear current density screening in the 4 cm x 12 cm flow cell.

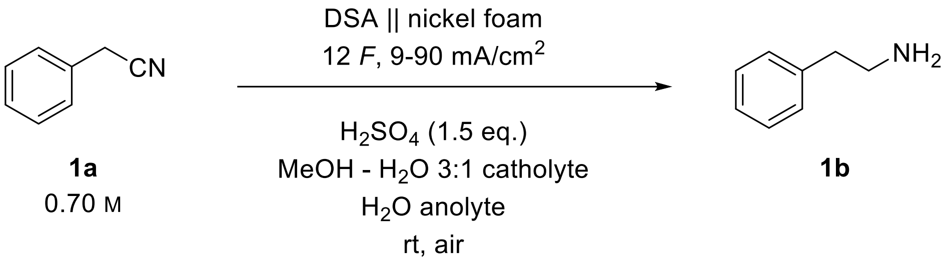

Exp.	Current density (mA/cm^2)	Full conversion of **1a**?
1	30	Yes
2	40	Yes
3	50	Yes
4	55	No

[a]Reaction conditions: anolyte 21 mmol H_2SO_4 in 20 mL H_2O, catholyte 14 mmol benzylcyanide and 21 mmol H_2SO_4 in 20 mL MeOH:H_2O (3:1). Reactions performed using IrO_2 DSA anode and nickel foam cathode, 50 mA/cm^2, 12 *F* in div. cell (Nafion™ N324) at rt under air. Reported current density is calculated based on the geometric area of the electrode.

Interestingly the same current density (50 mA/cm^2) was found to be highest we can use also in the large flow cell.

4 Limitations of the method

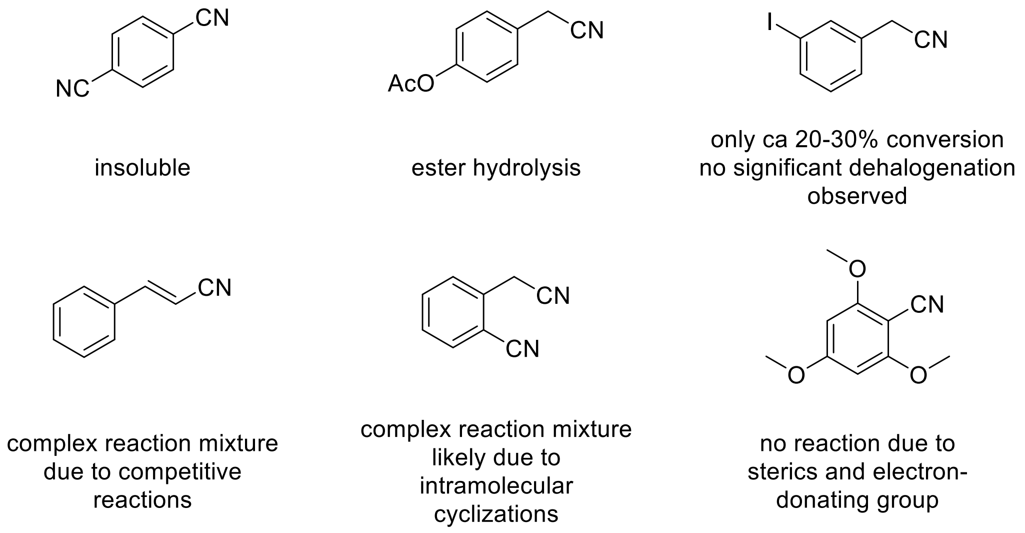

Scheme S1: Representative unsuccessful substrates.

5 Cyclic voltammetry

To elucidate the mechanistic details of the transformation, we performed cyclic voltammetry studies (**Figure S8**).

All analytes were prepared as 10 mM solution in 0.1 M NBu_4BF_4 in 3:1 MeOH-H_2O. The solutions were purged with Ar for 15 min prior to measurement. After measuring a few CVs (to see if anything is changing), internal standard ferrocene (ca. 2 mg) was added and the solution was purged with Ar for 15 min prior to measuring a new set of CVs.

The potentials were measured against the potential of the ferrocene/ferrocenium couple internal reference and were corrected with the conversion constant of +0.37 V to saturated calomel electrode (vs. SCE).

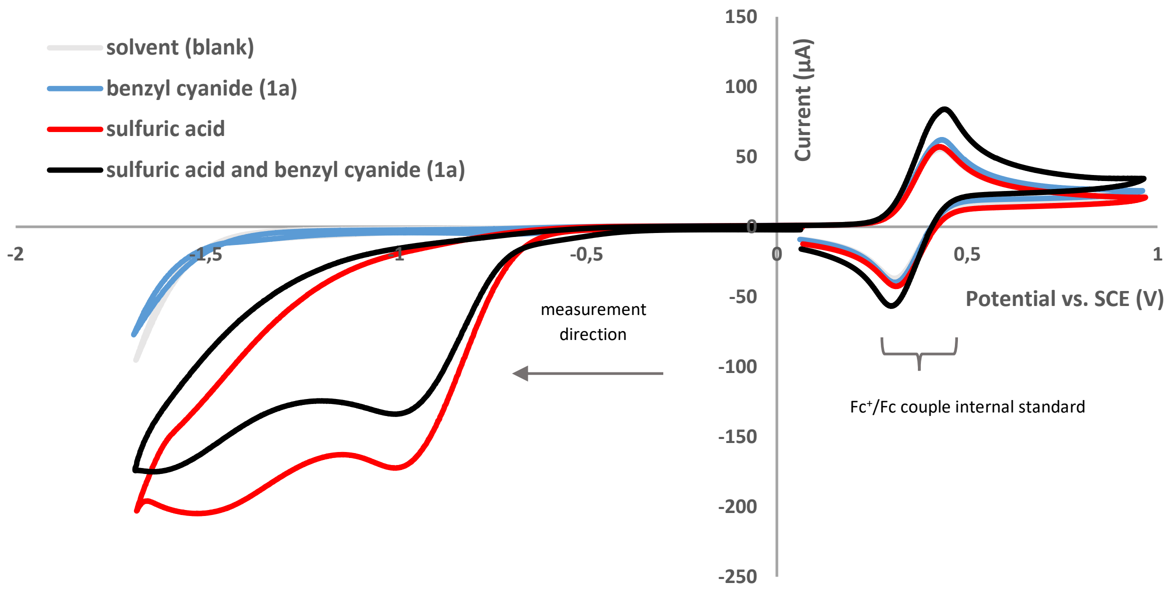

Figure S8: Overlaid voltammograms of benzyl cyanide, sulfuric acid and the combination of both.

The experimental data shows that benzyl cyanide cannot be reduced directly on the electrode (gray and blue line). The only observed significant reduction peak can be assigned to proton reduction (red and black line).

These results imply that formed metal hydride (Ni–H) species are able to efficiently hydrogenate different nitriles irrespective of their electrochemical reduction potential.

6 Reusability study of nickel and SEM images

The experiments were conducted according to general procedure 2 for screening in 2 cm x 6 cm flow cell (GP2). During the study, the cell was disassembled as few times as possible to avoid random errors coming from slightly different alignment of cell, electrodes, membrane, and spacers. After each experiment, both cell compartments were rinsed with approx. 40 mL of H_2O. The water was pumped out of the cell before the next reaction was set.

The piece of nickel foam used in this study was previously used as well for the DoE study.

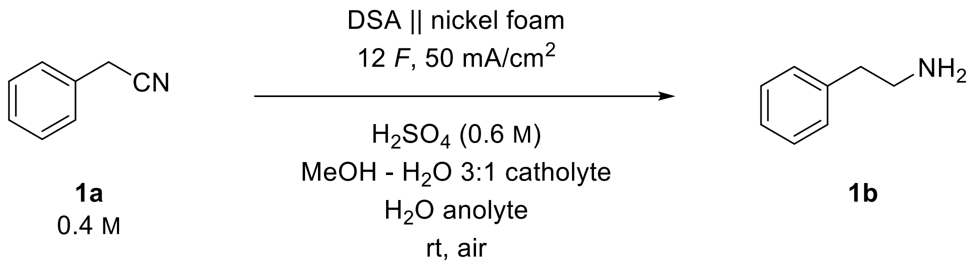

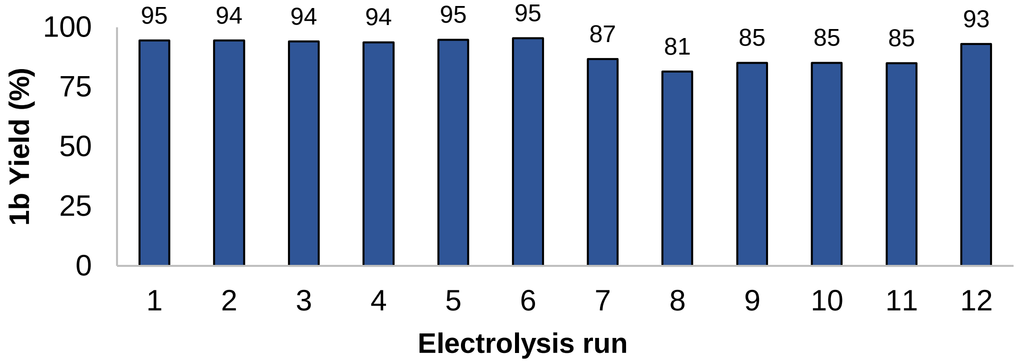

Chart S1: Reusability study of nickel foam.

The Ni foams were always rinsed in methanol and Milli-Q® water and then dried under ambient conditions before the SEM analysis. The porous structure of the nickel foam remained unaffected (**Figure S9** and **Figure S10**). Slight differences in nickel foam surface can be observed only under high magnification (last two photos in the series).

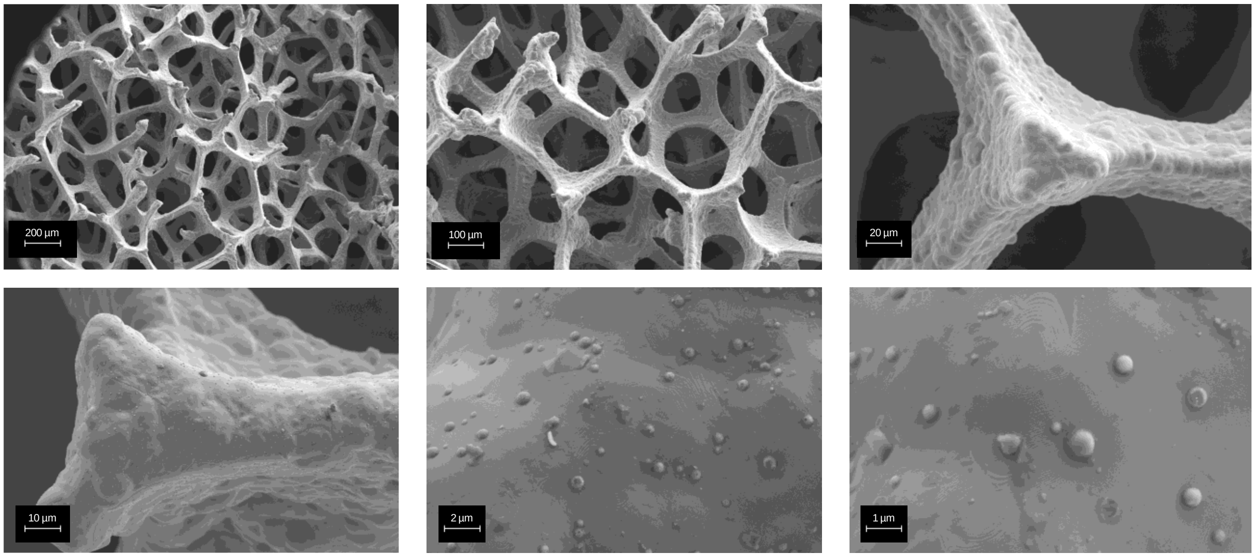

Figure S9: Differently magnified SEM images of nickel foam as received.

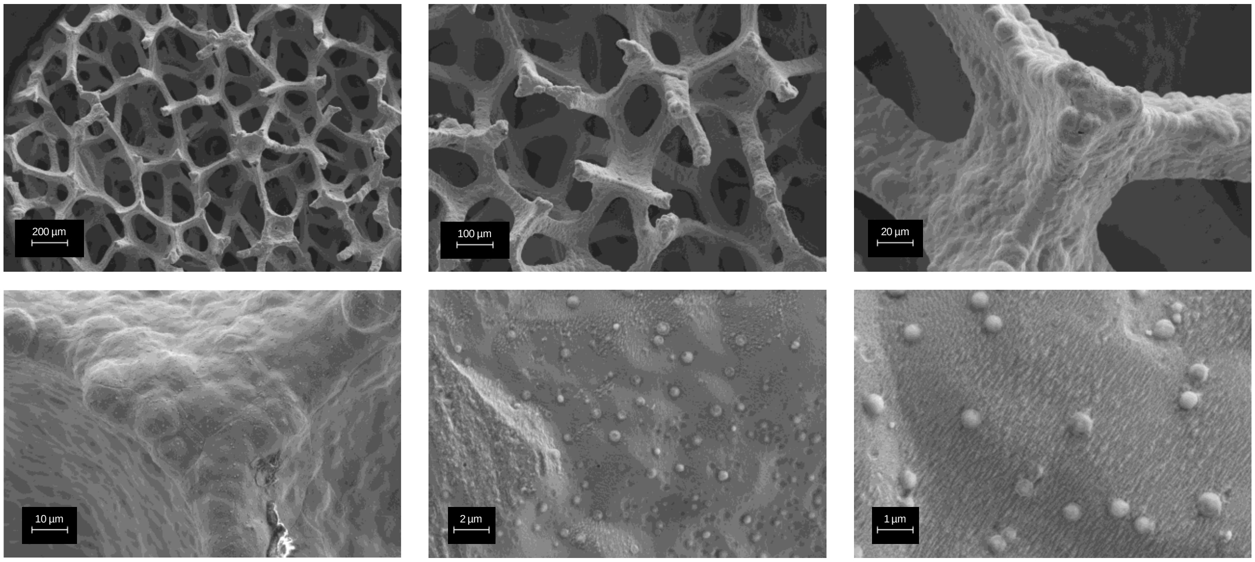

Figure S10: Differently magnified SEM images of nickel foam after DoE study.

7 Evaluation of Green Chemistry Aspects

To evaluate the sustainability of the protocol we developed for the hydrogenation of nitriles, a comparison was made with a conventional, published protocol. To achieve a balanced comparison, six different green chemistry metrics were assessed. This evaluation of sustainability has already been applied by our and other groups and was therefore adapted to our work.[1, 2] A comparison was performed for the model compound benzyl cyanide (**1a**). Our newly developed protocol was compared to a known protocol with high synthetic utility and similar reaction conditions shown in scheme S2.[3]

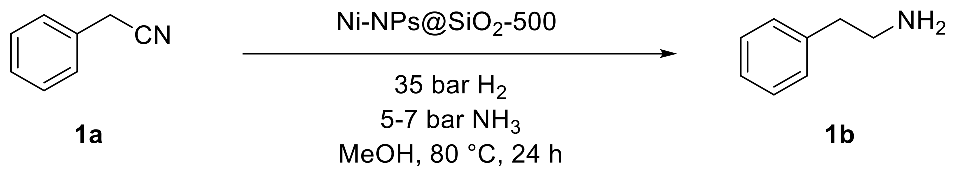

Scheme S2: Published protocol for comparison of sustainability aspects with our developed method.[3]

The authors make use of silica supported nickel-nanoparticles which were prepared by calcination of nickel(II) nitrate on silica and subsequent reduction with hydrogen at elevated temperatures (500 °C). For the reaction, high pressure of hydrogen and ammonia is required in methanol as a solvent at elevated temperatures. For the evaluation of sustainability and the comparison with our work, we excluded the nickel catalyst loading since it can be re-used similar to the nickel foam electrode we use in our method. However, the separation of nickel catalyst from the reaction mixture is an additional step which can be circumvented in our case. Additionally, the calculations do not include the gas pressure of hydrogen and ammonia, as well as the higher temperatures which emphasizes the safety and sustainability aspect of our work.

The cost evaluation covers only consumed and non-recoverable chemicals and refers to the synthesis on a 1 mol scale of the starting material. Prices were taken from the latest Sigma-Aldrich catalog (for the German market). The largest available package size was utilized to determine the price per gram of chemicals. Solvents and acids are calculated on the base of price per 2.5 L.

The economic aspect was calculated using the following formula: [1]

$$Eco = \frac{product\ value\ per\ mol \cdot chemical\ yield\ \%}{reagent\ cost\ per\ mol \cdot 100\%}$$

The atom economy was calculated according to the following formula:[4]

$$AE = \frac{molecular\ mass\ of\ desired\ product}{molecular\ mass\ of\ all\ products} \cdot 100\%$$

The reaction mass efficiency was calculated according to the following formula:[4]

$$RME = \frac{atom\ ecconomy \cdot chemical\ yield}{excess\ reactant\ factor}$$

Effective mass yield was calculated according to the following formula:[5]

$$EMY = \frac{mass\ of\ desired\ product}{mass\ of\ non-benign\ reagents} \cdot 100\%$$

To validate the safety perspective, the protocol was performed according to the GHS classification of the reagents used. The overall GHS classification, which is derived from the average of the GHS classifications, was used for the safety assessment. A classification scale from 1 to 5 was selected, in which 5 stands for very safe and 1 for very unsafe. The assessment was based on the following table and the safety data sheet provided by Sigma-Aldrich. [1, 2]

Table S8: Rating of the reactants.

GHS rating	hazard
1	explosive, oxidizing, toxic, health hazard (or more that 3 hazards)
2	harmful, flammable, environmental, corrosive (combination of 3 hazards)
3	harmful, flammable, environmental, corrosive (combination of 2 hazards)
4	harmful, flammable, environmental, corrosive (1 hazard)
5	None

7.1 Green chemistry calculations for our method

Table S9: Rating of the reactants used in our protocol.

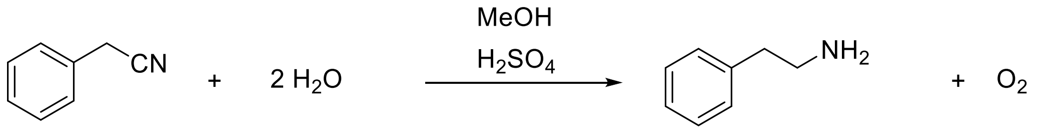

[117.15 g/mol] [18.02 g/mol] [121.18 g/mol] [31.99 g/mol]

**Substance**	***CAS***	***MW / g·mol^-1***	***Price Euro/g***	***GHS Hazard***	***GHS ranking***	***Specification***
Benzylcyanide	140-29-4	117.15	0.05	toxic	1	1 kg, 98%
Methanol	67-56-1	32.04	0.02	flammable, toxic, health hazard	1	≥99.6%
Sulfuric acid	7664-93-9	98.08	0.03	corrosive	4	98%, for analysis EMSURE®
Water	7732-18-5	18.02	0.02	none	5	1 L, deionized for synthesis
2-Phenylethylamine	64-04-0	121.18	0.17	corrosive, toxic	1	250 mL, ≥99.0%, for synthesis

Overall GHS: 2.8

The atom economy factor (AE) was calculated accordingly to the following equation:

$$AE = \frac{121.18\,\frac{g}{mol}}{121.18\,\frac{g}{mol} + 32\,\frac{g}{mol}} \cdot 100\% = 79\%$$

The AE metrics is slightly lowered due to oxygen evolution in the anolyte. However, this can be considered as a very benign by-product.

$$Eco = \frac{20.60\ € \cdot 89\ \%}{5.86\ €\ (Benzylcyanide) + 4.42\ €(H_2SO_4) + 3.95\ €\ (MeOH) \cdot 100\%} = 129$$

The cost of water was set to 0 € in this calculation.

$$RME = \frac{0.79 \cdot 0.89}{46} \cdot 100\% = 1.5\%$$

Water is used as reagent and co-solvent and therefore present in a large excess in the reaction mixture. The calculated value for the reaction mass efficiency (RME) is therefore relatively low. However, as water is harmless and considered as a green reagent/solvent, these metrics can not be considered as representative.

We performed an alternative calculation where water was excluded. We define it as RME*. We believe that this metric describes the system in a more appropriate way.

$$RME^{*} = \frac{0.79 \cdot 0.89}{1} \cdot 100\% = 70.3\%$$

The value for the effective mass yield was calculated as followed:

$$EMY = \frac{121.18 \frac{\mathrm{g}}{\mathrm{mol}}}{117.15 \frac{\mathrm{g}}{\mathrm{mol}}} \cdot 100\% = 103.4\ \%$$

7.2 Green chemistry calculations for a comparable reaction

Table S10: Rating of the reactants used in comparable reaction.[3]

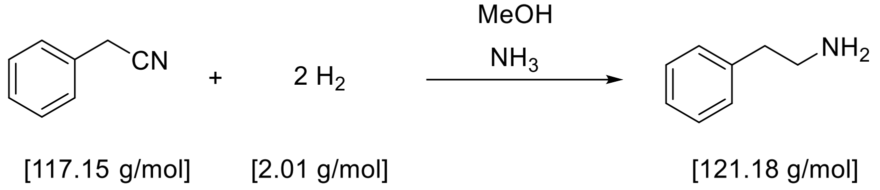

Substance	*CAS*	MW / *g·mol⁻¹*	Price *Euro/g*	*GHS Hazard*	*GHS ranking*	*Specification*
Benzylcyanide	140-29-4	117.15	0.05	toxic	1	1 kg, 98%
Methanol	67-56-1	32.04	0.02	flammable, toxic, health hazard	1	≥99.6%
Hydrogen, gas	1333-74-0	2.01	207.00	flammable, compressed gas	3	Messer® CANgas, 99.999%
Ammonia, gas	7664-41-7	17.03	1.29	compressed gas, toxic,	1	puriss., anhydrous,

				corrosive, nature polluting		≥99.95%, liquefied gas
2-Phenylethylamine	64-04-0	121.18	0.17	corrosive, toxic	1	250 mL, ≥99.0%, for synthesis

Overall GHS: 1.5

$$AE = \frac{121.18\,\frac{\mathrm{g}}{\mathrm{mol}}}{121.18\,\frac{\mathrm{g}}{\mathrm{mol}}} \cdot 100\% = 100\%$$

$$Eco = \frac{20.60\,€ \cdot 90\,\%}{5.86\,€\,(Benzylcyanide) + 832.14\,€\,(H_2) \cdot 100\%} = 2.21$$

$$RME = \frac{1 \cdot 0.9}{17} \cdot 100\% = 5.3\%$$

$$EMY = \frac{121.18\,\frac{\mathrm{g}}{\mathrm{mol}}}{117.15\,\frac{\mathrm{g}}{\mathrm{mol}} + 2.01\,\frac{\mathrm{g}}{\mathrm{mol}}} \cdot 100\% = 101.7\%$$

The difference between the two systems is small because the main contribution in the denominator comes from the molecular mass of the substrate which is toxic. Small difference in the EMYs comes from the use of hydrogen gas under conventional conditions.

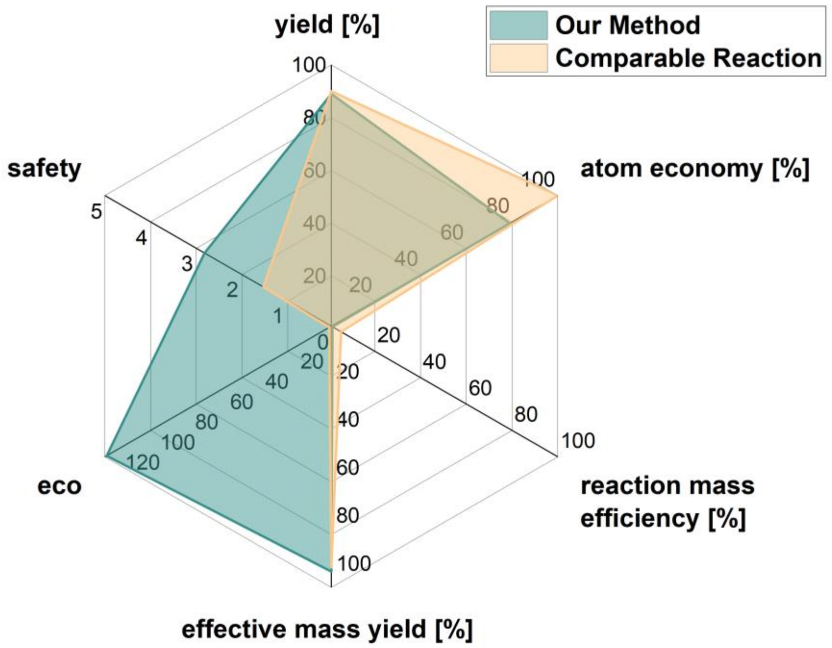

Figure S11: Spider plot comparing the green chemistry aspects of our new method with an already existing method.[3]

8 Characterization data

**2-Phenylethylamine hydrochloride (1b)**	
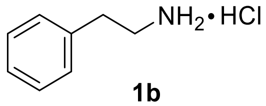 **1b**	
**Yield**	88% (290 mg, 1.84 mmol), white powder
**^{1}H NMR**	(500 MHz, D_2O): δ = 7.45 – 7.38 (m, 2H), 7.38 – 7.28 (m, 3H), 3.27 (t, J = 7.3 Hz, 2H), 2.99 (t, J = 7.3 Hz, 2H) ppm.
**^{13}C NMR**	(126 MHz, D_2O): δ = 136.6, 129.0, 128.8, 127.3, 40.6, 32.7 ppm.
**UPLC-MS (ESI)**	([M+H]$^+$ m/z): calc: 122.10, found: 121.97, “M” refers to freebase amine

The analytical data are in accordance with the literature.[6]

**2-(4-Trifluoromethylphenyl)ethylamine hydrochloride (2b)**	
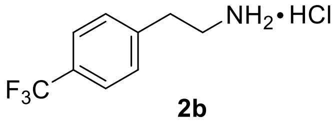 **2b**	
**Yield**	82% (391 mg, 1.73 mmol), white powder
**^{1}H NMR**	(500 MHz, D_2O): δ = 7.73 – 7.68 (m, 2H), 7.51 – 7.45 (m, 2H), 3.31 (t, J = 7.4 Hz, 2H), 3.08 (t, J = 7.4 Hz, 2H) ppm.
**^{13}C NMR**	(126 MHz, D_2O): δ = 140.9, 129.3, 128.7 (q, J = 32.1 Hz), 125.7 (q, J = 3.8 Hz), 124.2 (q, J = 271.2 Hz), 40.2, 32.6 ppm.
**UPLC-MS (ESI)**	([M+H]$^+$ m/z): calc: 190.08, found: 190.02, “M” refers to freebase amine

The analytical data are in accordance with the literature.[7]

**2-(2-Chlorophenyl)ethylamine hydrochloride (3b)**	
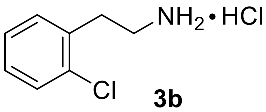 **3b**	
**Yield**	77% (310 mg, 1.61 mmol), white powder
**^{1}H NMR**	(500 MHz, D_2O): δ = 7.51 – 7.44 (m, 1H), 7.41 – 7.24 (m, 3H), 3.28 (t, J = 7.4 Hz, 2H), 3.13 (t, J = 7.4 Hz, 2H) ppm.
**^{13}C NMR**	(126 MHz, D_2O): δ = 134.0, 133.6, 131.2, 129.7, 129.1, 127.5, 39.0, 30.7 ppm.
**UPLC-MS (ESI)**	([M+H]$^+$ m/z): calc: 156.06, found: 155.98, “M” refers to freebase amine

2-(3-Chlorophenyl)ethylamine hydrochloride (4b)	
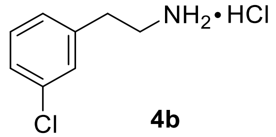 4b	
**Yield**	81% (321 mg, 1.67 mmol), white powder
**^{1}H NMR**	(500 MHz, D_2O): δ = 7.40 – 7.31 (m, 3H), 7.28 – 7.21 (m, 1H), 3.27 (t, *J* = 7.3 Hz, 2H), 2.98 (t, *J* = 7.3 Hz, 2H) ppm.
**^{13}C NMR**	(126 MHz, D_2O): δ = 138.6, 133.9, 130.4, 128.7, 127.3 (d, *J* = 6.5 Hz), 40.3, 32.4 ppm.
**UPLC-MS (ESI)**	([M+H]$^{+}$ m/z): calc: 156.06, found: 155.99, “M” refers to freebase amine

The analytical data are in accordance with the literature.[8]

2-(4-Chlorophenyl)ethylamine hydrochloride (5b)	
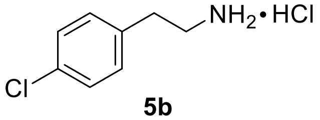 5b	
**Yield**	85% (343 mg, 1.79 mmol), white powder
**^{1}H NMR**	(500 MHz, D_2O): δ = 7.43 – 7.37 (m, 2H), 7.32 – 7.26 (m, 2H), 3.26 (t, *J* = 7.3 Hz, 2H), 2.98 (t, *J* = 7.3 Hz, 2H) ppm.
**^{13}C NMR**	(126 MHz, D_2O): δ = 135.1, 132.4, 130.3, 128.9, 40.3, 32.0 ppm.
**UPLC-MS (ESI)**	([M+H]$^{+}$ m/z): calc: 156.06, found: 155.99, “M” refers to freebase amine

The analytical data are in accordance with the literature.[9]

2-Phenylpropylamine hydrochloride (6b)	
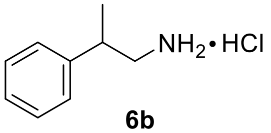 6b	
**Yield**	20% (60 mg, 0.35 mmol), white powder
**^{1}H NMR**	(500 MHz, D_2O): δ = 7.48 – 7.41 (m, 2H), 7.39 – 7.33 (m, 3H), 3.28 – 3.16 (m, 2H), 3.16 – 3.06 (m, 1H), 1.33 (d, *J* = 6.9 Hz, 3H) ppm.
**^{13}C NMR**	(126 MHz, D_2O): δ = 141.8, 129.2, 127.7, 127.3, 45.5, 37.8, 18.6 ppm.
**UPLC-MS (ESI)**	([M+H]$^{+}$ m/z): calc: 136.11, found: 136.02, “M” refers to freebase amine

The analytical data are in accordance with the literature.[10]

Benzylamine hydrochloride (10b)	
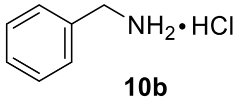 10b	
**Yield**	80% (241 mg, 1.68 mmol), white powder
**^{1}H NMR**	(500 MHz, D_2O): δ = 7.52 – 7.41 (m, 5H), 4.18 (s, 2H) ppm.
**^{13}C NMR**	(126 MHz, D_2O): δ = 132.6, 129.2, 128.8, 43.1 ppm.
**UPLC-MS (ESI)**	([M+H]$^{+}$ m/z): calc: 108.16, found: 108.01, “M” refers to freebase amine

The analytical data are in accordance with the literature.[6]

**2-Methylbenzylamine hydrochloride (11b)**	
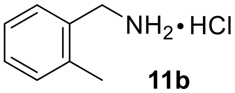 **11b**	
**Yield**	59% (193 mg, 1.23 mmol), white powder
**^{1}H NMR**	(500 MHz, D_2O): δ = 7.40 – 7.27 (m, 4H), 4.22 (s, 2H), 2.37 (s, 3H) ppm.
**^{13}C NMR**	(126 MHz, D_2O): δ = 137.1, 130.9, 130.8, 129.3, 129.0, 126.6, 40.4, 18.0 ppm.
**UPLC-MS (ESI)**	([M+H]$^+$ m/z): calc: 122.10, found: 122.01, “M” refers to freebase amine

The analytical data are in accordance with the literature.[6]

**3-Methylbenzylamine hydrochloride (12b)**	
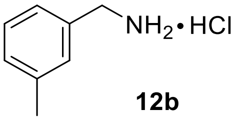 **12b**	
**Yield**	88% (291 mg, 1.85 mmol), white powder
**^{1}H NMR**	(500 MHz, D_2O): δ = 7.40 – 7.34 (m, 1H), 7.32 – 7.21 (m, 3H), 4.13 (s, 2H), 2.35 (s, 3H) ppm.
**^{13}C NMR**	(126 MHz, D_2O): δ = 139.4, 132.6, 129.8, 129.3, 129.1, 125.7, 43.0, 20.3 ppm.
**UPLC-MS (ESI)**	([M+H]$^+$ m/z): calc: 122.10, found: 121.98

The analytical data are in accordance with the literature.[11]

**4-Methylbenzylamine hydrochloride (13b)**	
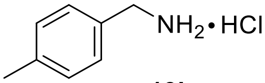 **13b**	
**Yield**	86% (285 mg, 1.80 mmol), white powder
**^{1}H NMR**	(500 MHz, D_2O): δ = 7.37 – 7.33 (m, 2H), 7.32 – 7.27 (m, 2H), 4.13 (s, 2H), 2.34 (s, 3H) ppm.
**^{13}C NMR**	(126 MHz, D_2O): δ = 139.6, 129.7, 129.5, 128.8, 42.8, 20.2 ppm.
**UPLC-MS (ESI)**	([M+H]$^+$ m/z): calc: 122.10, found: 122.11, “M” refers to freebase amine

The analytical data are in accordance with the literature.[6]

**4-Methoxybenzylamine hydrochloride (14b)**	
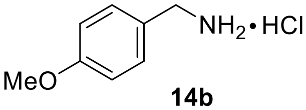 **14b**	
**Yield**	61% (224 mg, 1.29 mmol), white powder
**^{1}H NMR**	(500 MHz, D_2O): δ = 7.44 – 7.37 (m, 2H), 7.07 – 7.01 (m, 2H), 4.12 (s, 2H), 3.84 (s, 3H) ppm.
**^{13}C NMR**	(126 MHz, D_2O): δ = 159.4, 130.5, 125.1, 114.5, 55.3, 42.6 ppm.
**UPLC-MS (ESI)**	([M+H]$^+$ m/z): calc: 138.09, found: 138.13, “M” refers to freebase amine

The analytical data are in accordance with the literature.[6]

**2-Chlorobenzylamine hydrochloride (16b)**	
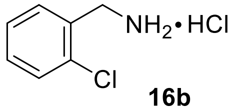 **16b**	
**Yield**	67% (250 mg, 1.40 mmol), white powder
**^{1}H NMR**	(500 MHz, D_2O): δ = 7.56 – 7.48 (m, 2H), 7.46 – 7.36 (m, 2H), 4.31 (s, 2H) ppm.
**^{13}C NMR**	(126 MHz, D_2O): δ = 133.7, 131.2, 131.1, 130.1, 129.9, 127.8, 40.8 ppm.
**UPLC-MS (ESI)**	([M+H]$^+$ m/z): calc: 142.04, found: 141.94, “M” refers to freebase amine

The analytical data are in accordance with the literature.[12]

**3-Chlorobenzylamine hydrochloride (17b)**	
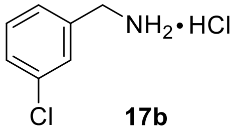 **17b**	
**Yield**	84% (313 mg, 1.75 mmol), white powder
**^{1}H NMR**	(500 MHz, D_2O): δ = 7.49 – 7.40 (m, 3H), 7.40 – 7.34 (m, 1H), 4.17 (s, 2H) ppm.
**^{13}C NMR**	(126 MHz, D_2O): δ = 134.4, 134.1, 130.6, 129.2, 128.7, 127.2, 42.5 ppm.
**UPLC-MS (ESI)**	([M+H]$^+$ m/z): calc: 142.04, found: 141.97, “M” refers to freebase amine

The analytical data are in accordance with the literature.[13]

**4-Chlorobenzylamine hydrochloride (18b)**	
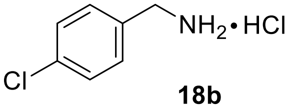 **18b**	
**Yield**	69% (257 mg, 1.44 mmol), white powder
**^{1}H NMR**	(500 MHz, D_2O): δ = 7.50 – 7.46 (m, 2H), 7.45 – 7.38 (m, 2H), 4.16 (s, 2H) ppm.
**^{13}C NMR**	(126 MHz, D_2O): δ = 134.5, 131.2, 130.4, 129.1, 42.4 ppm.
**UPLC-MS (ESI)**	([M+H]$^+$ m/z): calc: 142.04, found: 141.97, “M” refers to freebase amine

The analytical data are in accordance with the literature.[6]

**3-Bromobenzylamine hydrochloride (19b)**	
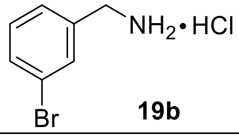 **19b**	
**Yield**	54% (250 mg, 1.13 mmol), white powder
**^{1}H NMR**	(500 MHz, D_2O): δ = 7.66 – 7.60 (m, 2H), 7.46 – 7.33 (m, 2H), 4.16 (s, 2H) ppm.
**^{13}C NMR**	(126 MHz, D_2O): δ = 134.7, 132.2, 131.7, 130.9, 127.6, 122.2, 42.4 ppm.
**UPLC-MS (ESI)**	([M+H]$^+$ m/z): calc: 185.99, found: 185.96, “M” refers to freebase amine

9 NMR spectra

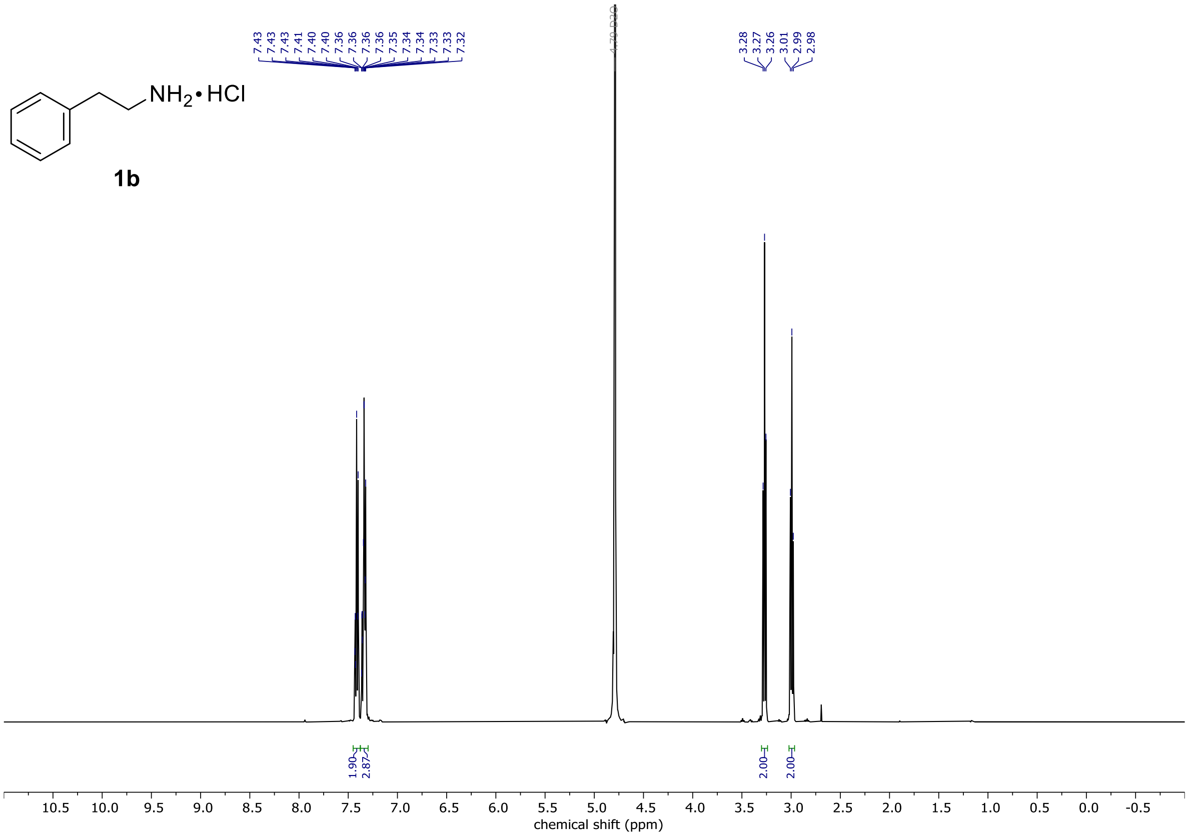

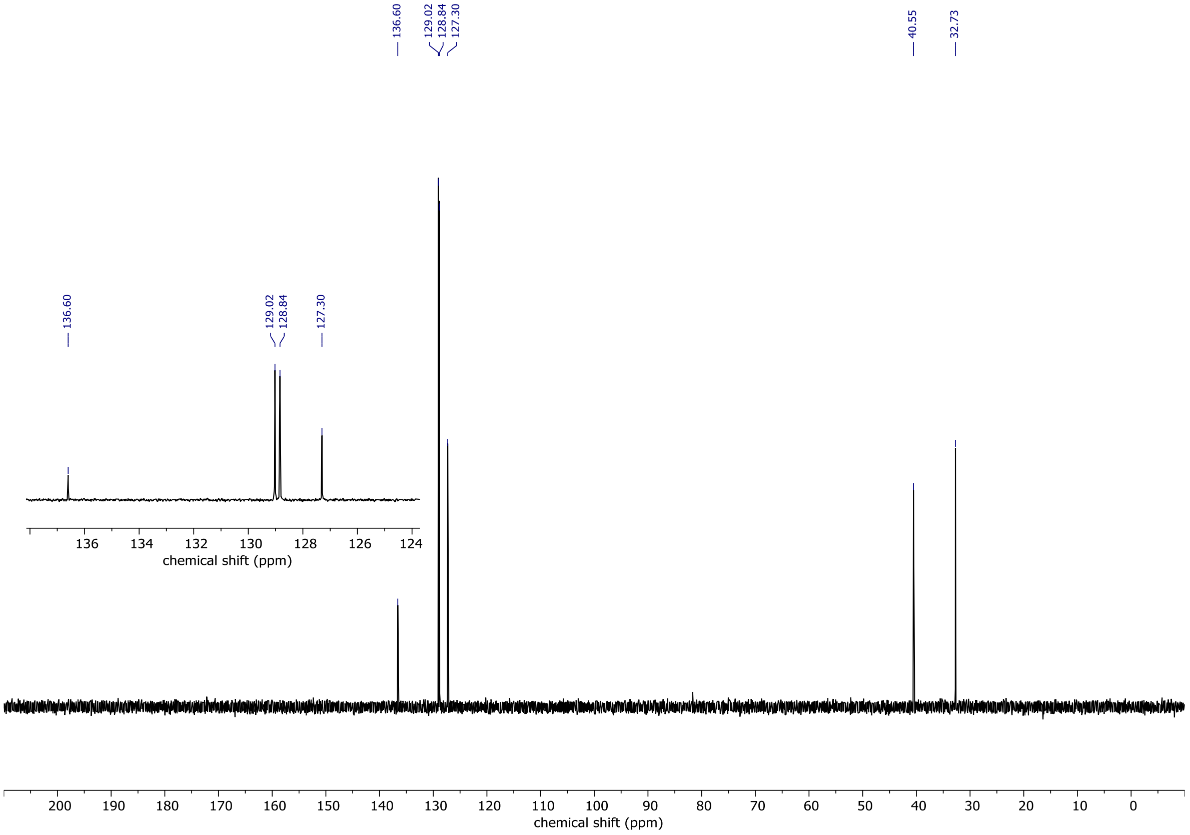

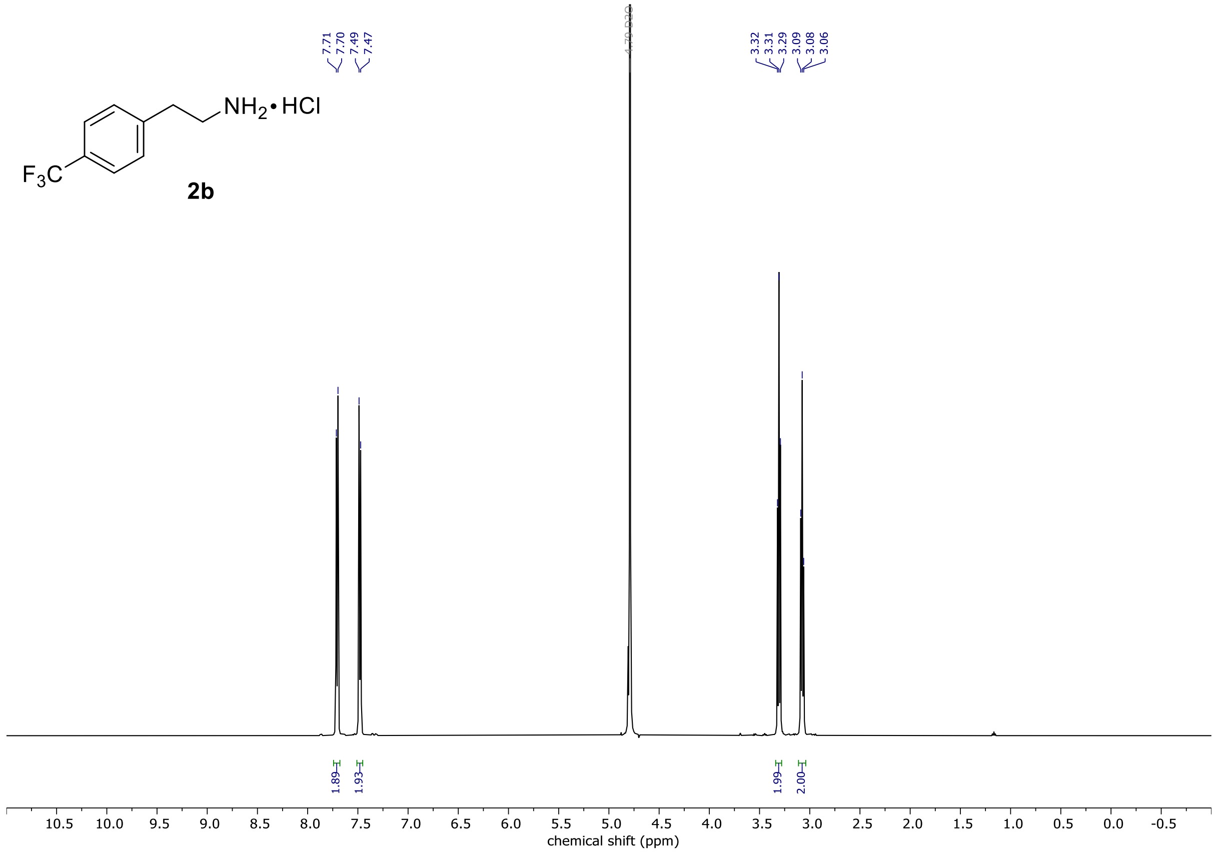
NH2•HCl
F3C
2b
7.71
7.70
7.49
7.47
3.32
3.31
3.29
3.09
3.08
3.06
1.89
1.93
1.99
2.00
10.5 10.0 9.5 9.0 8.5 8.0 7.5 7.0 6.5 6.0 5.5 5.0 4.5 4.0 3.5 3.0 2.5 2.0 1.5 1.0 0.5 0.0 -0.5
chemical shift (ppm)

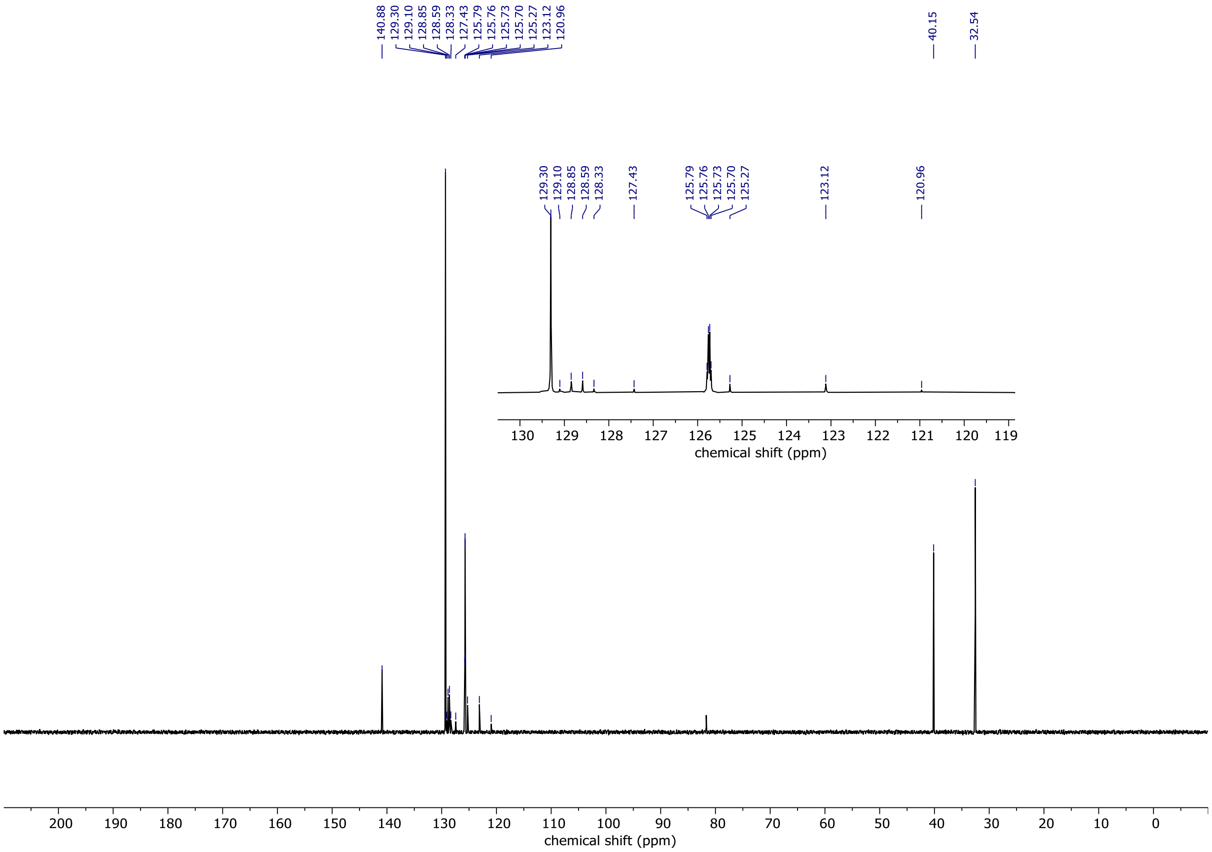
140.88
129.30
129.10
128.85
128.59
128.33
127.43
125.79
125.76
125.73
125.70
125.27
123.12
120.96
40.15
32.54
130 129 128 127 126 125 124 123 122 121 120 119
chemical shift (ppm)
200 190 180 170 160 150 140 130 120 110 100 90 80 70 60 50 40 30 20 10 0
chemical shift (ppm)

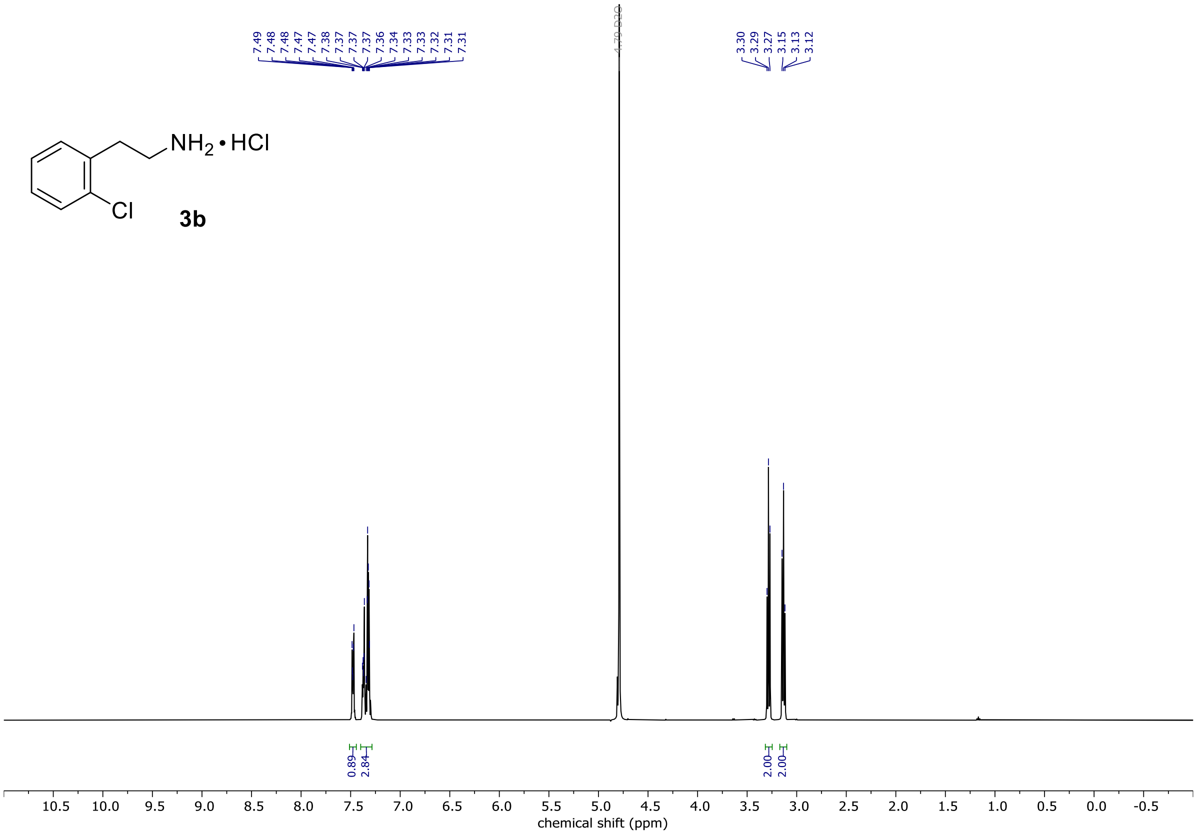

2024e05101.2.fid
SRW-MP-348-15 in D2O 13C{1H}

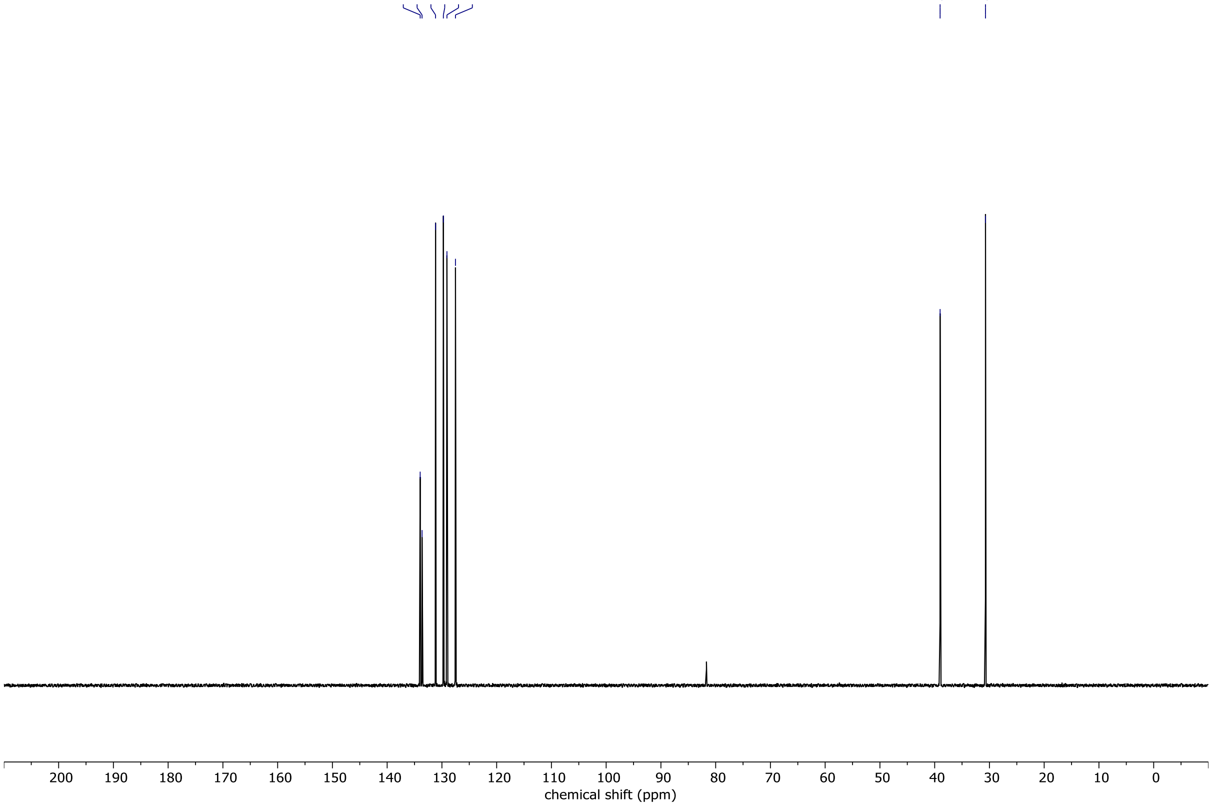

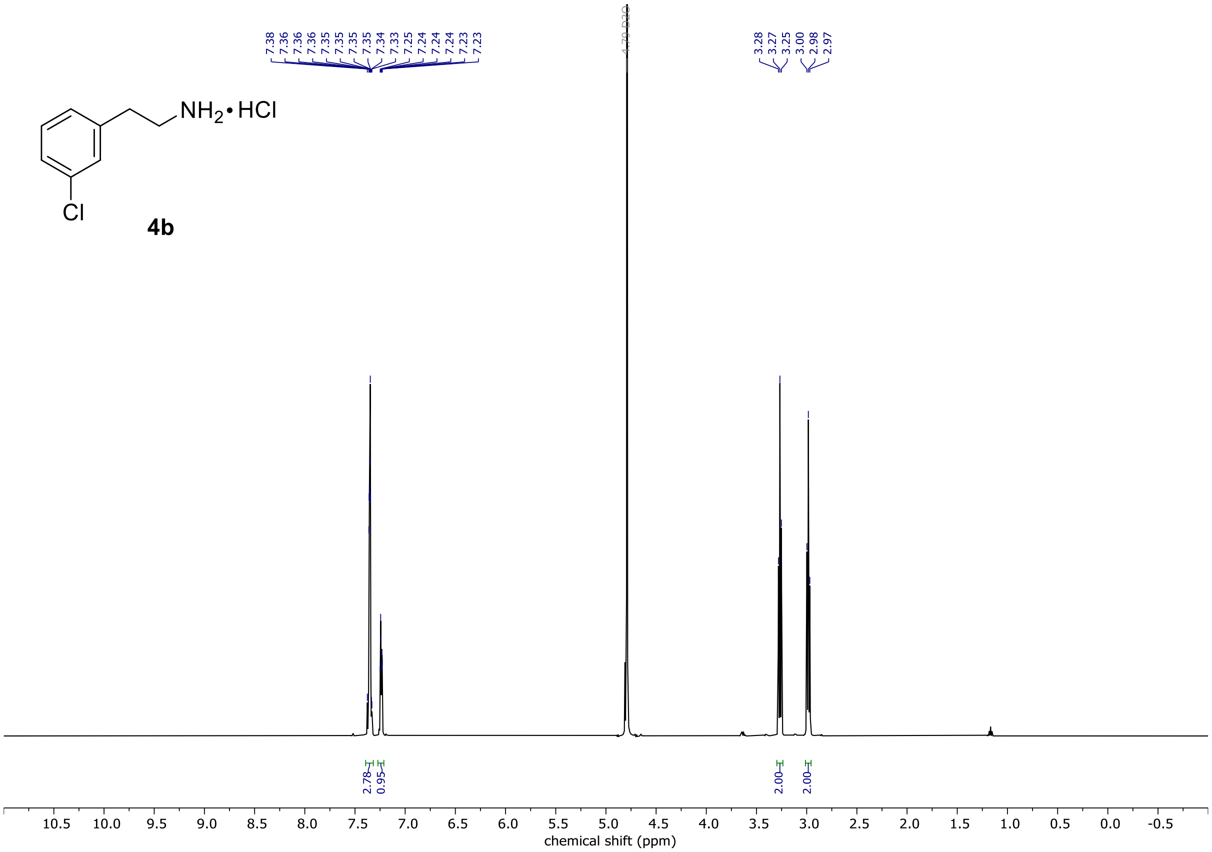
NH2•HCl
Cl
4b
7.38
7.36
7.36
7.36
7.35
7.35
7.35
7.35
7.34
7.33
7.25
7.24
7.24
7.24
7.23
7.23
3.28
3.27
3.25
3.00
2.98
2.97
2.78
0.95
2.00
2.00
10.5 10.0 9.5 9.0 8.5 8.0 7.5 7.0 6.5 6.0 5.5 5.0 4.5 4.0 3.5 3.0 2.5 2.0 1.5 1.0 0.5 0.0 -0.5
chemical shift (ppm)

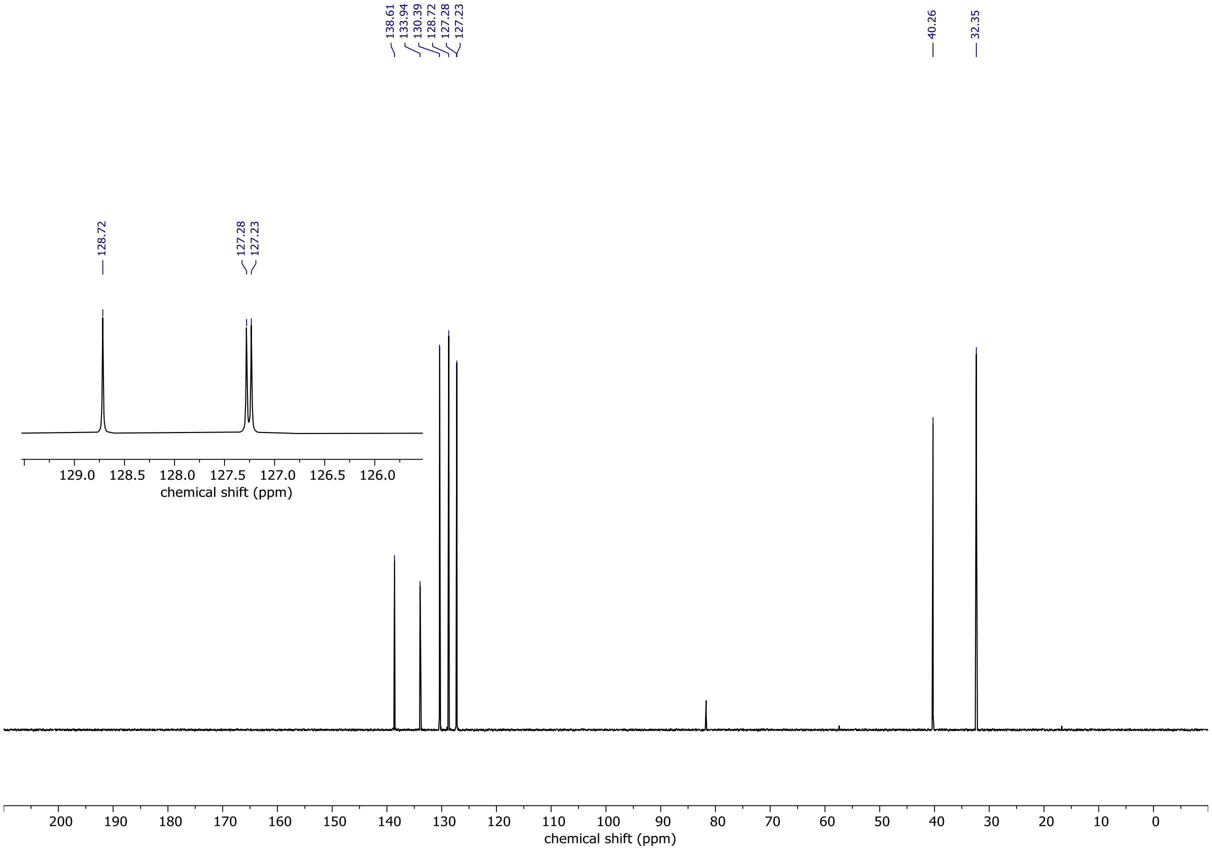
138.61
133.94
130.39
128.72
127.28
127.23
40.26
32.35
128.72
127.28
127.23
129.0 128.5 128.0 127.5 127.0 126.5 126.0
chemical shift (ppm)
200 190 180 170 160 150 140 130 120 110 100 90 80 70 60 50 40 30 20 10 0
chemical shift (ppm)

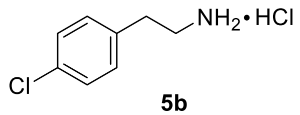

5b

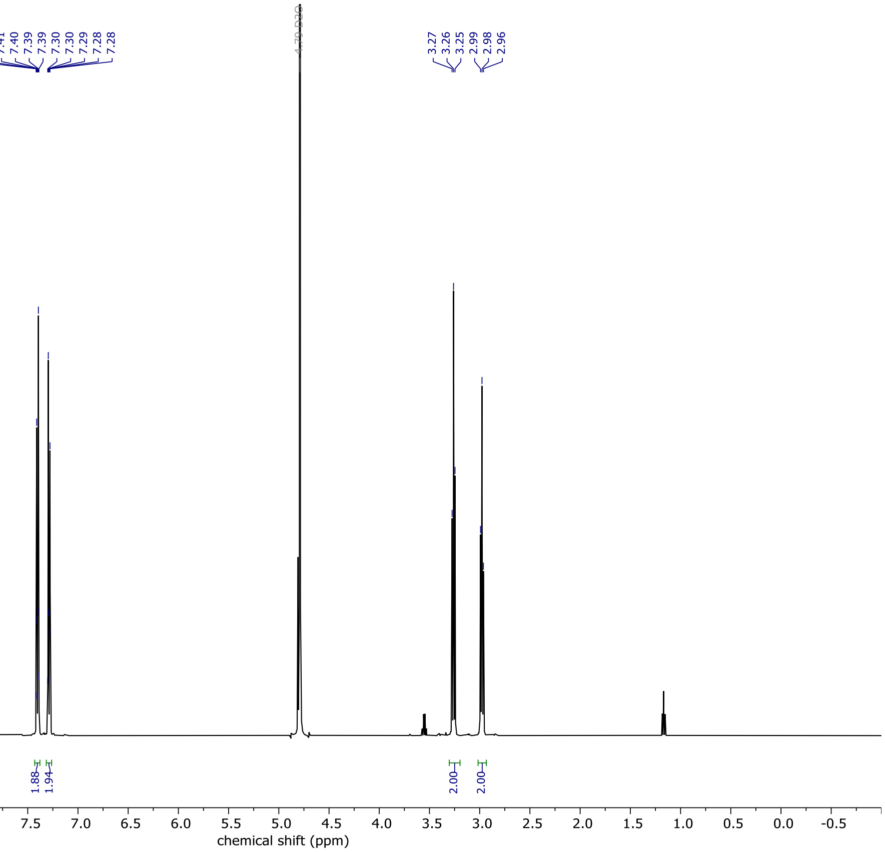

7.42
7.41
7.41
7.40
7.39
7.39
7.30
7.30
7.29
7.28
7.28
3.27
3.26
3.25
2.99
2.98
2.96
1.88
1.94
2.00
2.00
chemical shift (ppm)

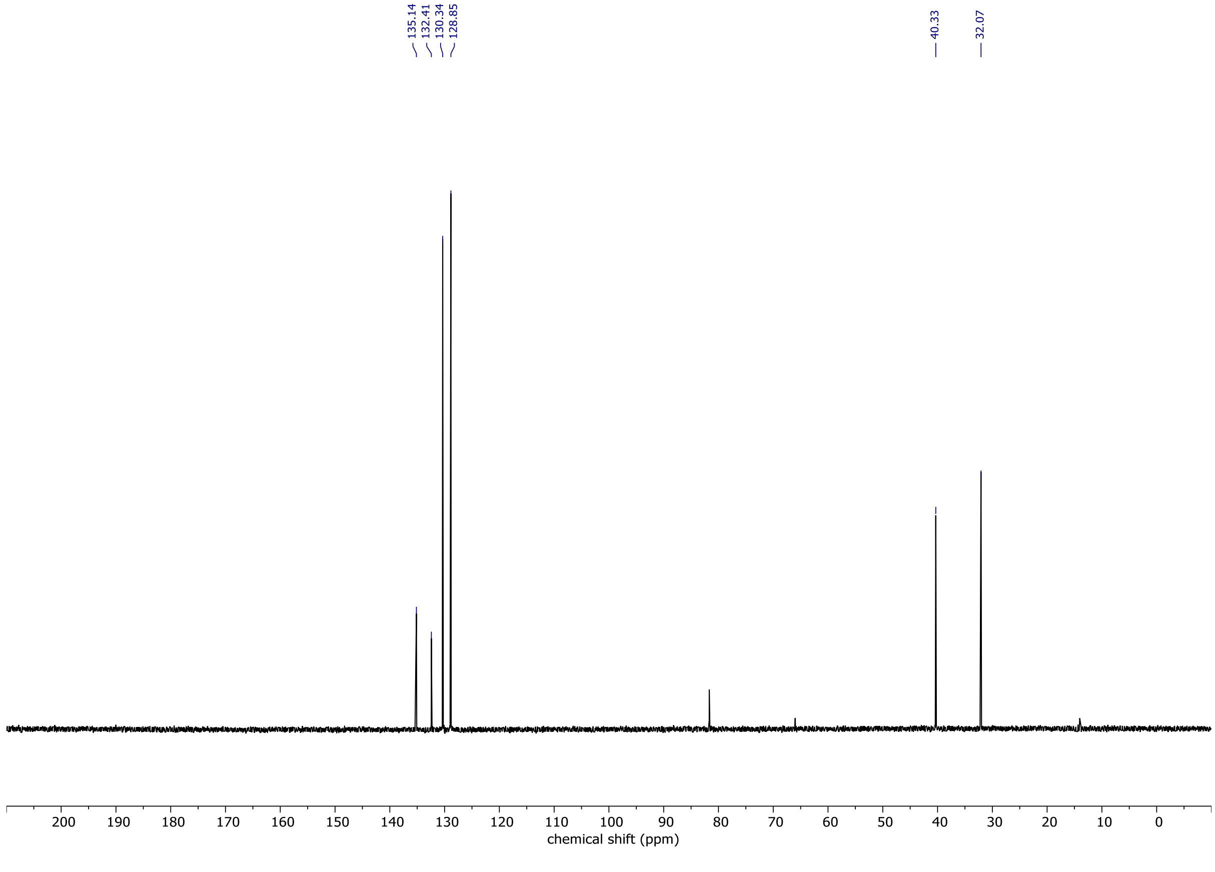

135.14
132.41
130.34
128.85
40.33
32.07
chemical shift (ppm)

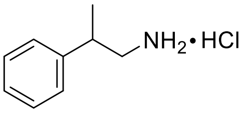
NH2•HCl

6b

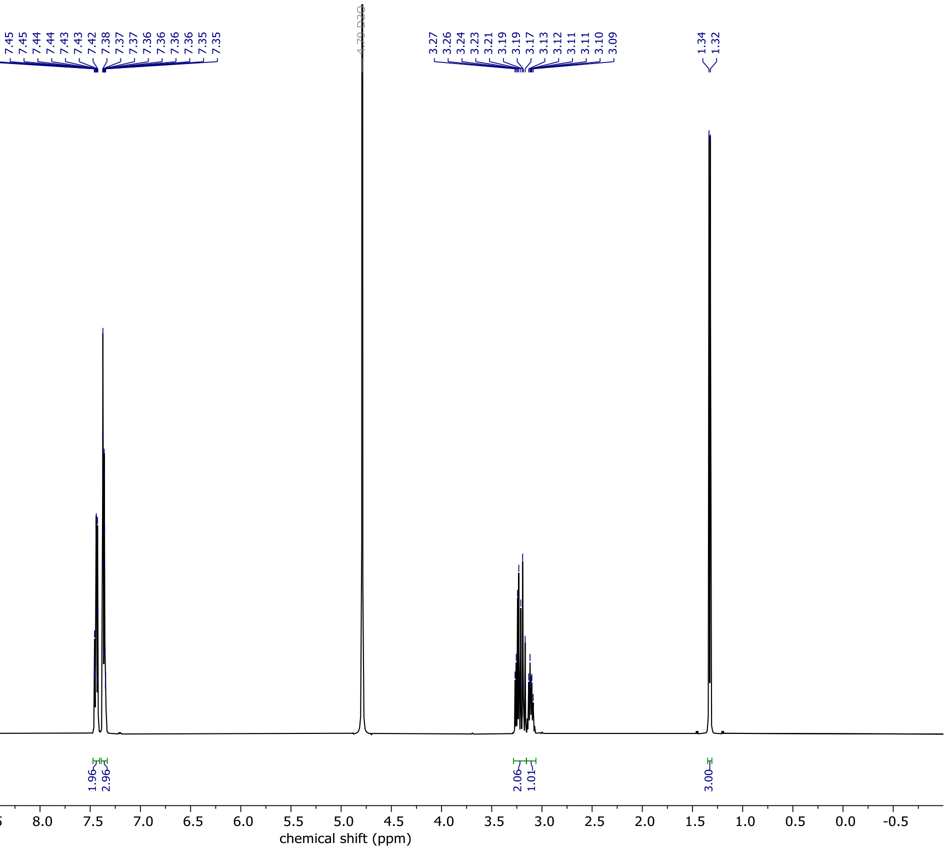
7.46
7.46
7.45
7.45
7.44
7.43
7.43
7.42
7.38
7.37
7.37
7.36
7.36
7.36
7.35
7.35
3.27
3.26
3.24
3.23
3.21
3.19
3.19
3.17
3.13
3.12
3.11
3.11
3.10
3.09
1.34
1.32
1.96
2.96
2.06
1.01
3.00
10.5 10.0 9.5 9.0 8.5 8.0 7.5 7.0 6.5 6.0 5.5 5.0 4.5 4.0 3.5 3.0 2.5 2.0 1.5 1.0 0.5 0.0 -0.5
chemical shift (ppm)

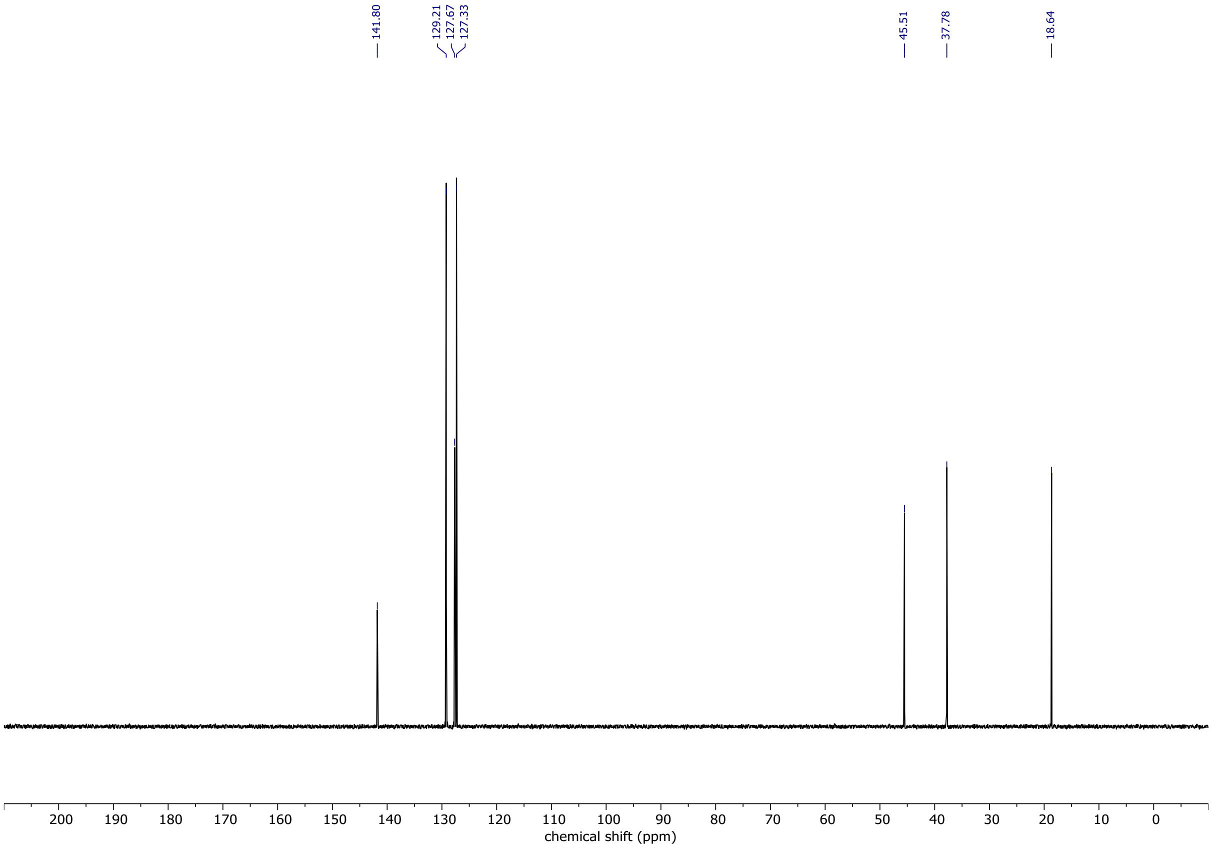
141.80
129.21
127.67
127.33
45.51
37.78
18.64
200 190 180 170 160 150 140 130 120 110 100 90 80 70 60 50 40 30 20 10 0
chemical shift (ppm)

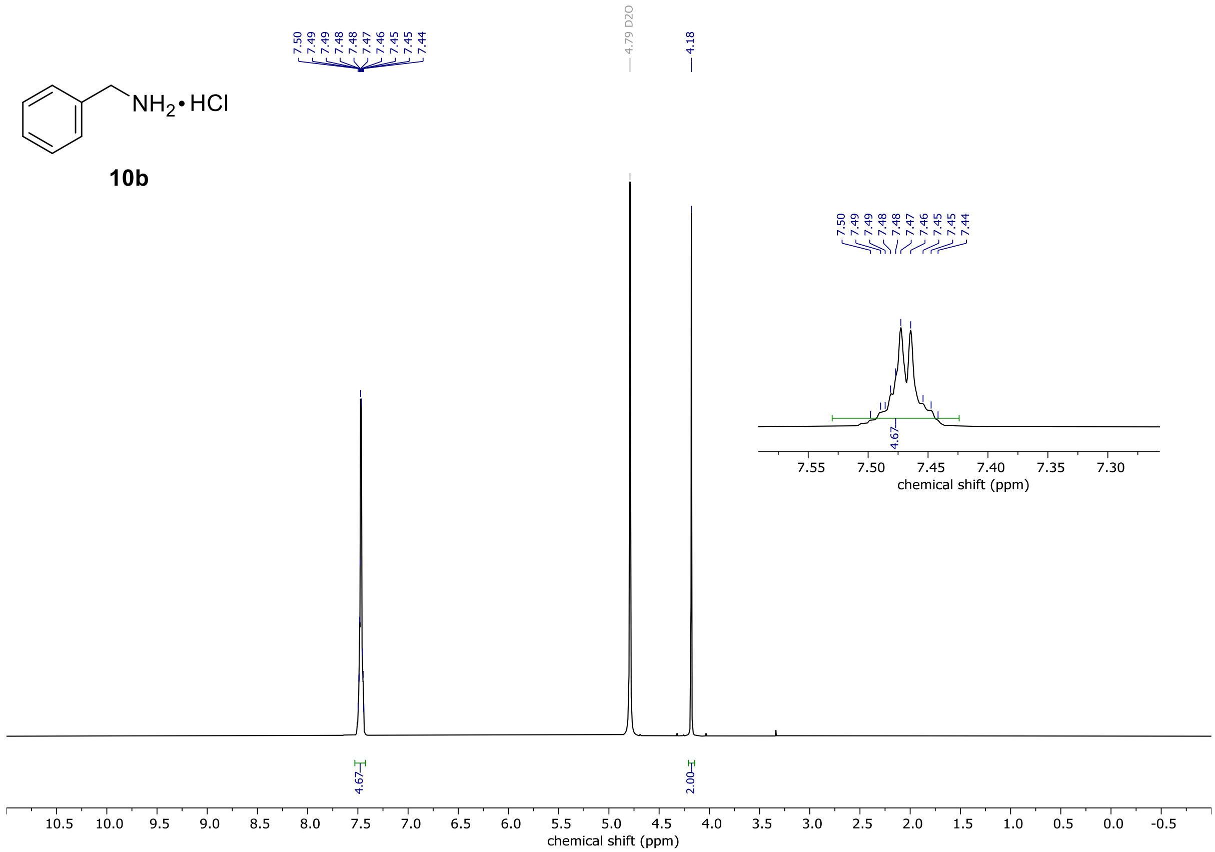
NH2•HCl
10b
7.50
7.49
7.49
7.48
7.48
7.47
7.46
7.45
7.45
7.44
4.79 D2O
4.18
4.67
2.00
chemical shift (ppm)

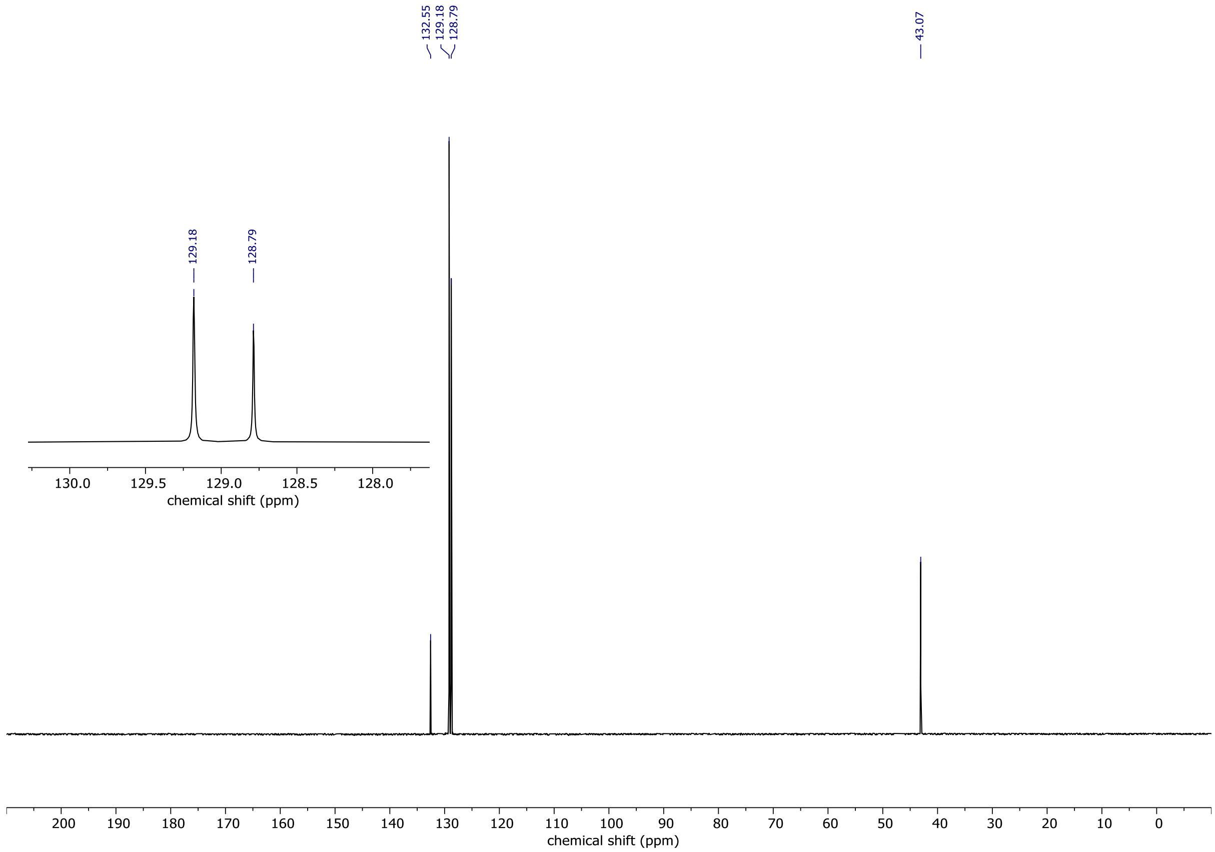
132.55
129.18
128.79
43.07
chemical shift (ppm)

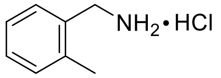
NH2•HCl

11b

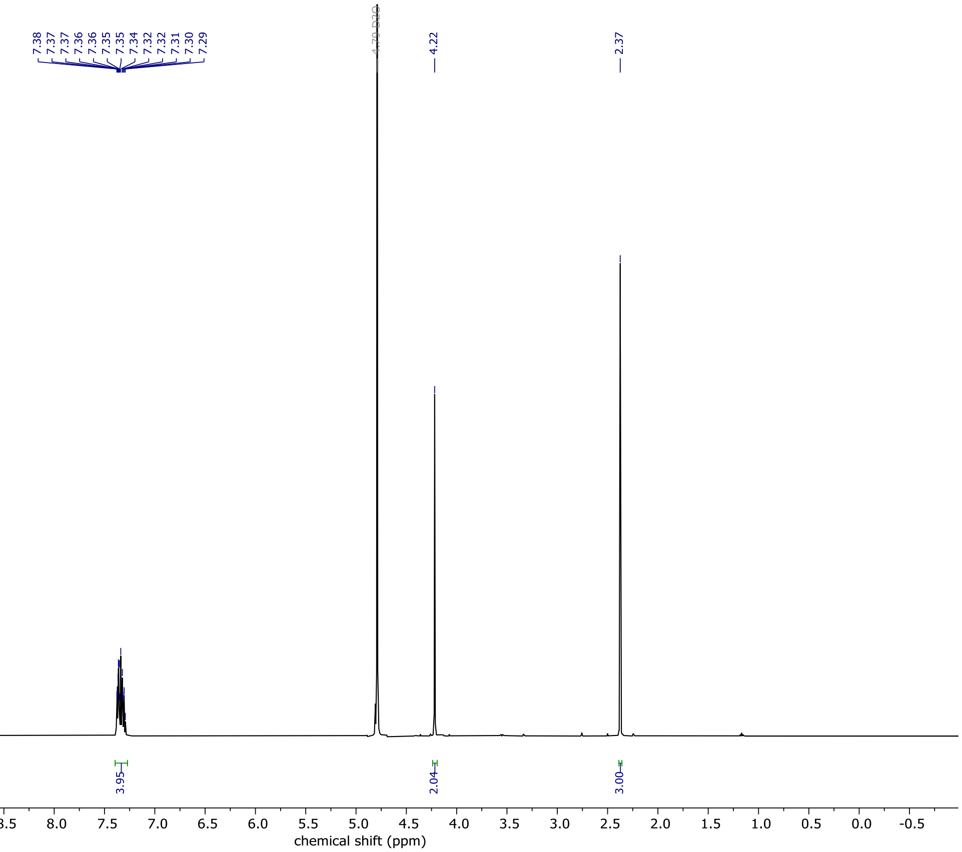
7.38
7.37
7.37
7.36
7.36
7.35
7.35
7.34
7.32
7.32
7.31
7.30
7.29
4.22
2.37
3.95
2.04
3.00
chemical shift (ppm)

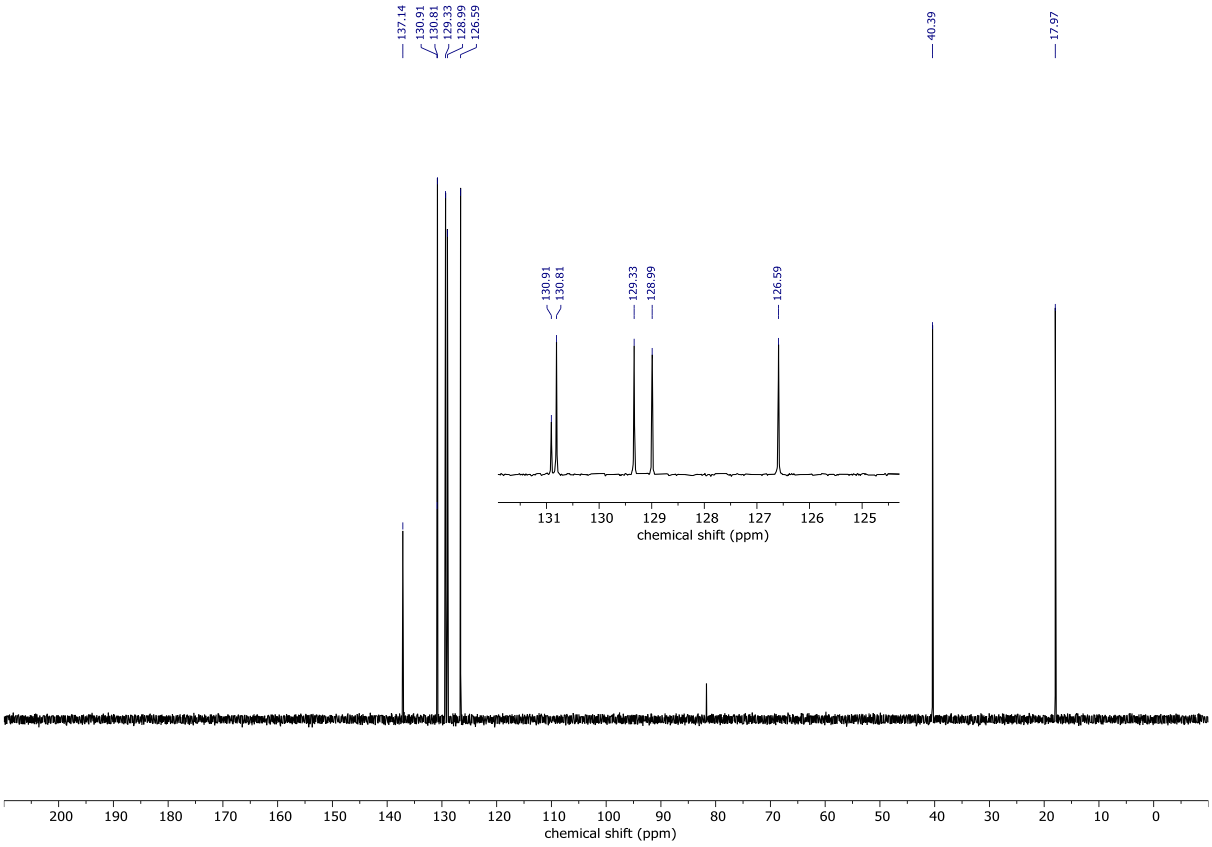
137.14
130.91
130.81
129.33
128.99
126.59
40.39
17.97
chemical shift (ppm)

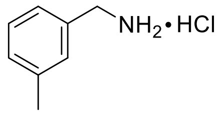
NH2•HCl

12b

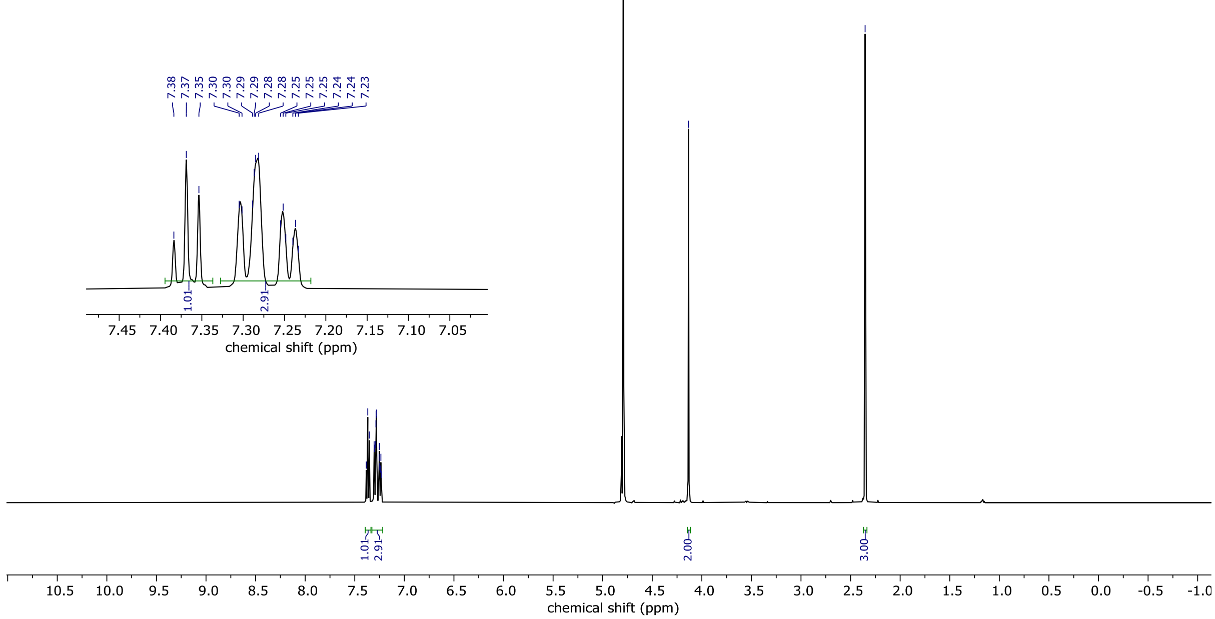
7.38
7.37
7.35
7.30
7.30
7.29
7.29
7.28
7.28
7.25
7.25
7.25
7.24
7.24
7.23
4.13
2.35
1.01
2.91
2.00
3.00
chemical shift (ppm)

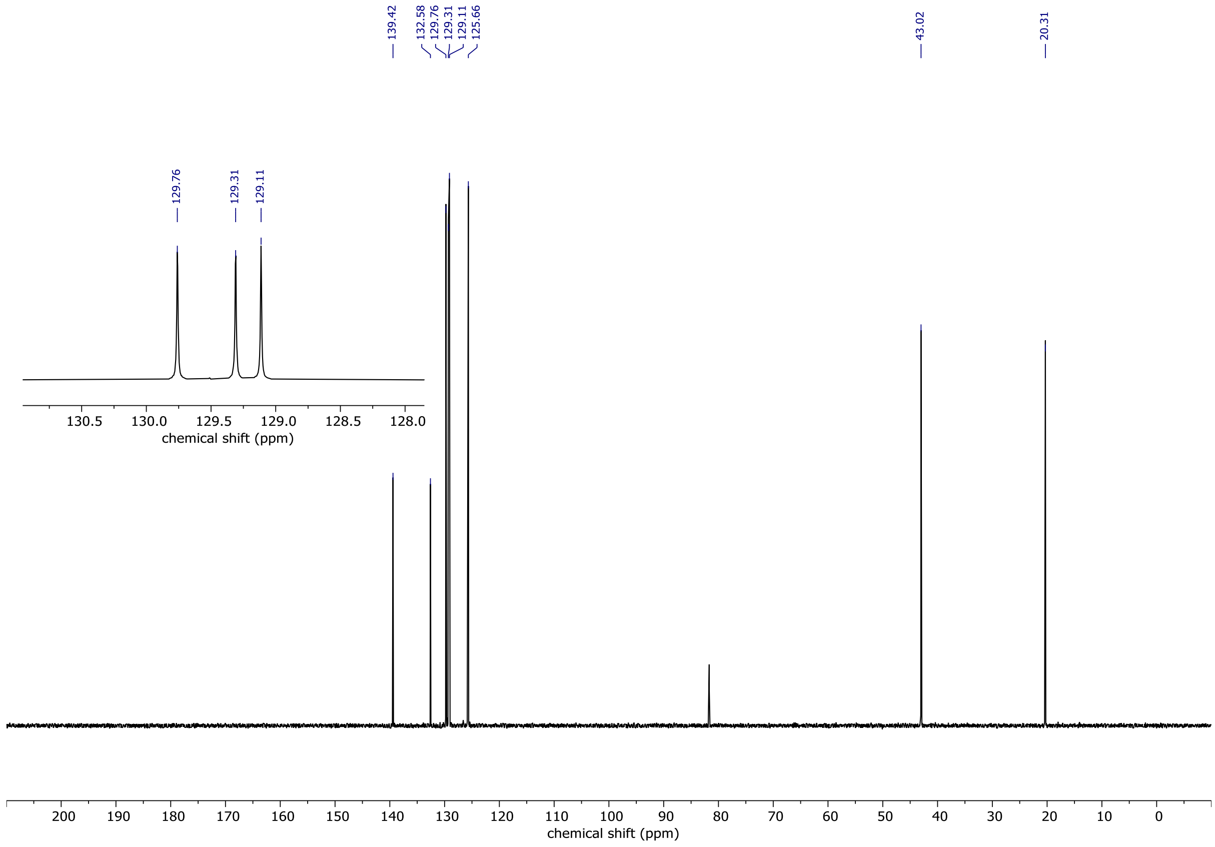
139.42
132.58
129.76
129.31
129.11
125.66
43.02
20.31
chemical shift (ppm)

13b

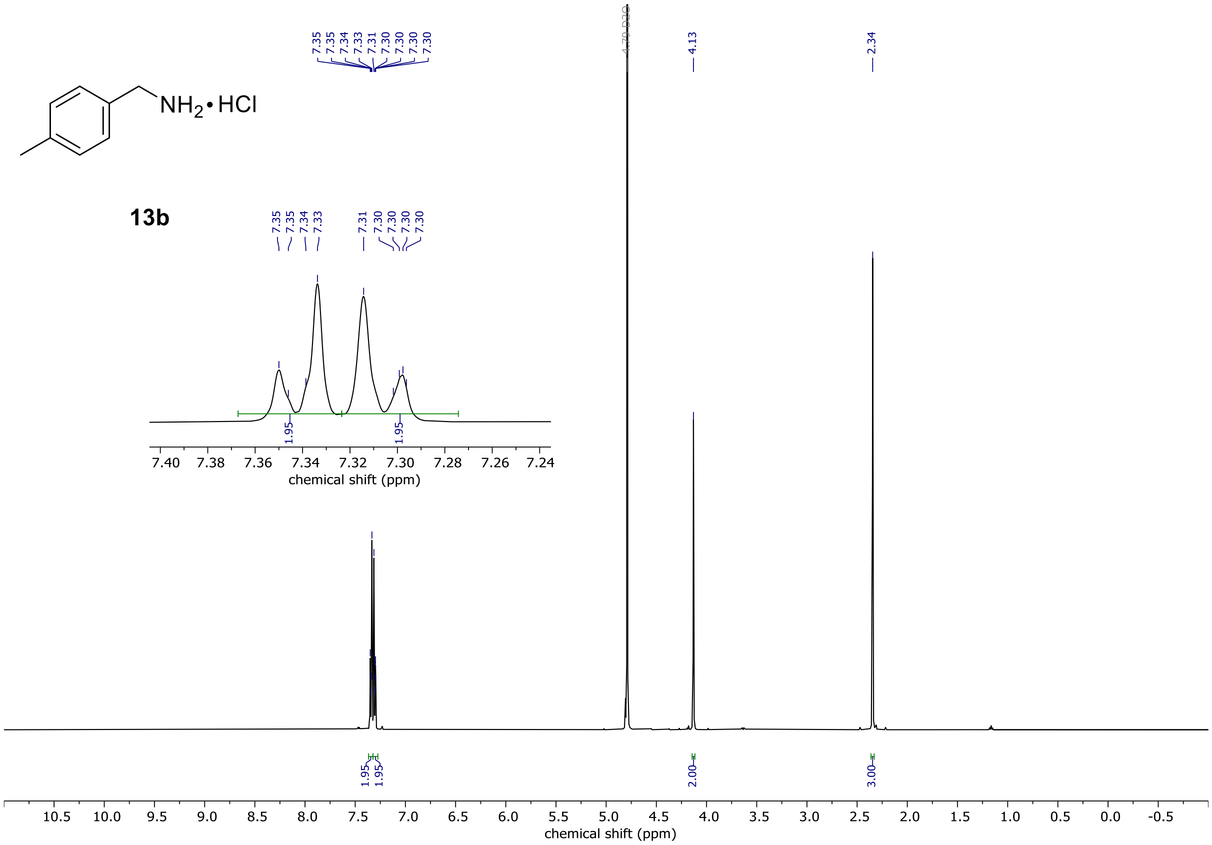
NH2•HCl
7.35
7.35
7.34
7.33
7.31
7.30
7.30
7.30
7.30
4.79 D2O
4.13
2.34
1.95
1.95
2.00
3.00
7.40 7.38 7.36 7.34 7.32 7.30 7.28 7.26 7.24
chemical shift (ppm)
10.5 10.0 9.5 9.0 8.5 8.0 7.5 7.0 6.5 6.0 5.5 5.0 4.5 4.0 3.5 3.0 2.5 2.0 1.5 1.0 0.5 0.0 -0.5
chemical shift (ppm)

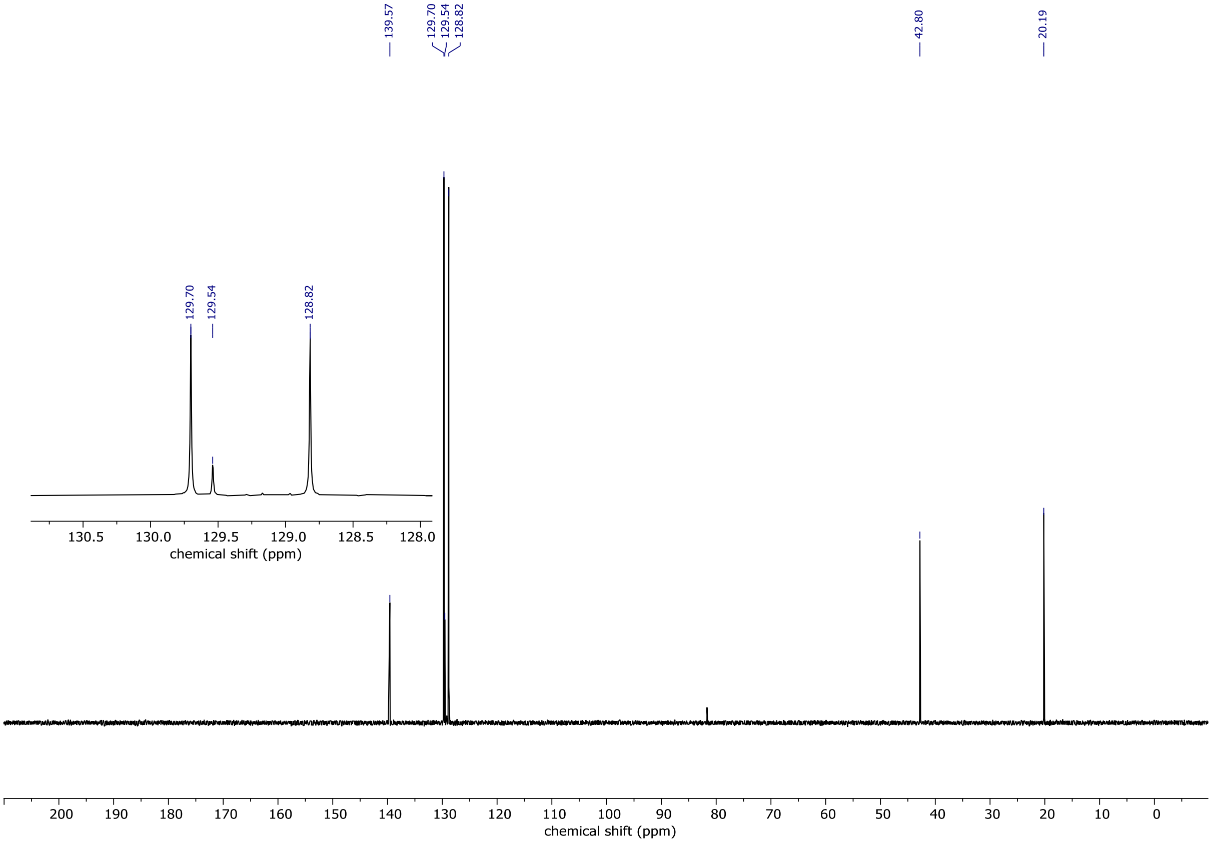
139.57
129.70
129.54
128.82
42.80
20.19
130.5 130.0 129.5 129.0 128.5 128.0
chemical shift (ppm)
200 190 180 170 160 150 140 130 120 110 100 90 80 70 60 50 40 30 20 10 0
chemical shift (ppm)

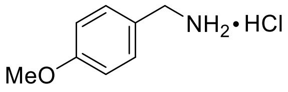

MeO
NH2•HCl

14b

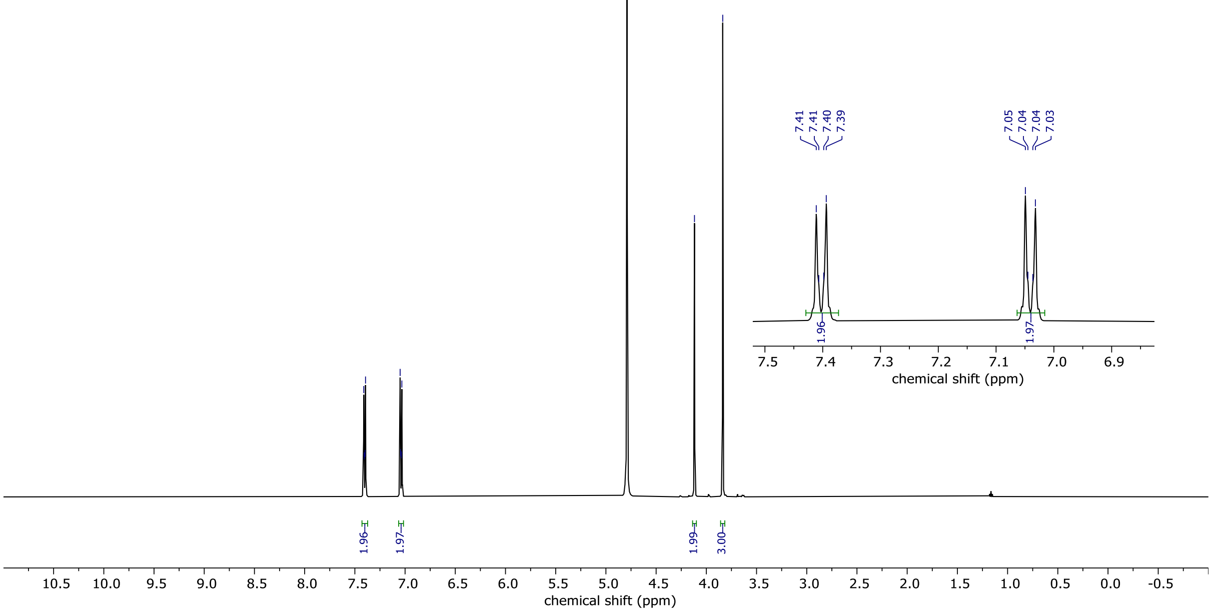

7.41
7.41
7.40
7.39
7.05
7.04
7.04
7.03
4.79 D2O
4.12
3.84
1.96
1.97
1.99
3.00
chemical shift (ppm)

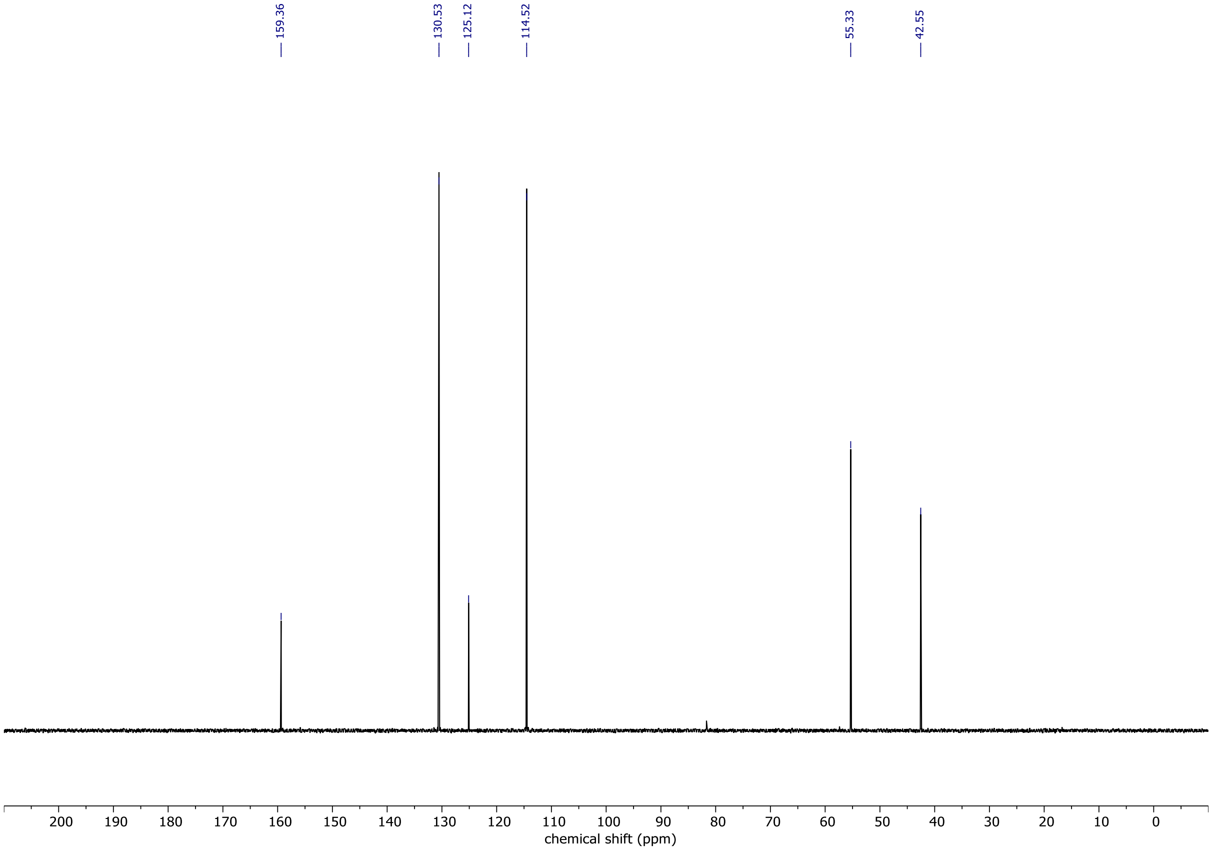

159.36
130.53
125.12
114.52
55.33
42.55
chemical shift (ppm)

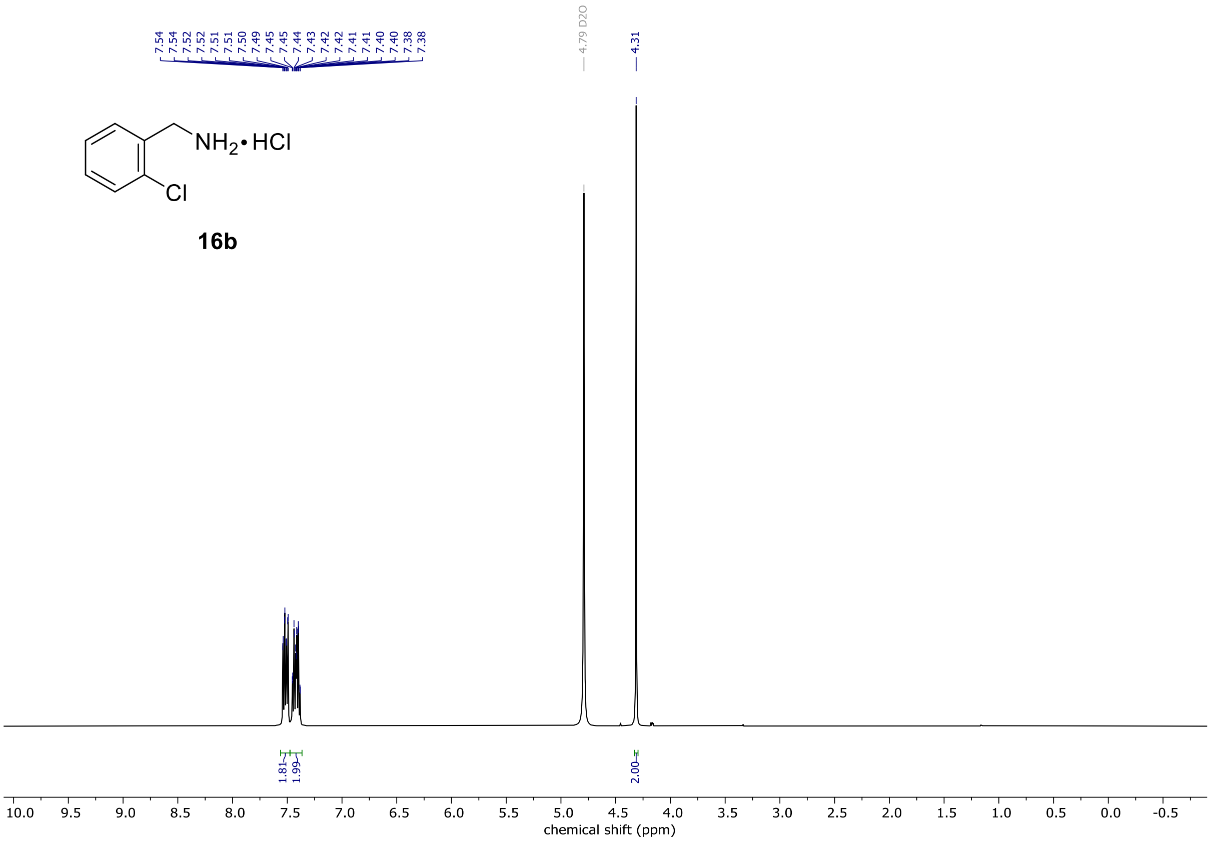

16b

7.54
7.54
7.52
7.51
7.51
7.50
7.49
7.45
7.45
7.44
7.43
7.42
7.42
7.41
7.41
7.40
7.38
7.38
4.79 D2O
4.31
1.81
1.99
2.00
10.0 9.5 9.0 8.5 8.0 7.5 7.0 6.5 6.0 5.5 5.0 4.5 4.0 3.5 3.0 2.5 2.0 1.5 1.0 0.5 0.0 -0.5
chemical shift (ppm)

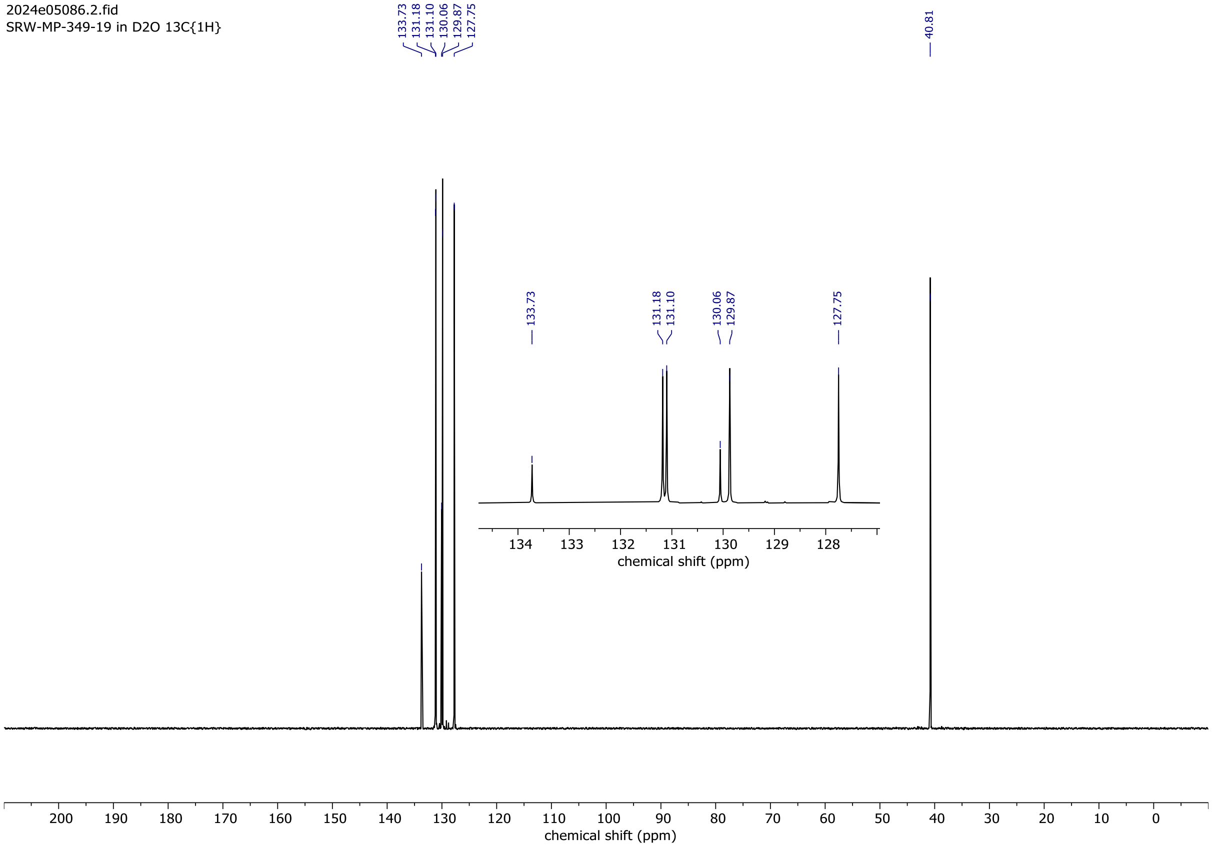
2024e05086.2.fid
SRW-MP-349-19 in D2O 13C{1H}
133.73
131.18
131.10
130.06
129.87
127.75
40.81
134 133 132 131 130 129 128
chemical shift (ppm)
200 190 180 170 160 150 140 130 120 110 100 90 80 70 60 50 40 30 20 10 0
chemical shift (ppm)

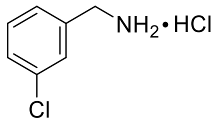
NH2•HCl
Cl

17b

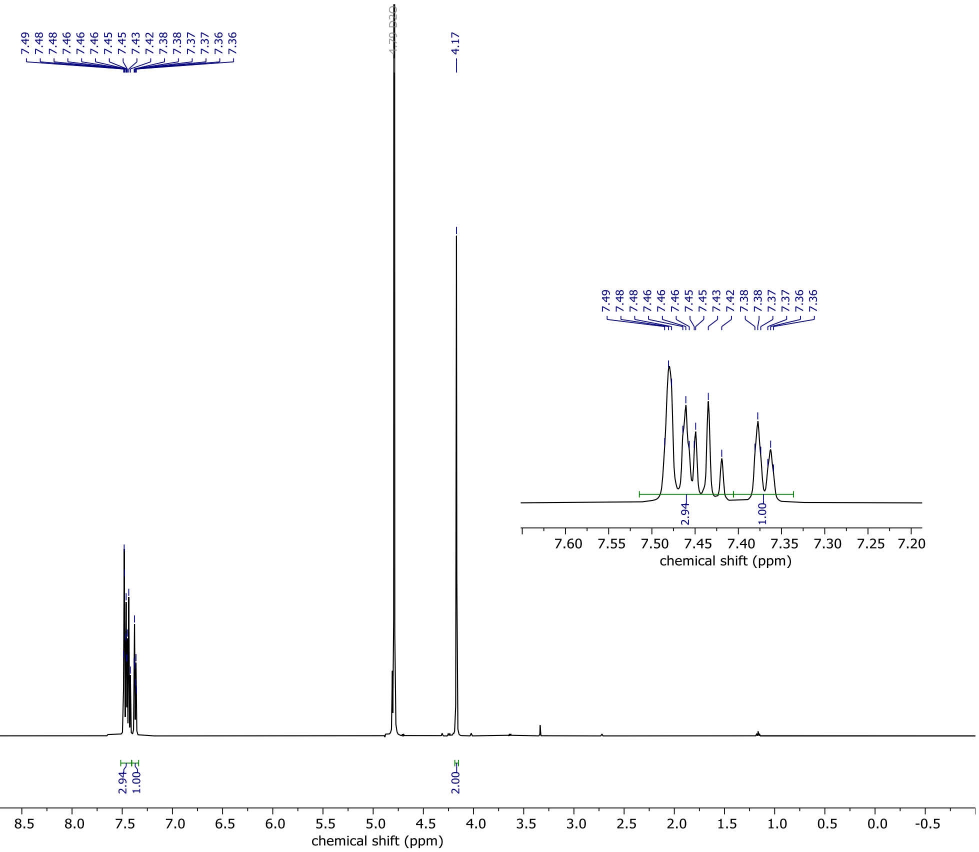
7.49 7.48 7.48 7.46 7.46 7.46 7.45 7.45 7.43 7.42 7.38 7.38 7.37 7.37 7.36 7.36
4.17
2.94
1.00
2.00
chemical shift (ppm)

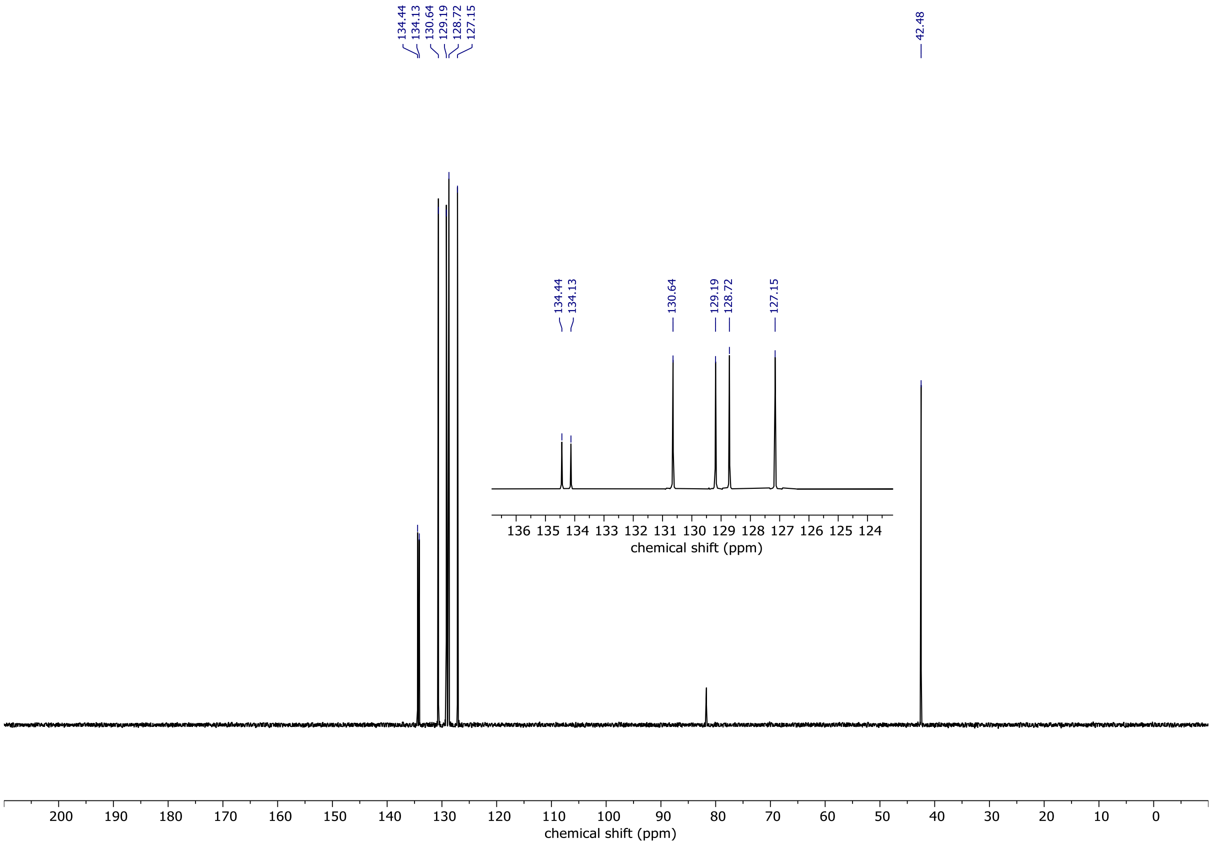
134.44 134.13 130.64 129.19 128.72 127.15
42.48
chemical shift (ppm)

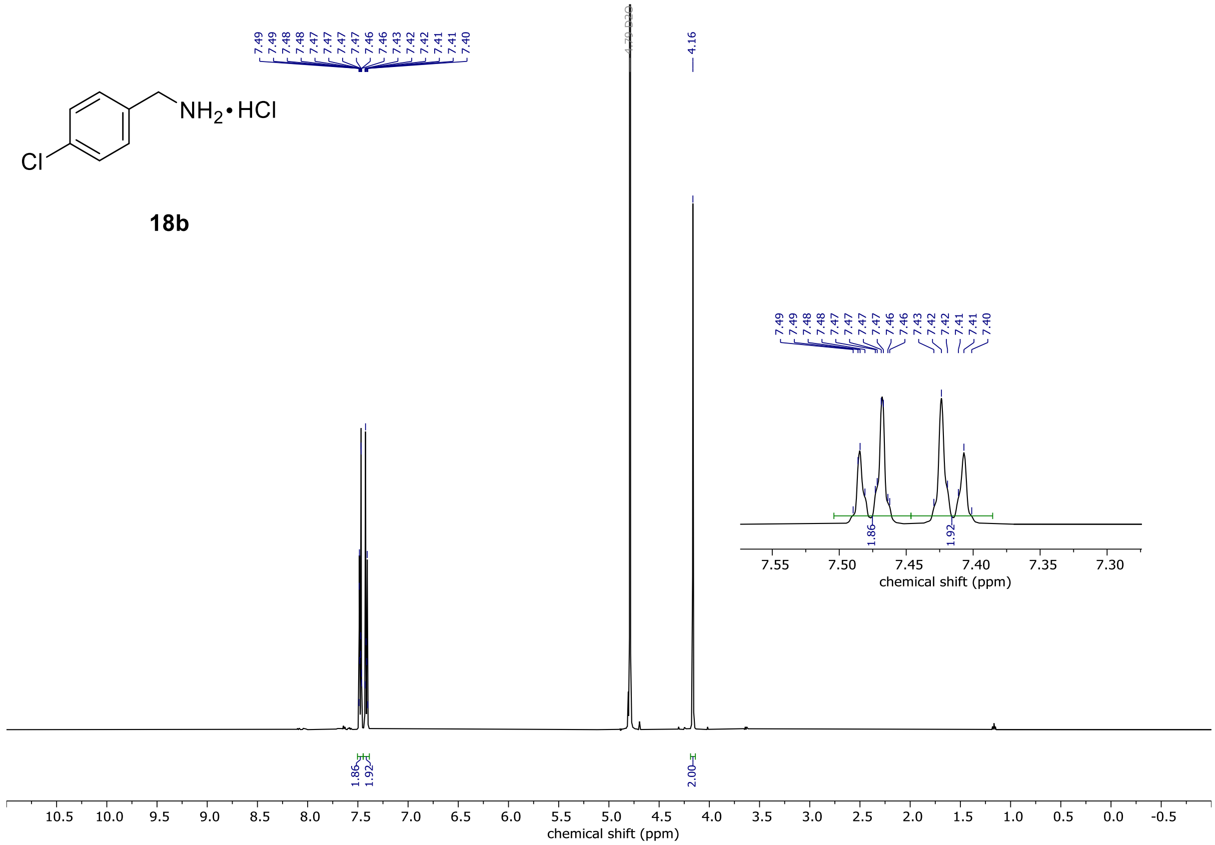
NH2 • HCl
Cl
18b
7.49 7.49 7.48 7.48 7.47 7.47 7.47 7.46 7.46 7.43 7.42 7.42 7.41 7.41 7.40
4.16
1.86
1.92
2.00
chemical shift (ppm)

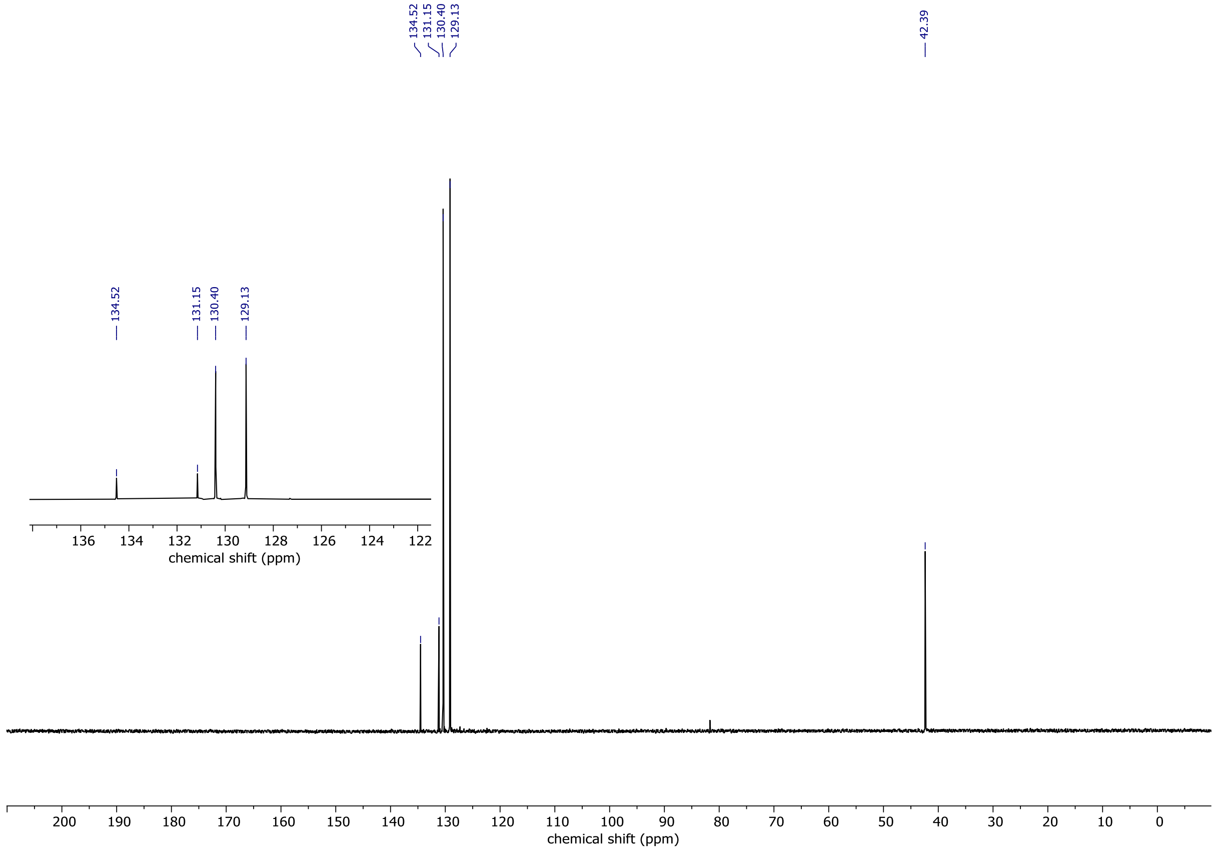
134.52 131.15 130.40 129.13
42.39
chemical shift (ppm)

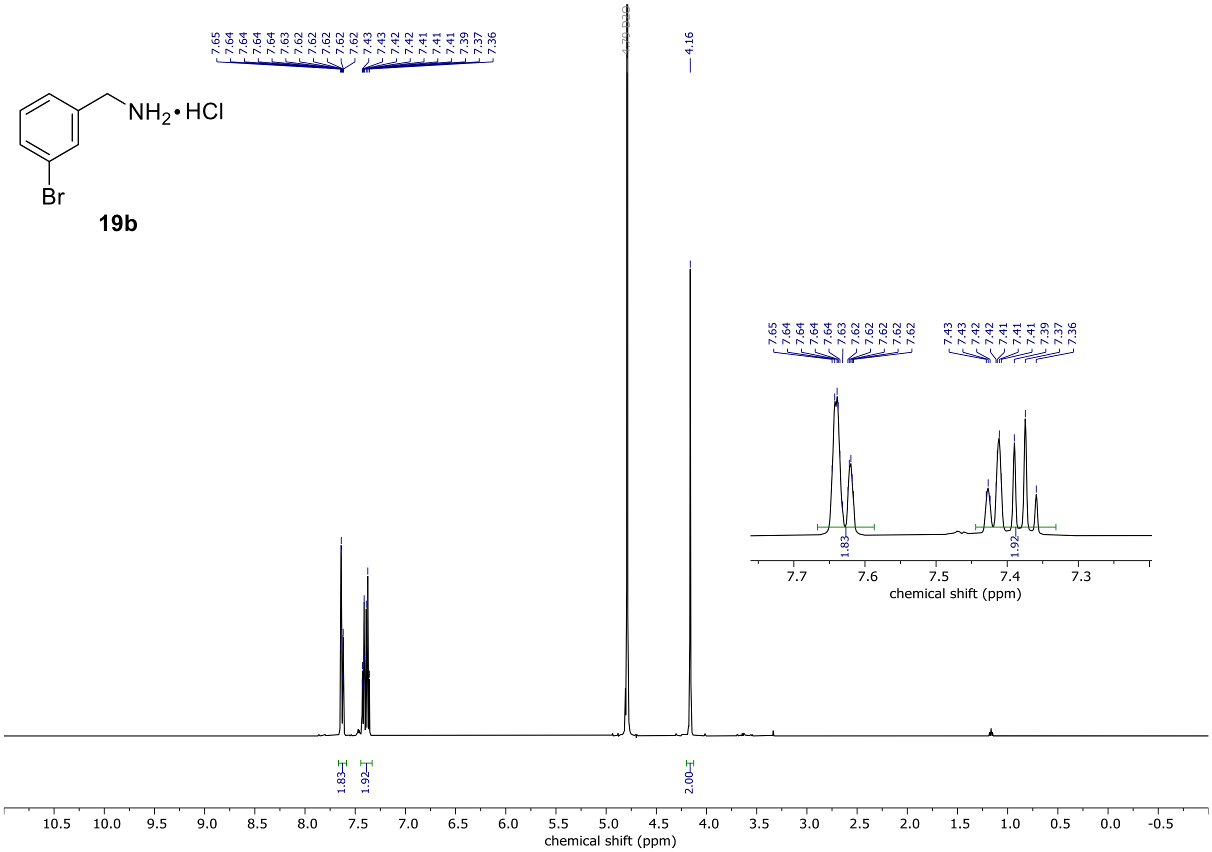

NH2•HCl
Br
19b
7.65 7.64 7.64 7.64 7.63 7.62 7.62 7.62 7.62 7.43 7.43 7.42 7.42 7.41 7.41 7.41 7.39 7.37 7.36
4.16
1.83
1.92
2.00
chemical shift (ppm)

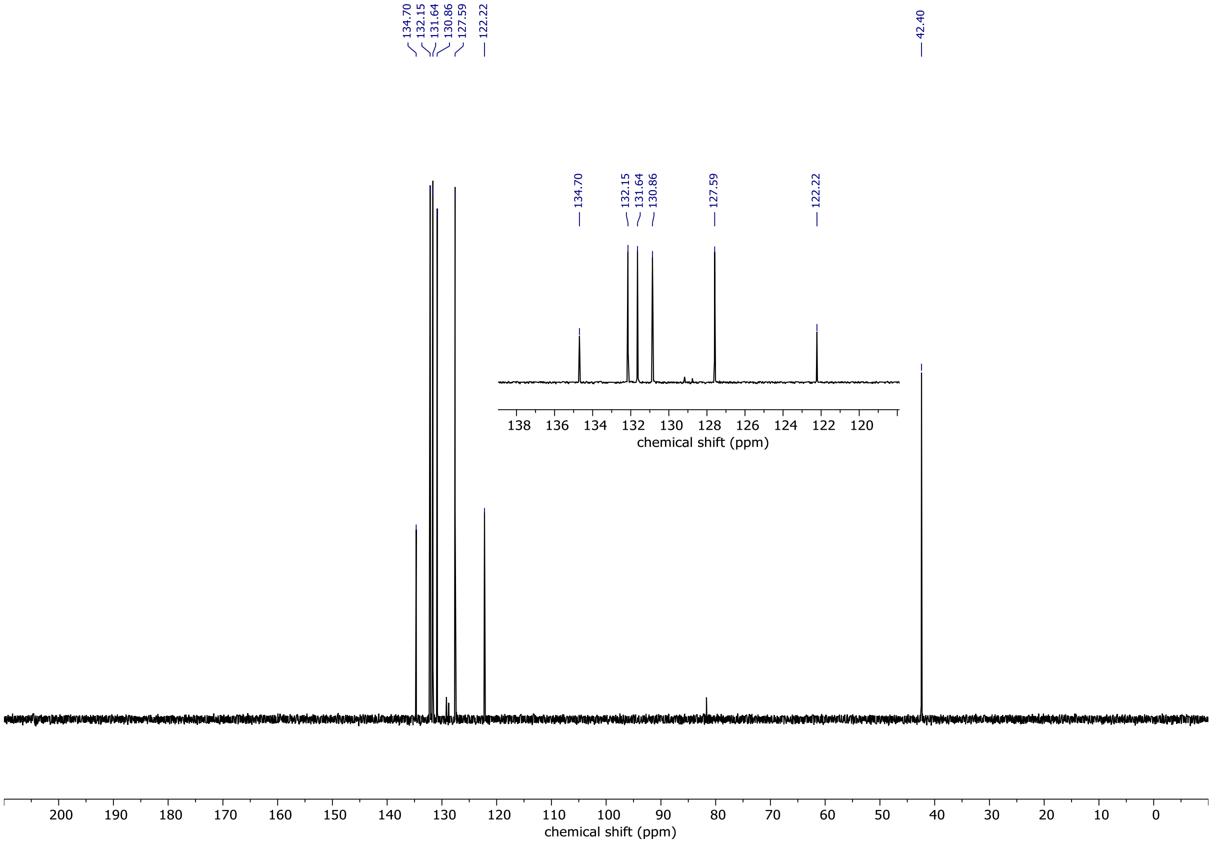

134.70 132.15 131.64 130.86 127.59 122.22
42.40
chemical shift (ppm)

10 References

(1) Goclik, L.; Walschus, H.; Bordet, A.; Leitner, W. Selective hydrodeoxygenation of acetophenone derivatives using a Fe25Ru75@SILP catalyst: a practical approach to the synthesis of alkyl phenols and anilines. *Green Chemistry* **2022**, *24* (7), 2937-2945, 10.1039/D1GC04189D. DOI: 10.1039/D1GC04189D.
(2) Sprang, F.; Schupp, N.; Kohlpaintner, P. J.; Gooßen, L. J.; Waldvogel, S. R. E-Dakin reaction: oxidation of hydroxybenzaldehydes to phenols with electrochemically generated peroxodicarbonate as sustainable ex-cell oxidizer. *Green Chemistry* **2024**, *26* (10), 5862-5868, 10.1039/D3GC04597H. DOI: 10.1039/D3GC04597H.
(3) Ma, Z.; Chandrashekhar, V. G.; Zhou, B.; Alenad, A. M.; Rockstroh, N.; Bartling, S.; Beller, M.; Jagadeesh, R. V. Stable and reusable Ni-based nanoparticles for general and selective hydrogenation of nitriles to amines. *Chemical Science* **2022**, *13* (36), 10914-10922, 10.1039/D2SC02961H. DOI: 10.1039/D2SC02961H.
(4) Constable, D. J. C.; Curzons, A. D.; Cunningham, V. L. Metrics to 'green' chemistry—which are the best? *Green Chemistry* **2002**, *4* (6), 521-527, 10.1039/B206169B. DOI: 10.1039/B206169B.
(5) Hudlicky, T. Design Constraints in Practical Syntheses of Complex Molecules: Current Status, Case Studies with Carbohydrates and Alkaloids, and Future Perspectives. *Chemical Reviews* **1996**, *96* (1), 3-30. DOI: 10.1021/cr950012g.
(6) Dai, H.; Guan, H. Switching the Selectivity of Cobalt-Catalyzed Hydrogenation of Nitriles. *ACS Catalysis* **2018**, *8* (10), 9125-9130. DOI: 10.1021/acscatal.8b02645.
(7) Lewin, A. H.; Navarro, H. A.; Wayne Mascarella, S. Structure–activity correlations for β-phenethylamines at human trace amine receptor 1. *Bioorganic & Medicinal Chemistry* **2008**, *16* (15), 7415-7423. DOI: https://doi.org/10.1016/j.bmc.2008.06.009.
(8) Lenstra, D. C.; Lenting, P. E.; Mecinović, J. Sustainable organophosphorus-catalysed Staudinger reduction. *Green Chemistry* **2018**, *20* (19), 4418-4422, 10.1039/C8GC02136H. DOI: 10.1039/C8GC02136H.
(9) Hothi, P.; Hay, S.; Roujeinikova, A.; Sutcliffe, M. J.; Lee, M.; Leys, D.; Cullis, P. M.; Scrutton, N. S. Driving Force Analysis of Proton Tunnelling Across a Reactivity Series for an Enzyme-Substrate Complex. *ChemBioChem* **2008**, *9* (17), 2839-2845. DOI: https://doi.org/10.1002/cbic.200800408.
(10) Kitamura, M.; Suga, T.; Chiba, S.; Narasaka, K. Synthesis of Primary Amines by the Electrophilic Amination of Grignard Reagents with 1,3-Dioxolan-2-one O-Sulfonyloxime. *Organic Letters* **2004**, *6* (24), 4619-4621. DOI: 10.1021/ol0479951.
(11) Sanagawa, A.; Nagashima, H. Hydrosilane Reduction of Nitriles to Primary Amines by Cobalt–Isocyanide Catalysts. *Organic Letters* **2019**, *21* (1), 287-291. DOI: 10.1021/acs.orglett.8b03736.
(12) Elangovan, S.; Topf, C.; Fischer, S.; Jiao, H.; Spannenberg, A.; Baumann, W.; Ludwig, R.; Junge, K.; Beller, M. Selective Catalytic Hydrogenations of Nitriles, Ketones, and Aldehydes by Well-Defined Manganese Pincer Complexes. *Journal of the American Chemical Society* **2016**, *138* (28), 8809-8814. DOI: 10.1021/jacs.6b03709.
(13) Guan, Q.; Jiang, M.; Wu, J.; Zhai, Y.; Wu, Y.; Bao, K.; Zhang, W. Direct cycle between co-product and reactant: an approach to improve the atom economy and its application in the synthesis and protection of primary amines. *Green Chemistry* **2016**, *18* (21), 5794-5799, 10.1039/C6GC01318J. DOI: 10.1039/C6GC01318J.
